# Virtual Reality Interventions for Stress Reduction in the General Population: Systematic Review and Meta-Analysis of Randomized Controlled Trials

**DOI:** 10.2196/78212

**Published:** 2026-05-25

**Authors:** Hannah Strauch, Isabel Schuil, Stefan Simm, Jens Grubert, Snehanjali Kalamkar, Karin Meissner

**Affiliations:** 1 Faculty of Applied Natural Sciences and Health, Coburg University of Applied Sciences and Arts, Friedrich-Streib-Straße 2, Coburg, 96450, Germany, 49 9561 317 0; 2 Faculty of Electrical Engineering and Computer Sciences, Coburg University of Applied Sciences and Arts, Coburg, Germany

**Keywords:** virtual reality, VR, immersion, stress reduction, mental health, positive emotion, general population, stress management, predictor, effectiveness, meta-analysis, systematic review, PRISMA

## Abstract

**Background:**

Increasing mental demands across multiple life domains underscore the importance of effective individual stress management to mitigate the adverse health consequences of chronic stress. Growing evidence suggests that virtual reality (VR) interventions constitute an effective approach to stress reduction.

**Objective:**

This systematic review and meta-analysis aimed to examine and compare application areas of VR interventions for stress reduction in the general population and to identify potential predictors of effectiveness based on sample characteristics and intervention design.

**Methods:**

Five databases (MEDLINE, CINAHL, CENTRAL, PsycInfo, and Web of Science) were systematically searched for randomized controlled trials investigating the effectiveness of VR interventions for stress reduction in the general population. Studies were included if they primarily focused on stress reduction, included a neutral control condition, and reported a validated measurement of perceived stress. Trials targeting mental disorders or those conducted in the context of medical procedures were excluded. Two reviewers independently screened the literature, extracted data, and assessed the risk of bias using the Cochrane Collaboration’s tool. Effects were synthesized using pooled standardized mean differences, and relevant predictors were evaluated through subgroup analyses and meta-regressions.

**Results:**

A total of 55 relevant studies met the inclusion criteria, with 37 investigating single-session and 18 multisession interventions (ranging from 1 to 42 sessions over 2 days to 6 months). The meta-analysis included 39 studies with 4024 participants (sample sizes 24‐409; mean ages 19.2‐70.6 years). Intervention types included VR-based nature exposure (21), biophilic architectural elements (6), guided meditation (9), interactive tasks (4), and other approaches (1). On average, VR interventions significantly reduced perceived stress level (−0.55, 95% CI −0.70 to −0.40; *P*<.001; *I*^2^=76%; 95% prediction interval [PI] −1.32 to 0.23), anxiety (−0.88, 95% CI −1.23 to −0.54; *P*<.001; *I*^2^=87%; 95% PI −2.07 to 0.31), and depression (−0.34, 95% CI −0.47 to −0.21; *P*<.001; *I*^2^=0%; 95% PI −0.47 to −0.21), and enhanced positive emotion (−0.64, 95% CI −0.84 to −0.46; *P*<.001; *I*^2^=62%; 95% PI −1.28 to −0.02). In addition, the decrease in systolic blood pressure was significant (−0.20, 95% CI −0.37 to −0.04; *P*=.02; 95% PI −0.37 to −0.04). Univariate linear regression analyses identified multiple sessions (*R*²=15%, *P*=.02) as a significant predictor of VR-based stress reduction. Further analyses of technical and content-related characteristics revealed that a higher image refresh rate (*t*_15_=−2.36, 2-tailed; *P*=.04) was associated with stronger intervention effects.

**Conclusions:**

This review highlights the considerable potential of VR interventions for reducing psychological stress in the general population and extends recent evidence by quantifying the effects of a broad range of VR-based stress reduction approaches and, for the first time, comparing diverse intervention characteristics. VR represents a promising alternative, particularly in contexts where access to real-life nature or conventional relaxation methods is limited or resource-intensive.

## Introduction

Stress can influence individual health in multiple ways. In general, stress is defined as an organism’s reaction to diverse stimuli categorized as harmful or straining by the brain [1]. These stressors can be temporary or recurring and result in an activation of the hormonal and nervous system leading to physical, cognitive, and sociopsychological adjustments [1,2]. While acute stress, for example, time pressure, can have beneficial effects on performance and cognition, chronic stress, characterized by ongoing demands without adequate recovery, is associated with negative consequences for both mental (eg, sleeping or memory issues) and physical health (eg, hypertension). These effects may occur indirectly, by affecting health behavior [3], as well as direct risk factors for several diseases such as cancer, stroke, anxiety disorder, or dementia [2].

The prevalence of both general mental health issues, such as negative emotions, worries, or stress, and mental disorders, such as depression, has increased in European countries over time [4,5]. Global crises, for example, the COVID-19 pandemic, the ongoing climate change, and various armed conflicts, act as collective stressors, negatively affecting the mental well-being of the general population [4,6,7]. In addition, individuals encounter unique challenges that can affect their perceived stress levels (PSLs). According to the Flash Eurobarometer 530, a representative survey on mental health commissioned by the European Commission and conducted across 27 EU countries among citizens over the age of 15 years in 2023, 46% of the participants reported experiencing emotional or psychological problems in the previous 12 months [5]. Among these, 69% reported feeling sad or down, 50% experienced excessive fears and worries, and 34% struggled with daily stress.

The current rise in mental load and its detrimental effects on psychological and physiological health highlight the need for effective strategies to help individuals in coping with daily demands and reducing PSL. According to the Flash Eurobarometer 530, mental health has become a major public concern [5]. The majority of respondents either “totally agreed” (56%) or “tended to agree” (33%) that mental health promotion is just as important as the promotion of physical health. There is strong evidence that stress management techniques, such as mindfulness-based stress reduction [8], progressive muscle relaxation [9], and autogenic training [10], can help individuals in coping with stressful situations and prevent negative health consequences [11]. In addition, exposure to nature has been shown to be an effective method for reducing stress [12,13]. Enhancing scientific evidence, the Flash Eurobarometer 530 found that 35% of the respondents experience mental health benefits from contact with nature and green spaces, while 27% indicated that relaxation techniques improve their mental well-being [5].

Over the past years, virtual reality (VR) has gained significant attention as a promising technology in various health care domains [14,15]. As the quality and accessibility of VR applications are steadily improving, the number of publications on its potential health benefits has noticeably increased. The possibility to expose users to specific virtual environments or situations has given rise to two major fields of VR research and application: the utilization as an alternative and location-independent treatment for mental disorders and, since 2019, as a tool for individual health promotion, including stress reduction and meditation [15,16]. Common application areas with proven effectiveness include VR exposure therapy for severe anxiety disorders [17], such as social anxiety disorder [18,19], posttraumatic stress disorder [20,21], and other psychiatric diagnoses [22,23]. In addition to the therapy of specific disorders, systematic reviews provide evidence for the stress-reducing effects of VR-based relaxation and stress management interventions for both individuals with mental health conditions [24,25] and the general population [26-28].

Recognizing stress management as a key factor in overall health, considerable efforts have been made to translate proven stress reduction techniques into VR, with the aim of creating cost-effective and easily accessible alternatives [24]. For instance, research has demonstrated the efficacy of VR-based stress management based on the exposure to natural environments [29], breathing exercises [30], and mindfulness interventions [26]. Evidence also supports the stress-reducing effects of these interventions in specific target groups and settings, including patients with cancer [31] and cardiovascular disease [32], students and young adults [28], and workplace environments [33]. Overall, current research highlights the potential of VR for stress reduction for specific application areas. Nevertheless, given the heterogeneity of stressors and challenges among different target groups, there is a corresponding variety in requirements and treatment goals for VR-based stress interventions. As most recent reviews are limited to specific target groups (eg, patients with psychiatric disorders [17-20] or young adults [28]) or interventions (eg, nature exposure [29,34] or breathing interventions [30]), a broader meta-analysis comparing different VR intervention approaches regarding their effectiveness for separate target populations is lacking. The effectiveness of these interventions may be influenced by factors such as intervention design, VR content, and technological features, as well as individual characteristics including age, sex, prior experiences, and impairments [14,27,35].

As demonstrated, VR-based interventions have been shown to be effective in reducing PSL in the general population. When daily stress becomes more common among the general population, easily applicable tools for stress reduction for everyone become important for global health promotion. Consequently, the effects of VR interventions for stress reduction, especially in the general population, should be investigated in more detail. To exploit the potential of VR as an easily accessible and purposive method for individual self-practiced stress reduction and to create expedient VR interventions addressing individual requirements and conditions, it is crucial to investigate predictors that enhance stress-reducing effects for different target groups. Therefore, the objective of this systematic review was to synthesize the most common application areas and intervention characteristics in order to quantify and compare the effectiveness of VR for stress reduction across various settings and target groups by meta-analyses. As other studies focus on the general effectiveness of VR interventions in distinct target populations and the predictors of an enhanced effectiveness in stress reduction remain largely underexplored [28-30], this meta-analysis focused on differences in the effectiveness of VR-based interventions for reducing PSL, comparing subgroups based on sociodemographic characteristics (eg, age, sex, or prior VR experience) and across various application areas (eg, clinical settings, workplaces, universities, and the general population). Furthermore, this meta-analysis exploratively investigated which technical features and methodological design aspects of VR interventions could influence and predict their effectiveness in reducing PSL, so that further VR approaches can be designed more expediently, resulting in more successful VR applications for stress reduction.

## Methods

### Guidelines and Register

This systematic review was conducted and reported in accordance with the PRISMA (Preferred Reporting Items for Systematic Reviews and Meta-Analyses) guidelines [36], the PRISMA-S (Extension to the PRISMA Statement for Reporting Literature Searches in Systematic Reviews) guidelines [37], and the GRADE (Grading of Recommendations Assessment, Development, and Evaluation) approach [38]. The study protocol was preregistered in PROSPERO (CRD42024592600).

### Literature Search

A systematic search of multiple databases was conducted (HS) on July 24, 2024, to identify randomized controlled trials (RCTs) of immersive VR-based interventions for stress reduction in the general population. The information sources searched included the databases MEDLINE PubMed, CINAHL EBSCOhost, the Cochrane Controlled Trials Register (CENTRAL), PsycInfo EBSCOhost, and Web of Science. Study registries, citation searching, or further resources were not considered. The search strings combined different keywords and text terms related to VR and stress, completed by validated filters [39] for RCTs. There were no further limits applied, for example, for date or language. Search strategies were peer reviewed by a second researcher (KM) regarding PRESS (Peer Review of Electronic Search Strategies) guidelines [40]. The search string used for MEDLINE is provided as an example in Table 1, and the complete search strategies for each database are provided in Multimedia Appendix 1. The search was updated on February 13, 2026, using the same strategy with publication date filters applied.

**Table 1.** Search strategy of the systematic search for the systematic review and meta-analysis on virtual reality interventions for stress reduction in the database MEDLINE.

No.	Search terms
#1	“virtual reality”[MeSH Terms]
#2	“virtual reality”[All Fields]
#3	“virtual reality”[Title/Abstract:~2] or “simulated reality”[Title/Abstract:~2] or “digital reality”[Title/Abstract:~2] or “spatial reality”[Title/Abstract:~2]
#4	“virtual environment”[Title/Abstract:~2] or “simulated environment”[Title/Abstract:~2] or “immersive environment”[Title/Abstract:~2] or “digital environment”[Title/Abstract:~2]
#5	“virtual nature”[Title/Abstract:~2] or “simulated nature”[Title/Abstract:~2] or “digital nature”[Title/Abstract:~2] or “computer-generated nature”[Title/Abstract:~2]
#6	“virtual world”[Title/Abstract:~2] or “simulated world”[Title/Abstract:~2] or “immersive world”[Title/Abstract:~2] or “computer-generated world”[Title/Abstract:~2]
#7	“virtual spaces”[Title/Abstract:~2] or “3D environment”[Title/Abstract:~2] or “virtual immersion”[Title/Abstract:~2] or “immersive technology”[Title/Abstract:~2] or “3D simulation”[Title/Abstract:~2] or “3D video”[Title/Abstract:~2] or “360 degree simulation”[Title/Abstract:~2] or “360 degree video”[Title/Abstract:~2] or “virtual exposure”[Title/Abstract:~2] or “virtual experience”[Title/Abstract:~2] or “immersive experience”[Title/Abstract:~2] or “simulated experience”[Title/Abstract:~2]
#8	1 or 2 or 3 or 4 or 5 or 6 or 7
#9	“stress, psychological”[MeSH Terms] or “subjective stress”[MeSH Terms] or “relaxation”[MeSH Terms]
#10	“stress*“[Title/Abstract] or “distress”[Title/Abstract] or “coping”[Title/Abstract] or “relax*“[Title/Abstract] or “resilience”[Title/Abstract] or “restorat*“[Title/Abstract] or “meditat*“[Title/Abstract] or “positive affect”[Title/Abstract] or “negative affect”[Title/Abstract]
#11	9 or 10
#12	“randomized controlled trial”[Publication Type] or “controlled clinical trial”[Publication Type]
#13	“placebo”[Title/Abstract] or “random*“[Title/Abstract] or “trial”[Title/Abstract] or “within subject*“[Title/Abstract] or “between subject*“[Title/Abstract] or “drug therapy”[MeSH Subheading]
#14	“control*“[Title/Abstract] or “comparison”[Title/Abstract] or “compare*“[Title/Abstract] or “study”[Title/Abstract] or “different”[Title/Abstract] or “experiment*"[Title/Abstract]
#15	“participant*“[Title/Abstract] or “patient*“[Title/Abstract] or “group*“[Title/Abstract] or “intervention*“[Title/Abstract] or “individual*“[Title/Abstract] or “subject*“[Title/Abstract] or “therapy”[Title/Abstract] or “adult*"[Title/Abstract]
#16	14 and 15
#17	12 or 13 or 16
#18	8 and 11 and 17
#19	“animals”[MeSH Terms]
#20	“humans”[MeSH Terms]
#21	19 not 20
#22	“review”[Publication Type] or “meta-analysis”[Publication Type]
#23	21 or 22
#24	18 not 23

### Eligibility Criteria

Inclusion was limited to RCTs that used either a within-subjects or between-subjects design and were published in English or German. Study protocols were excluded. Included studies were required to focus on human participants aged 18 years or older without any psychiatric diagnoses (eg, anxiety disorder, posttraumatic stress disorder, or depression) due to special demands regarding mental health. No further restrictions were applied regarding demographics such as gender, age, income, or intervention setting (eg, workplace or university, clinical or private setting). This review exclusively encompassed interventions based on VR with a primary focus on stress reduction, delivered through immersive technologies such as cave automatic virtual environments (CAVE) or head-mounted displays (HMD). In this context, a CAVE was defined as an enclosed room with virtual images projected onto the walls, floor, and ceiling. An HMD, in contrast, is a headset worn on the head, providing virtual images and sounds while shielding external stimuli. There was no further restriction regarding intervention content, for example, nature exposure, meditation task, or game. VR-based interventions with other aims than reducing PSL, such as stress inoculation training, pain management, physiological recovery, skill training, or the treatment of psychiatric disorders, were excluded. Furthermore, studies that used VR interventions for anxiety reduction during medical procedures, such as chemotherapy or dental surgery, were excluded to prevent potential biases caused by pain or medication effects. However, studies conducted in a clinical setting or among adults with a physical health condition (eg, cancer) were included if the intervention aimed at a reduction of PSL in general, independent of medical treatment, and no psychiatric diagnosis was involved. Further, inclusion required that the study design incorporated at least one neutral control condition or group, such as no intervention, treatment as usual, waitlist control, or a neutral environment (eg, urban or empty VR) as a placebo. To focus on the primary outcome of this review, only studies that provided PSLs, including arousal or negative affect, were considered. PSL had to be assessed using a validated questionnaire, a visual analog scale (VAS), or a numeric rating scale, with measurements taken at least postintervention. Secondary outcomes encompassed PSL in comparison to an active control group, as well as physiological stress parameters such as heart rate (HR), heart rate variability (HRV), skin conductance level (SCL), and systolic blood pressure (SYS). Secondary psychological outcomes included anxiety, depression, feelings of restoration, and positive emotions. Studies that were limited to physical stress outcomes were excluded.

### Study Selection

All records identified through the literature search were first screened for duplicates using the online tool Rayyan [41], followed by a manual verification by one reviewer (HS) to ensure accuracy. After the removal of duplicates, two researchers (HS and IS) independently screened the titles and abstracts to exclude clearly irrelevant studies. Any disagreements were resolved through discussion. Next, full texts of the remaining articles were retrieved and assessed for eligibility based on the above-mentioned predefined eligibility criteria. This process was conducted independently by two researchers (HS and IS). Disagreements were resolved through discussion, and if consensus could not be reached, a third reviewer (KM) was consulted. If relevant information was missing to clearly apply the eligibility criteria, the authors of the respective studies were contacted to provide further details.

### Data Collection

Relevant study information and data were extracted from eligible studies by 2 of 3 independent researchers (HS, IS, and KM) using a standardized spreadsheet form. To ensure accuracy, extracted data were compared and any discrepancies during the data extraction process were resolved through discussion. Extracted data items included (1) bibliographic information (first author, year, and country) and study design aspects (within-subjects or between-subjects, sample size, setting, and target population); (2) participant demographics, including age, gender, educational level, family status, race, health conditions, and prior VR experience; (3) numbers of participants in the intervention and control groups, as well as dropout numbers; (4) details of the VR intervention, such as conducted procedure, treatment duration, number and length of VR sessions, VR content and environment (provided relaxation technique, environment style and realism, content motion, technical aspects of the output device [device type, refresh rate, and field of view]), and level of user interactivity; (5) details of passive and, if applicable, active control conditions (procedure, treatment duration, intervention content); (6) details of measurements and analyses; and (7) reported outcome effects. The primary outcome variable of PSL, including negative affect or arousal, was addressed using a predefined hierarchical structure based on use frequency in the included studies to ensure standardized data extraction: Negative Affect Scale of Positive and Negative Affect Schedule (PANAS-NA), Profile of Mood States (POMS), State Scale of State-Trait Anxiety Inventory (STAI-S), Stress Scale of Depression, Anxiety, and Stress Scale (DASS-S), VAS for stress (VAS-S), Perceived Stress Scale (PSS), Warr’s Mood Scale (Warr’s) and miscellaneous measures. Secondary outcomes included physiological parameters of SCL, HR, HRV (high frequency component [HF-HRV], root mean square of successive differences [rMSSD], low frequency to high frequency ratio [LF/HF], and HRV response fraction), and SYS, as well as measures of restoration (Perceived Restorativeness Scale [PRS], Restoration Outcome Scale [ROS], and Recovery Experience Questionnaire [REQ]), positive emotion (positive affect scale of Positive and Negative Affect Schedule [PANAS-PA] and Zuckerman Inventory of Personal Reactions [ZIPERS]), anxiety (STAI-S, anxiety scale of Depression, Anxiety, and Stress Scale [DASS-A], anxiety subscale of Hospital Anxiety and Depression Scale [HADS-A], Generalized Anxiety Disorder [GAD] scale, VAS for anxiety [VAS-A], trait subscale of State-Trait Anxiety Inventory [STAI-T]), and depression (Self-Rating Depression Scale [SDS], depression subscale of Hospital Anxiety and Depression Scale [HADS-D], depression scale of Depression, Anxiety, and Stress Scale [DASS-D], depression subscale of Profile of Mood States [POMS-D], depression subscale of Warr’s Mood Scale [Warr’s-D], depression scale of Patient-Reported Outcomes Measurement Information System [PROMIS-D]). Additionally, measurements of perceived presence (IGroup Presence Questionnaire [IPQ], Presence Questionnaire [PQ], ITC-Sense of Presence Inventory [ITC-SOPI]), cybersickness (Simulator Cybersickness Questionnaire [SSQ]), and user experience with the VR intervention were considered. All effect measures were primarily extracted for postintervention time point in the form of mean and SD. If studies reported only standard errors, CIs, or IQRs, we calculated the SD according to methods described in the *Cochrane Handbook* [42]. In case of multisession interventions, the measure after the first session was extracted when reported. For between-subjects design studies that encompassed multiple relevant VR interventions (eg, an interactive VR game and a noninteractive VR exposure to nature), analyses were based on aggregated means and SDs across VR intervention groups to prevent double-counting of the neutral control. For within-subjects design studies, the VR intervention with the highest “dose,” such as the greatest level of biodiversity or stimulus intensity, was selected for inclusion. If relevant information on outcomes or intervention design was missing, the authors of the respective studies were contacted to obtain missing data. In case of nonresponse, studies with incomplete data on the PSL measure were excluded from the meta-analysis. Studies providing data on the primary outcome but limited information on intervention design or intervention characteristics were included in the main analyses. Further analyses only included studies providing the necessary information.

### Risk of Bias Assessment

The risk of bias (RoB) was assessed by two independent researchers (HS and KM) for each included study using the Cochrane Collaboration’s Risk of Bias tool for randomized trials [42]. Each study was evaluated as having a low, high, or unclear RoB across the following domains: random sequence generation (selection bias), allocation concealment (selection bias), blinding of participants and personnel (performance bias), blinding of outcome assessment (detection bias), incomplete outcome data (attrition bias), and selective reporting (reporting bias). Any discrepancies between reviewers were resolved through discussion. An overall RoB judgment was not conducted.

### Certainty of Evidence Assessment

The quality of evidence was assessed by two independent researchers (HS and KM) following the GRADE approach [38]. Therefore, the certainty of evidence was rated for each analyzed outcome (PSL, positive emotion, anxiety, depression, restoration, HR, HRV, SCL, and SYS) and categorized as high, moderate, low, or very low for the domains RoB, inconsistency, indirectness, imprecision, and publication bias. The overall certainty of evidence was determined by the GRADE classification system as high, moderate, low, or very low.

### Data Synthesis

To investigate potential predictors and differences in stress-reducing effects based on variations in VR interventions, studies were categorized for the domain setting, intervention type, environment realism, environment style, content motion, and user interactivity. Descriptions of the domains and the applied labels are provided in Multimedia Appendix 2. Ladakis et al [14] synthesize in a scoping review that most studies on VR interventions for stress reduction focus on the following application fields: the general population, students or working adults, groups with high daily stress, and people with special demands in a clinical setting. Therefore, labels for the domain setting were determined as general population, workplace/university, and clinical setting. Environmental realism was poorly described in most studies. Therefore, a binary labeling in real-world recording or computer simulation was conducted. As a dichotomous division (yes or no) did not adequately capture the variety of the included studies in content motion as well as in user interactivity, an intermediate stage was implemented. Consequently, there were studies with no (simple 3D photos or simulations without animated elements), low (a static scene from a consistent perspective with some moving events like leaves in the wind), or high (a moving scene with changing perspective like on a pathway) content motion. User interactivity was also divided into 3 levels, based on Zhang et al [43] and Gras [44]: low control (only watching and exploring scene by head movement), medium control (option to change perspective while moving in the environment by controller or body movement), or high control (option to interact with elements due to an interactive task or game). The assignment of the included studies to the different labels of domains is provided in the Results section. If a study evaluated the characteristics of multiple relevant VR interventions, the effect sizes were pooled and included as one intervention in subgroup analyses. If intervention arms differed in methodological or technical aspects and categorization was not clearly applicable for a domain, the study was excluded from analyses to prevent double counting.

### Statistical Analysis

The primary meta-analysis was conducted and visualized by forest plots using Cochrane's Review Manager (RevMan), version 9.11.0. As PSL was measured differently by the included studies, for each study, standardized mean differences (SMDs) between intervention and control groups were calculated for the primary outcome of PSL after the intervention, along with corresponding 95% CIs. Due to substantial variation in intervention design, a random-effects model following the restricted maximum likelihood method was applied to pool the results. As the approach described by Hartung, Knapp, Sidik, and Jonkman (HKSJ) is proven to perform better when trials of similar size are combined and to result in more adequate error rates, the HKSJ correction was applied to calculate CIs. Secondary outcomes were also analyzed using random-effects meta-analyses of SMDs. Following Cohen’s recommendations [45], SMDs can be categorized as none (SMD<0.2), small (0.2≤SMD<0.5), medium (0.50≤SMD<0.80), or large (SMD≥0.80) effect.

Statistical precision of the average intervention effect was assessed by *I*² statistic. Further, heterogeneity and the practical robustness of observed intervention effects were explored by 95% prediction intervals (PIs). As the PI for the primary outcome of PSL was not significant, sensitivity analyses were conducted to identify possible subgroups with significant PIs and thus more robust results. For this purpose, studies were subdivided based on study design, including gender ratio (men or women dominance), age group (<40 years or ≥40 years), setting (clinical setting or general population), target group, and intervention duration (single- or multisession intervention). Further subgroups were formed based on features of the VR intervention, including the used technical device (HMD or CAVE), intervention type, environment style and realism, content motion, and level of user interactivity. The presence of reporting bias was explored by examining funnel plot asymmetry and conducting the Egger test in IBM SPSS Statistics, version 30.0. To assess the robustness of the findings and to evaluate the potential influence of the study design, a series of sensitivity analyses were conducted.

Potential predictors of the effectiveness of VR-based interventions for stress reduction were examined through meta-regressions, conducted in IBM SPSS Statistics, version 30.0, using the inverse-variance method. As the number of studies providing all data for an overall multivariate regression model was inadequate for the number of observed predictors, predictors were purpose-guided and allocated to two subsets defined by study design and technical or content-related VR characteristics for analyses. The assignment of the separate predictors for different meta-regressions is provided in Table 2 as well as the total number of included studies and participants. Only studies providing information on all observed predictors in each subset were included in analyses. Subsequently, all ordinal and continuous explanatory variables regarding study design (mean age of participants, percentage of men, intervention duration in days, number of VR sessions, single session length) were separately tested in univariate linear regression analyses (32 studies). As the chi-square statistic (Multimedia Appendix 3) revealed that a higher age of participants and multisession interventions were more likely in the clinical setting, the setting was dummy coded and integrated in regression analyses (clinical setting=yes/no). Further, multivariate regression analyses examining technical and content-related VR characteristics were conducted in a subset of 17 studies that provided sufficient data. As statistical power was low, the number of investigated predictors had to be limited. The chi-square test revealed a high correlation of the intervention type with the level of content motion and user interactivity, wherefor this categorial variable was not considered. Meta-regressions were visualized using bubble plots, generated in R Studio (version 2026.01.00). For all statistical tests, a *P* value of <.05 was considered the threshold for statistical significance.

**Table 2.** Overview of the assignment of predictors to different meta-regression analyses to investigate possible predictors of the effectiveness of virtual reality interventions on perceived stress level in the general population, including the total number of included studies and participants for each analysis.

	Involved predictors	Included studies (references)	Studies, n	Participants, n
Study design	Mean age (years), males (%), multisession intervention (1,0), intervention duration (days), number of VR^a^ sessions (1-42), session length (min), clinical setting (1,0)	[46-77]	32	3023
Technical and content factors of VR	Content motion (1-3), user interactivity (1-3), environment realism (1,0), refresh rate (Hz), FoV^b^ (degree)	[46-48,50,51,53,55-57,60,64,66,68,69,76,78,79]	17	1831

a VR: virtual reality.

b FoV: field of view.

## Results

### Literature Search

**Figure 1.**
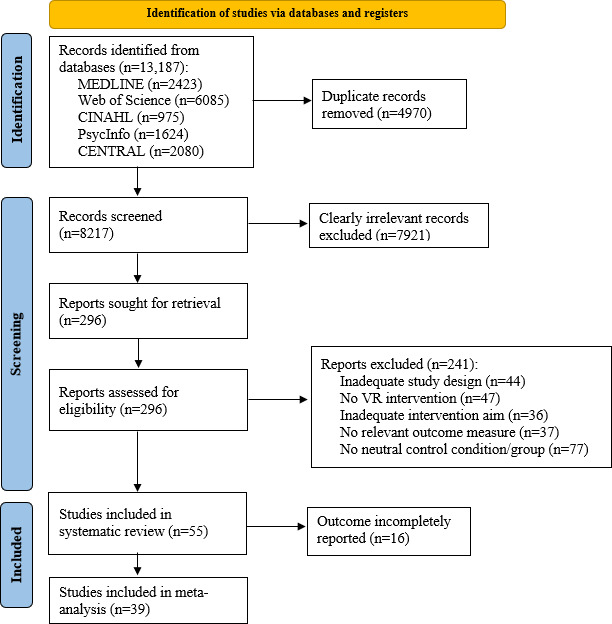
PRISMA (Preferred Reporting Items for Systematic Reviews and Meta-Analyses) flow diagram of the study selection process for the systematic review and meta-analysis on virtual reality interventions for stress reduction in the general population (searches conducted in June 2024 and February 2026). VR: virtual reality.

The PRISMA flow diagram (Figure 1) provides an overview of the study selection process. The database search yielded 13,187 results, of which 4971 were identified as duplicates and removed. Following the screening of the titles and abstracts of the remaining 8217 papers, 296 articles were retrieved and screened in full text. In total, 55 studies met the predefined and in methods section described inclusion criteria and were included in the systematic review. Of these, 16 publications did not provide complete data (ie, mean and SD postintervention for both intervention and control group) for the primary outcome of PSL and were excluded from the meta-analysis (Table 3, MA=“no”), resulting in 39 studies included in the quantitative synthesis. Studies that seemed to fit at first glance but were excluded during full-text screening are provided in Multimedia Appendix 4.

**Table 3.** Overview of the main characteristics of the included studies in the systematic review and meta-analysis on virtual reality interventions for stress reduction for the general population, providing details on study design, intervention setting, participants, intervention type and duration, and virtual reality output device.

Study ID	MA^a^	Country	Study design	Setting	Randomized, n	Men, %	Mean age, years	Intervention type	Control (active control)^b^	Output device	Duration of intervention	Session length (number)
Ahn 2025 [46]	Yes	Korea	Between	Workplace/university	41	26.8	21.3	Nature exposure	WLC (TSM)	HMD^c^	2 weeks	20 min (6)
Anderson 2017 [80]	No	USA	Within	General population	18	50.0	32.0	Nature exposure	eVR	HMD	1 time	15 min (1)
Bodet-Contentin 2023 [78]	Yes	France	Within	Workplace/university	88	19.3	—^d^	Meditation	TAU/NI	HMD	1 time	8 min (1)
Browning 2019 [47]	Yes	USA	Between	Workplace/university	98	43.9	20.2	Nature exposure	TAU/NI (RWE)	HMD	1 time	6 min (1)
Chand 2024 [48]	Yes	India	Between	Workplace/university	44	70.45	24.4	Miscellaneous	TAU/NI	HMD	6 days	15 min (6)
Chen 2024 [49]	Yes	USA	Within	General population	173	49.1	37.0	Nature exposure	bVR	CAVE^e^	1 time	5 min (1)
Da 2024 [81]	No	China	Between	General population	273	24.0	22.3	Biophilic design	bVR	HMD	1 time	3 min (1)
Emamjomeh 2020 [50]	Yes	USA	Within	Workplace/university	35	77.1	23.5	Biophilic design	eVR (RWE)	HMD	1 time	5 min (1)
Gao 2019 [82]	No	China	Between	Workplace/university	116	50.0	20.7	Nature exposure	bVR (aVR)	HMD	1 time	5 min (1)
Gao 2024 [83]	Yes	China	Between	Workplace/university	195	—	—	Nature exposure	eVR	HMD	1 time	3 min (1)
Gentile 2024 [51]	Yes	USA	Between	Workplace/university	409	39.1	19.4	Meditation	ot (TSM)	HMD	1 time	20 min (1)
Hessabi 2020 [52]	Yes	Iran	Between	Clinical setting	60	50.0	51.0	Nature exposure	TAU/NI	HMD	4 days	15 min (2)
Hooyberg 2023 [53]	Yes	Belgium	Within	General population	164	32.3	34.9	Nature exposure	bVR (aVR)	HMD	1 time	16 min (2)
Huang 2020 [54]	Yes	China	Between	Workplace/university	89	49.4	23.0	Biophilic design	bVR	HMD	1 time	10 min (1)
Hung 2025 [84]	No	Taiwan	Between	General population	111	50.0	24.0	Nature exposure	bVR	HMD	1 time	5 min (1)
Jiang 2025 [85]	No	China	Between	Workplace/university	66	—	26.9	Meditation	TAU/NI (TSM)	HMD	8 days	20 min (8)
Jimenez 2025 [86]	Yes	USA	Between	General population	200	19.0	—	Meditation	TAU/NI	HMD	1 time	6 min (1)
Jimenez-Barragan 2025 [55]	Yes	Spain	Between	Clinical setting	70	0.0	31.9	Meditation	TAU/NI	HMD	6 weeks	14 min (42)
Kawai 2024^f^ [56]	Yes	Switzerland	Between	General population	96	48.9	39.4	Nature exposure	bVR	HMD	1 time	20 min (1)
Kerr 2023 [57]	Yes	Swiss	Between	Workplace/university	87	51.7	22.9	Meditation	WLC	HMD	4 weeks	20‐26 min (4)
Kılıç 2023 [58]	Yes	Turkey	Between	Clinical setting	131	0.0	23.3	Nature exposure	TAU/NI	HMD	2 days	5 min (6)
Kim 2024 [59]	Yes	Korea	Between	Workplace/university	60	31.7	21.9	Meditation	TAU/NI (TSM)	HMD	5 days	30 min (5)
Knaust 2022 [87]	No	Germany	Within	General population	102	59.8	36.5	Nature exposure	TAU/NI (2D)	HMD	1 time	5 min (1)
Kosa 2024 [60]	Yes	USA	Between	Workplace/university	202	54.5	19.2	Game/activity	ot (2D, aVR)	HMD	1 time	15 min (1)
Kumpulainen 2024 [61]	Yes	Finland	Within	General population	57	33.3	40.0	Nature exposure	TAU/NI	CAVE	1 time	10 min (1)
Lepilkina 2023 [62]	Yes	Russia	Between	Clinical setting	68	44.1	42.9	Meditation	TAU/NI	HMD	5 days	20‐30 min (5)
Li 2025 [63]	Yes	China	Between	Clinical setting	98	39.8	60.5	Meditation	TAU/NI	HMD	1 time	25 min (1)
Liszio 2018 [64]	Yes	Germany	Between	General population	62	41.9	22.6	Nature exposure	TAU/NI (2D)	HMD	1 time	7 min (1)
Liszio 2019^f^ [65]	Yes	Germany	Between	General population	57	28.1	23.7	Game/activity, nature exposure	TAU/NI	HMD	1 time	9 min (1)
Liu 2023^f^ [66]	Yes	China	Within	Workplace/university	24	41.7	21.8	Biophilic design	bVR	HMD	1 time	6 min (1)
Ma 2025 [67]	Yes	China	Between	Workplace/university	170	33.5	19.9	Game/activity	TAU/NI (oVR)	HMD	1 time	25 min (1)
Ma 2026^f^ [68]	Yes	China	Between	General population	61	54.1	24.7	Biophilic design	bVR	HMD	1 time	6 min (1)
Mahmud 2022 [88]	No	Malaysia	Between	General population	67	37.3	33.2	Nature exposure	WLC (TSM)	HMD	2 weeks	5 min (6)
Martínez Manchón 2024^f^ [89]	Yes	Croatia	Between	Workplace/university	64	7.8	—	Game/activity, nature exposure	eVR	HMD	1 time	6 min (1)
Newman 2022^f^ [69]	Yes	England	Between	General population	120	13.3	20.0	Nature exposure	bVR	HMD	1 time	10 min (1)
Plante 2003 [90]	No	USA	Between	Workplace/university	154	33.8	—	Nature exposure	TAU/NI (RWE)	HMD	1 time	20 min (1)
Richesin 2021 [91]	No	USA	Between	Workplace/university	44	18.2	21.2	Game/activity	eVR (2D)	HMD	1 time	15 min (1)
Şansal 2024 [70]	Yes	Turkey	Within	General population	60	40.0	70.6	Nature exposure	bVR	HMD	1 time	6 min (1)
Schebella 2019 [92]	No	Australia	Between	General population	52	46.2	37.6	Nature exposure	bVR	HMD	1 time	5 min (1)
Schutte 2017 [71]	Yes	Australia	Between	Workplace/university	26	38.5	34.5	Nature exposure	bVR	HMD	1 time	6 min (1)
Song 2022 [72]	Yes	China	Between	Clinical setting	70	68.3	60.4	Nature exposure	bVR	HMD	1 week	5:20 min (3-5)
Sun 2023^f^ [73]	Yes	China	Between	Clinical setting	63	0.0	31.8	Nature exposure, biophilic design	bVR	HMD	1 time	5 min (1)
Suppakittpaisarn 2023 [79]	Yes	Taiwan, Thailand, USA	Between	General population	270	51.9	—	Nature exposure	bVR	HMD	1 time	1‐15 min (1)
Valtchanov 2010 [93]	Yes	Canada	Between	Workplace/university	69	46.4	—	Nature exposure	eVR (aVR)	HMD	1 time	10 min (1)
Villani 2008 [94]	No	Italy	Between	Workplace/university	64	46.9	24.5	Meditation	TAU/NI (2D, TSM)	HMD	1 week	— (2)
Wu 2025 [95]	No	China	Between	Clinical setting	85	0.0	46.4	Meditation	bVR	HMD	2 weeks	15 min (3-5)
Xiaoxue 2024 [96]	No	China	Within	General population	23	30.4	62.7	Biophilic design	bVR	HMD	1 week	5 min (1)
Yang 2024 [74]	Yes	Ukraine	Between	Workplace/university	120	60.8	22.0	—	TAU/NI	HMD	6 months	60 min (26)
Yildirim 2024 [75]	No	Australia	Within	Workplace/university	32	50.0	29.2	Biophilic design	bVR	HMD	1 time	6 min (1)
Yin 2024 [97]	No	China	Between	Workplace/university	320	43.8	21.1	Nature exposure	TAU/NI	HMD	1 time	3 min (1)
Yin 2020^f^ [76]	Yes	USA	Between	Workplace/university	100	37.0	29.0	Biophilic design	eVR	HMD	1 time	6 min (1)
Yu 2018 [98]	No	Taiwan	Within	Workplace/university	30	43.3	—	Nature exposure	bVR	HMD	1 time	9:30 min (1)
Yuan 2022 [99]	No	China	Between	Clinical setting	63	33.3	82.0	Nature exposure	TAU/NI	HMD	3 days	5 min (3)
Zhang 2022 [77]	Yes	China	Between	Clinical setting	90	0.0	51.7	Nature exposure	TAU/NI	HMD	3 months	30 min (6)
Zheng 2024^f^ [100]	Yes	China	Between	Workplace/university	60	26.7	20.2	Meditation, nature exposure	TAU/NI	HMD	2 weeks	10‐15 min (—)

a Study included (yes) or excluded (no) from meta-analyses (MA).

b Neutral control condition was categorized as “TAU/NI” (treatment as usual or no intervention), “eVR” (empty or blank VR), “bVR” (built or urban VR), “WLC” (waitlist control), or “ot” (other task). Active control condition was categorized as “2D” (2D simulation), “RWE” (real-world exposure), “TSM” (traditional stress management), or “aVR” (alternative VR intervention).

c HMD: head-mounted display.

d Not available.

e CAVE: cave automatic virtual environment.

f Study observed more than one VR intervention of interest (eg, noninteractive and interactive group, different levels of biodiversity/realism). Primary VR intervention and other VR intervention effects were pooled for analyses.

### Characteristics of Studies Included in the Systematic Review

Table 3 provides an overview of the main characteristics of the 55 studies included in this review [46-52,54-100]. Most studies were published within the past 5 years, while 8 were conducted between 2017 and 2019, and 3 prior to 2011. Among them, 43 studies used a between-subjects and 12 a within-subjects design. The 55 studies included one of the following neutral control conditions: 20 trials built/urban VR environment, 6 trials empty/blank VR control, 3 trials waitlist control, 23 trials treatment as usual/no intervention, and 2 trials other nonrelaxing tasks without VR. A total of 17 studies included an additional active control condition: 5 trials digital 2D simulations (2D), 3 trials real-world nature exposure (RWE), 6 trials traditional stress management (TSM) techniques, and 5 trials an alternative VR intervention that was not part of the main intervention focus. About 18 trials focused on the general population [49,53,56,61,64,65,68-70,79-81,84,86-88,92,96], 27 trials were conducted in work-related settings (2 in workplace, 23 in university, and 2 combined) [46-48,50,51,54,57,59,60,66,67,71,74-76,78,82,83,85,89-91,93,94,97,98,100], and 10 trials targeted subgroups with specific demands in clinical settings (eg, pregnant women, older adults, or clinical residents) [52,55,58,62,63,72,73,77,95,99].

Most studies (37 trials) implemented single-session interventions (Table 3). The remaining 18 studies involved multisession interventions lasting from 2 days to 6 months (1 trial 2 days, 1 trial 3 days, 1 trial 4 days, 2 trial 5 days, 1 trial 6 days, 3 trials 1 week, 1 trial 8 days, 3 trials 2 weeks, 1 trials 4 weeks, 1 trial 5 weeks, 1 trial 6 weeks, 1 trial 3 months, and 1 trial 6 months). The number of VR sessions ranged from 1 to 42 (37 trials 1 session, 9 trials 2‐5 sessions, 7 trials 6‐8 sessions, 2 trials more than 26 sessions). The duration of individual VR sessions ranged from 1 to 60 minutes (29 trials <10 min, 25 trials 10‐30 min, 1 trial>30 min).

A total of 31 studies investigated the effects of pure exposure to natural environments in VR (eg, forest, beach, or park) on stress reduction [46,47,49,52,53,56,58,61,64,65,70-73,77,79,80,82-84,87-90,92,93,97-100]. About 12 studies combined natural VR environments with a meditation task [51,55,57,59,62,63,78,85,86,94,95,100]. Another 9 studies studied the effect of biophilic design elements integrated into environments such as offices, streets, or courtyards in VR [50,54,66,68,73,75,76,81,96]. Five studies focused on games or interactive tasks as the main VR content [60,65,67,89,91], and 1 study [48] investigated miscellaneous content in VR. About 17 studies included more than one VR intervention to examine differences in factors such as environment type and biodiversity [49,53,54,66,68,69,72,73,76,80,82,92,96,97], meditation guidance [100], or level of user interactivity [65,89]. In nearly all studies (n=53), the VR intervention was delivered via HMD, while only 2 studies used a CAVE as an output device [49,61]. Around 28 studies used real-world recordings (photos or videos) as VR content [46-49,52,53,56,58,61,66,70-73,79,80,82-85,87,89,90,92,95,97-99], whereas 25 used a computer-generated simulation [50,51,54,55,57,59,60,62-65,67-69,75-78,81,86,88,93,94,96]. Two studies did not provide any technical details about the VR intervention.

### Meta-Analysis of Included Studies

#### Characteristics of Studies Included

A total of 39 studies provided sufficient data on the primary outcome of PSL and were included in the meta-analysis (Table 4) [46-53,55-79,83,86,89,93,100]. In total, the meta-analysis included 4024 participants, of whom 2179 received a VR-based intervention for stress reduction and 1845 were assigned to a neutral control condition. Participants’ mean ages ranged from 19.2 to 70.6 years (27 trials <40 years, 7 trials ≥40 years, 5 trials not reporting age). Further characteristics of the included studies are available in Table 3.

Possible predictors for stress reduction were analyzed based on both general study characteristics and further technical and content-related aspects of VR interventions. The detailed information used for categorization as well as reported outcome parameters for studies included in the meta-analysis are provided in Table 4. Most studies used a simple nature exposure intervention and offered limited user interaction, typically restricted to head movement for scene exploration.

**Table 4.** Technical details and reported outcome measures of the included randomized controlled trials in the quantitative synthesis of the systematic review and meta-analysis on virtual reality interventions for stress reduction in the general population.

Study ID	Session length, min (number)	Environment realism (style)^a^	Content motion^b^	FoV^c^, degree (refresh rate, Hz)	User interactivity^d^	Stress (PSL) outcome^e^	Anxiety / depression^f^	Positive emotion outcome^g^	Restorativeness outcome^h^	Physiological outcomes^i^
Ahn 2025 [46]	20 (6)	Recording (dl)	Low	134.38 (120)	Medium	PSS	PROMIS-A/PROMIS-D	—^j^	—	—
Bodet-Contentin 2023 [78]	8 (1)	Simulation (gs)	Low	126.57 (60)	Low	VAS-S	VAS-A/—	—	—	—
Browning 2019 [47]	6 (1)	Recording (gs)	Low	96.0 (60)	Low	PANAS-NA	—	PANAS-PA	PRS	—
Chand 2024 [48]	15 (6)	Recording (bs)	Low	134.38 (120)	Low	DASS-S	DASS-A/DASS-D	—	—	LF/HF
Chen 2024 [49]	5 (2)	Recording (gs)	Low	— (—)	Low	POMS	STAI-S/POMS-D	—	—	HR, HRV response fraction, SCL
Emamjomeh 2020 [50]	5 (1)	Simulation (ba)	No	138.59 (90)	Low	PANAS-NA	—	PANAS-PA	—	—
Gao 2024 [83]	3 (1)	Recording (gs)	—	150.57 (120)	Low	PANAS-NA	—/BPOMS-D	PANAS-PA	—	HR, SYS
Gentile 2024 [51]	20 (1)	Simulation (dl)	Low	145.17 (90)	Low	Other	—	Other	—	—
Hessabi 2020 [52]	15 (2)	Recording (—)	—	— (—)	—	STAI-S	—	—	—	—
Hooyberg 2023 [53]	16 (2)	Recording (bs)	Low	124.45 (80)	Low	PANAS-NA	—	PANAS-PA	—	HR, SCL
Huang 2020 [54]	10 (1)	Simulation (ba)	No	135.83 (75)	—	PANAS-NA	—	PANAS-PA	—	SCL
Jimenez 2025 [86]	6 (1)	Simulation (dl)	—	126.57 (60)	Low	STAI-S	VAS-A/—	—	—	—
Jimenez-Barragan 2025 [55]	14 (42)	Simulation (bs)	Low	126.57 (60)	Low	STAI-S	—	—	—	HR
Kawai 2024^k^ [56]	20 (1)	Recording (gs)	Low	134.38 (120)	Low	Other	—	—	ROS	SCL
Kerr 2023 [57]	21.5^l^ (4)	Simulation (gs)	Low	131.52 (75)	Low	DASS-S	DASS-A/DASS-D	—	—	HR, HF-HRV, SYS
Kılıç 2023 [58]	5 (6)	Recording (gs)	High	134.38 (120)	—	DASS-S	STAI-S/—	—	—	—
Kim 2024 [59]	30 (5)	Simulation (—)	—	134.38 (120)	Low	PSS	—	—	—	HRV
Kosa 2024 [60]	15 (1)	Simulation (gs)	High	134.38 (120)	High	PANAS-NA	STAI-S/—	PANAS-PA	REQ	—
Kumpulainen 2024 [61]	10 (1)	Recording (gs)	High	— (—)	Low	Warr’s	— /Warr’s-D	Other	—	HR, HF-HRV
Lepilkina 2023 [62]	25^l^ (5)	Simulation (dl)	—	124.45 (80)	—	STAI-S	—	—	—	—
Li 2025 [63]	25 (1)	Simulation (dl)	—	— (90)	Low	STAI-S	—	—	—	HR, SYS
Liszio 2018 [64]	7 (1)	Simulation (uw)	Low	123.75 (90)	Low	PANAS-NA	STAI-S/—	PANAS-PA	—	SDSD
Liszio 2019^k^ [65]	9 (1)	Simulation (bs)	Low	123.75 (90)	Low/high	PANAS-NA	STAI-S/—	PANAS-PA	—	SDSD
Liu 2023 [66]	6 (1)	Recording (ba)	High	138.59 (90)	Low	POMS	—	—	ROS	LF/HF
Ma 2025 [67]	25 (1)	Simulation (dl)	High	— (70)	High	PANAS-NA	STAI-S	PANAS-PA	—	HR
Ma 2026^k^ [68]	6 (1)	Simulation (bd)	High	138.59 (90)	Medium	STAI-S	—	—	—	HR, rMSSD, SCL
Martínez Manchón 2024^k^ [89]	6 (1)	Recording (dl)	Low/high	134.38 (120)	Low/high	VAS-S	—	—	—	—
Newman 2022 [69]	10 (1)	Simulation (gs)	High	145.17 (90)	Medium	PANAS-NA	—	PANAS-PA	—	—
Şansal 2024 [70]	6 (1)	Recording (gs)	Low	— (—)	Low	PANAS-NA	—	PANAS-PA	PRS	—
Schutte 2017 [71]	6 (1)	Recording (gs)	Low	— (—)	Low	PANAS-NA—		PANAS-PA	PRS	—
Song 2022 [72]	5.33^l^ (4)	Recording (dl)	No	130.11 (70)	—	PANAS-NA	—/SDS	PANAS-PA	—	HR, SYS
Sun 2023^k^ [73]	5 (1)	Recording (gs/ba)	Low	— (—)	Low	PANAS-NA	—	PANAS-PA	—	HR, SCL, SYS
Suppakittpaisarn 2023 [79]	7^l^ (1)	Recording (gs)	Low	131.52 (72)	Low	VAS-S	—	—	—	—
Valtchanov 2010 [93]	10 (1)	Simulation (bs)	High	— (—)	Medium	VAS-S	—	ZIPERS	—	HR
Yang 2024 [74]	60 (26)	—	—	145.17 (90)	Medium	STAI-S	STAI-T/—	—	—	—
Yildirim 2024 [75]	6 (1)	Simulation (ba)	—	134.38 (120)	—	Other	—	—	—	HR, SCL, SYS
Yin 2020 [76]	6 (1)	Simulation (ba)	No	145.17 (90)	Medium	STAI-S	—	—	—	HR, rMSSD, SCL, SYS
Zhang 2022 [77]	30 (6)	Simulation (bs)	High	— (—)	Medium	DT	SAS/SDS	—	—	—
Zheng 2024^k^ [100]	12.5^l^ (—)	— (dl)	—	— (—)	—	PSS	GAD/—	—	—	—

a Environment style was categorized as “gs” (green space, eg, garden, forest, park), “bs” (blue space, eg, island or beach), “dl” (different landscapes), “ba” (biophilic architecture, eg, office room, courtyard, street scenes), or “uw” (under water).

b Content motion was ranked as no motion (simple photos or simulations without animated elements), low (a static scene from a consistent perspective with some moving events like leaves in the wind), or high (a moving scene with changing perspective like on a pathway).

c Diagonal field of view (FoV) in degree: $\sqrt{{\left(horizontal\;\;fov\right)}^{2}+\left(vertical\;\;fov\right)2}$

d User interactivity was ranked as low control (only watching and exploring scenes by head movement), medium control (option to change perspective while moving in the environment by controller or body movement), or high control (option to interact with elements due to an interactive task or game).

e DASS-S: stress scale of Depression, Anxiety, and Stress Scale; DT: distress thermometer; PANAS-NA: negative affect scale of Positive and Negative Affect Scale; POMS: Profile of Mood States; PSL: Perceived Stress Level; PSS: Perceived Stress Scale; STAI-S: state scale of State-Trait Anxiety Inventory; VAS-S: Visual Analog Scale for Stress; Warr’s: Warr’s Mood Scale.

f BPOMS-D: depression subscale of Brief Profile of Mood States; DASS-A: anxiety scale of Depression, Anxiety, and Stress Scale; DASS-D: depression scale of Depression, Anxiety, and Stress Scale; GAD: Generalized Anxiety Disorder Scale; POMS-D: depression subscale of Profile of Mood States; PROMIS-A: anxiety scale of Patient-Reported Outcomes Measurement Information System; PROMIS-D: depression scale of Patient-Reported Outcomes Measurement Information System; SAS: Self-Rating Anxiety Scale; SDS: Self-Rating Depression Scale; STAI-T: trait subscale of State-Trait Anxiety Inventory; VAS-A: Visual Analog Scale for Anxiety; Warr’s-D: depression subscale of Warr’s Mood Scale.

g PANAS-PA: positive affect scale of Positive and Negative Affect Scale; ZIPERS: Zuckerman Inventory of Personal Reactions.

h PRS: Perceived Restorativeness Scale; REQ: Recovery Experience Questionnaire; ROS: Restoration Outcome Scale.

i HR: heart rate; HRV: heart rate variability; LF/HF: low frequency to high frequency ratio; rMSSD: root mean square of successive differences; SCL: skin conductance level; SDSD: standard deviation of successive differences; SYS: systolic blood pressure.

j Not available.

k Study observed more than one VR interventions of interest (eg, noninteractive and interactive group, different levels of biodiversity/realism). Primary VR intervention and other VR intervention effects were pooled for analyses. Subgroup analyses and meta-regression studies were excluded if categorization was not applicable.

l Study used different intervention durations or session lengths of VR without a separate data report. For analyses, the mean value was used.

#### Risk of Bias Assessment

**Figure 2.**
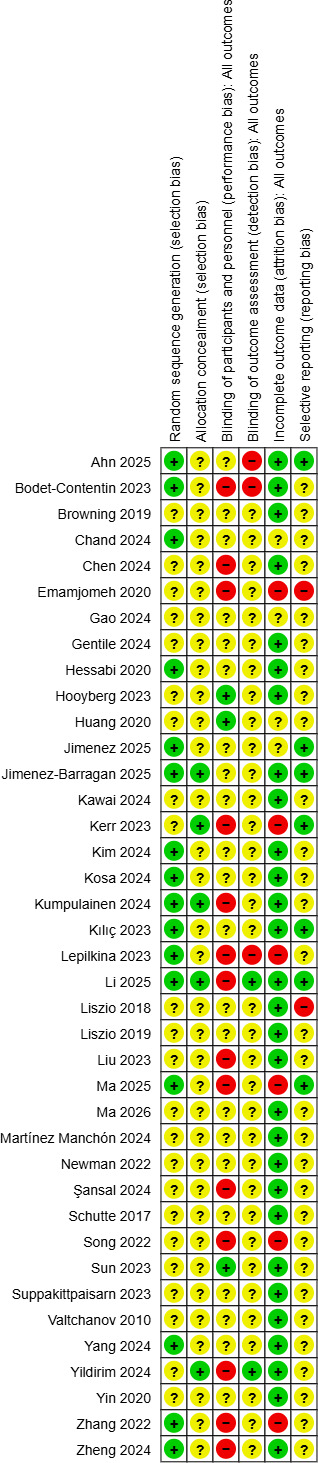
Risk of bias summary graph for the included studies in the meta-analysis on virtual reality interventions for stress reduction in the general population, generated using Cochrane's Review Manager (RevMan), version 9.11.0. Risk of bias was rated as low (green), high (red), or unclear (yellow) for each domain [46-74,76-79,81,83,86,89,93,100].

Summary graphs illustrating the RoB in the included studies are shown in Figure 2. The risk of selection bias was rated as low or unclear in all studies, with the latter primarily due to insufficient reporting on randomization and allocation procedures. Owing to the unblinded nature of the interventions, most studies were rated as having an unclear or high risk of performance and detection bias. Reporting bias was generally assessed as low for most of the studies.

#### Perceived Psychological Stress

**Figure 3.**
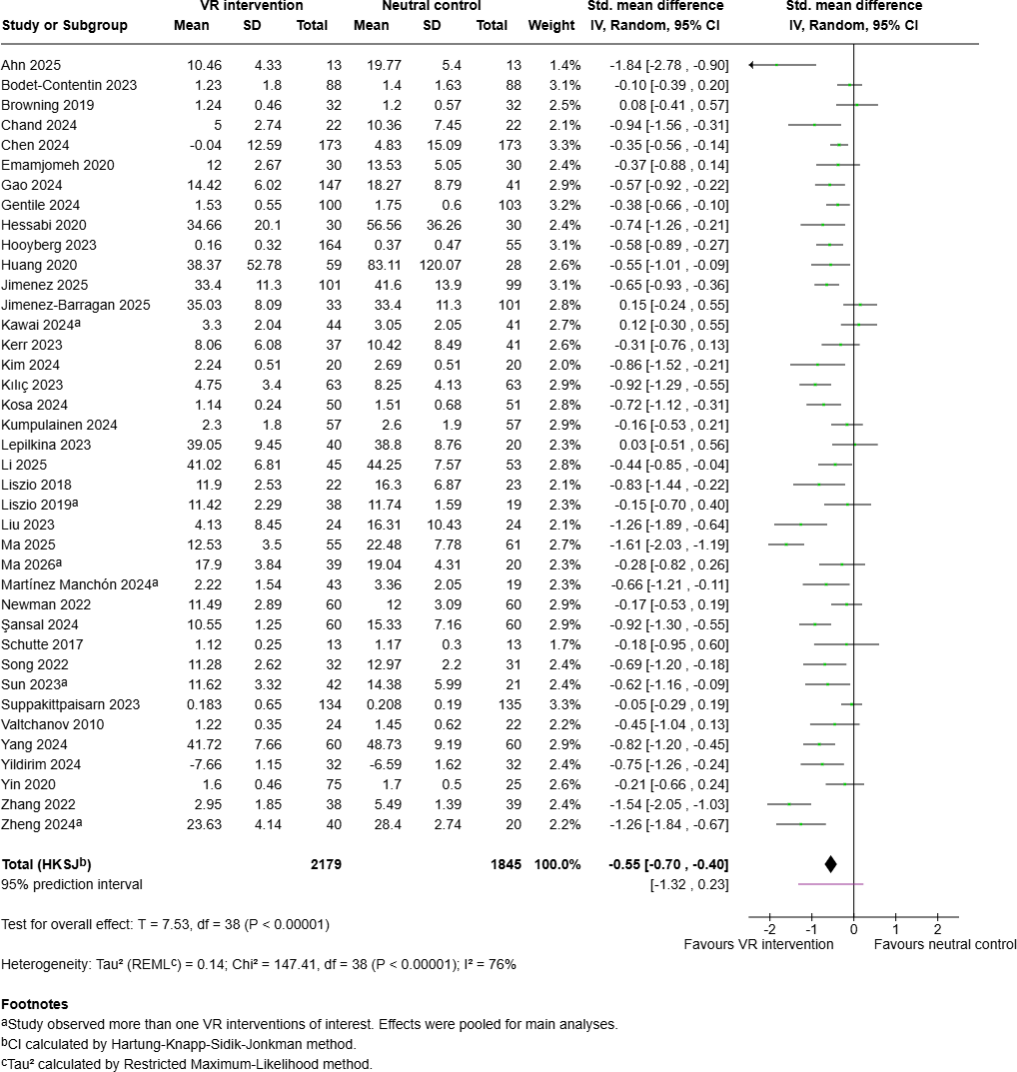
Forest plot for the primary outcome of perceived stress (postintervention) of the meta-analysis on virtual reality interventions for stress reduction in the general population, generated using Cochrane's Review Manager (RevMan), version 9.11.0, and including effect sizes for separate studies [46-74,76-79,81,83,86,89,93,100]. VR: virtual reality.

The forest plot for the postintervention effect on perceived stress is shown in Figure 3. On average, the pooled effect of VR-based interventions on PSL was significant (SMD −0.55, 95% CI −0.70 to −0.40; *P*<.001), considering the moderate-to-high level of variation in study outcomes (*I*²=76%, *P*<.001). However, the nonsignificant 95% PI (−1.32 to 0.23) suggests that while the majority of participants experienced a reduction in PSL through the VR intervention, a subset of participants showed no decrease, or even an increase in stress levels.

**Figure 4.**
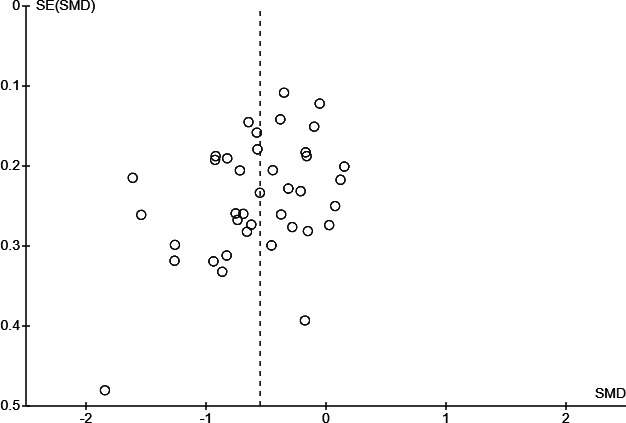
Funnel plot for the primary outcome of perceived stress (postintervention) of the meta-analysis on virtual reality interventions for stress reduction in the general population, generated using Cochrane's Review Manager (RevMan), version 9.11.0. SMD: standardized mean difference.

The Egger test for funnel plot asymmetry was marginally significant (intercept 0.66, *P*=.05). Moreover, inspection of the funnel plot (Figure 4) suggests the potential presence of small-study bias, possibly due to publication bias or the high heterogeneity of included studies.

To evaluate study quality as a potential source of heterogeneity, a series of sensitivity analyses were performed (Table 5). First, a sensitivity analysis only including between-subjects design studies was conducted to exclude possible carryover effects related to within-subjects design studies. To address potential biases from small sample sizes or a high RoB in critical domains, further analyses were restricted to studies with at least 100 participants, low risk of selection bias, and low risk of attrition bias. To examine the impact of potential performance bias, a further analysis only involved studies with low or unclear RoB. An additional sensitivity analysis excluded the two studies using a CAVE system as an output device and thus was limited to HMD. As illustrated in Table 5, all sensitivity analyses yielded significant pooled effect sizes (*P*<.001), while *I*² values remained at moderate to high levels. However, PIs did not reach significance in any of these analyses, indicating that the investigated factors did not contribute substantially to heterogeneity between studies.

To investigate variation in study outcomes and the precision of overall effects of VR-based interventions on PSL in more detail, further exploratory sensitivity analyses were performed. For this purpose, studies were stratified by age group (<40 years or ≥40 years), intervention duration (single-session or multisession), gender dominance (more women or more men), setting (general population or clinical), target population, and intervention type. In all subset analyses, *I*² values remained above 50%, and the range of the 95% PIs could not be reduced (see Multimedia Appendix 5) [46-74,76-79,81,83,86,89,93,100]. Thus, the sensitivity analyses did not explain the observed heterogeneity.

**Table 5.** Results of the sensitivity analyses for the primary outcome perceived stress level (postintervention) of the meta-analysis on virtual reality interventions for stress reduction in the general population, investigating effect sizes for randomized controlled trials exclusively with low risk of bias in critical domains, large sample size, or between-subjects study design.

	Random effects, SMD^a^ (95% CI)	*I*² value	95% PI^b^
All studies (n=39)	−0.55 (−0.70 to −0.40)	76%	−1.32 to 0.23
Low RoB^c^ – selection bias (n=16)	−0.74 (−1.04 to −0.43)	84%	−1.86 to 0.38
Low RoB – attrition bias (n=29)	−0.48 (−0.64 to −0.32)	72%	−1.17 to 0.21
Low or unclear RoB – performance bias (n=25)	−0.47 (−0.63 to −0.31)	64%	−1.07 to 0.13
Large sample size ≥100 (n=12)	−0.57 (−0.83 to −0.31)	83%	−1.41 to 0.27
Output device HMD^d^ (n=37)	−0.57 (−0.72 to −0.41)	76%	−1.36 to 0.23
Between-subjects design (n=31)	−0.56 (−0.73 to −0.28)	77%	−1.40 to 0.28

a SMD: standardized mean difference.

b PI: prediction interval.

c RoB: risk of bias.

d HMD: head-mounted display.

#### Objective Stress Measures

A total of 18 studies reported at least one physiological stress parameter (12 trials HR, 10 trials HRV, 8 trials SCL, and 7 trials SYS). Most objective stress outcomes showed no significant intervention effect compared with the neutral control group: HR (SMD −0.08, 95% CI −0.21 to 0.05, *P*=.22; 95% PI −0.26 to 0.11); HRV (SMD 0.23, 95% CI −0.09 to 0.54, *P*=.14; 95% PI −0.59 to 1.04); and SCL (SMD −0.03, 95% CI −0.15 to 0.10, *P*=.60; 95% PI −0.15 to 0.10). A significant intervention effect was observed for SYS (SMD −0.20, 95% CI −0.37 to −0.04, *P*=.02; 95% PI −0.37 to −0.04). Forest plots for the physiological outcomes are provided in Multimedia Appendix 6 [48,49,53-57,59,61,63-68,72,73,75,76,83,93], including RoB assessment.

#### Secondary Psychological Outcomes

A total of 17 studies reported a measure of positive emotion. To ensure a consistent direction of effects across outcomes, effect sizes were coded such that negative SMDs indicate beneficial effects of VR interventions. The pooled SMD showed a significant effect of VR interventions on positive emotion (−0.65, 95% CI −0.84 to −0.46, *P*<.001), with a moderate level of heterogeneity (*I*²=62%, *P*<.001). As the 95% PI (−1.28 to −0.02) reached significance, VR interventions appear to consistently promote positive emotions across participants. Measures of anxiety and depression were reported in 14 and 8 studies, respectively. On average, significant pooled effects were observed for both outcomes: anxiety (SMD −0.88, 95% CI −1.23 to −0.54, *P*<.001; 95% PI −2.07 to 0.31) and depression (SMD −0.34, 95% CI −0.47 to −0.21, *P*<.001; 95% PI −0.47 to −0.21). However, the heterogeneity was high for anxiety (*I*²=87%, *P*<.001) and negligible for depression (*I*²=0%, *P*=.71). The nonsignificant PI for anxiety suggests that VR interventions did not reduce, or even increase, anxiety in some participants, whereas depression symptoms decreased more consistently. Additionally, 6 studies reported a measure of restoration, for which the pooled average effect was significant (SMD −1.50, 95% CI −2.58 to −0.42, *P*=.02). Again, the nonsignificant 95% PI (95% PI −4.26 to 1.26) indicates even negative effects on restoration for some individuals. Forest plots and effect sizes of each study are provided in Multimedia Appendix 6, including RoB assessment.

#### Effects of VR-Based Interventions Compared to Active Controls

**Figure 5.**
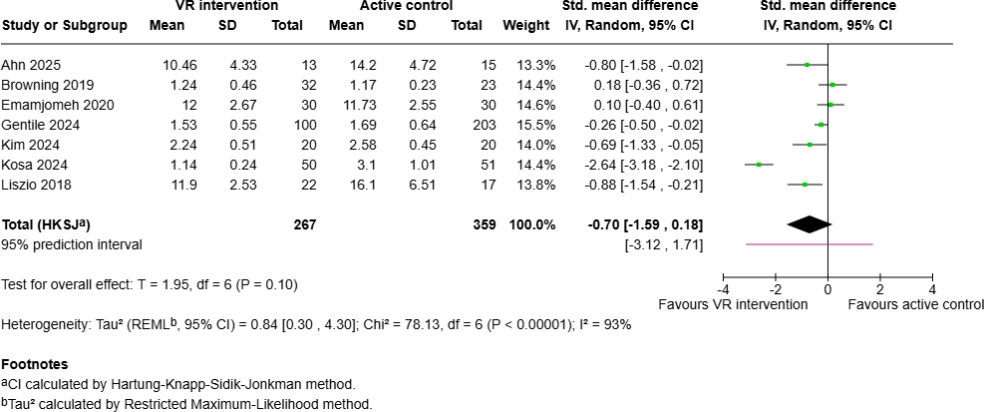
Forest plot of the meta-analysis on virtual reality interventions for stress reduction in the general population for the primary outcome PSL (postintervention) in comparison to active control condition, generated using Cochrane's Review Manager (RevMan), version 9.11.0 [46,47,50,51,59,60,64]. PSL: perceived stress level; VR: virtual reality.

A total of 7 studies included an active control condition without a VR component (2 trials RWE, 3 trials 2D, 2 trials TSM). The pooled SMD for PSL showed no significant effect of VR interventions in comparison to active controls (SMD −0.70, 95% CI −1.59 to 0.18, *P*=.10; 95% PI −3.12 to 1.71; Figure 5). Five studies also provided data on positive emotion, for which no significant effect of VR interventions was observed when compared to active controls (SMD −0.03, 95% CI −0.80 to 0.73, *P*=.91; 95% PI −1.70 to 1.64).

#### Certainty of Evidence

We identified moderate-quality evidence that VR interventions enhance positive emotion in the general population compared with neutral control conditions (Table 6). The certainty of evidence for PSL and most other psychological outcomes was rated as low to very low. In contrast, all physiological stress parameters showed a moderate or high certainty of evidence.

**Table 6.** Summary of findings of the meta-analysis on virtual reality interventions for stress reduction in the general population according to GRADE^a^, including certainty of evidence assessment^b^.

Outcome	SMD^c^ (95% CI)	Participants (studies)	Certainty of the evidence (GRADE)^d^	Comments
Perceived stress level	−0.55 (−0.70 to −0.40)	4024 (39 RCTs)^e^	⨁◯◯◯ Very low	In 4 studies, PSL^f^ slightly increased on a nonsignificant level, while 35 demonstrated decreased PSL. Nonsignificant PI^g^ suggested variety in intervention effects.
Positive emotion	−0.65 (−0.84 to −0.46)	1688 (17 RCTs)	⨁⨁⨁◯ Moderate	Positive emotion was enhanced consistently in all studies, supported by the significant PI.
Anxiety	−0.88 (−1.23 to −0.54)	1472 (14 RCTs)	⨁◯◯◯ Very low	All studies showed an average decrease in anxiety.
Depression	−0.34 (−0.47 to −0.21)	936 (8 RCTs)	⨁⨁◯◯ Low	All studies showed a consistent decrease in depression, supported by the significant PI.
Restoration	−1.50 (−2.58 to −0.42)	440 (6 RCTs)	⨁◯◯◯ Very low	—^h^
Heart rate	−0.08 (−0.21 to 0.05)	1398 (12 RCTs)	⨁⨁⨁◯ Moderate	—
Heart rate variability	0.23 (−0.09 to 0.54)	901 (10 RCTs)	⨁⨁⨁◯ Moderate	—
Systolic blood pressure	−0.20 (−0.37 to −0.04)	654 (7 RCTs)	⨁⨁⨁⨁ High	6 of 7 studies showed a decrease in SYS^i^. Consistent effects on SYS are underlined by the significant PI.
Skin conductance level	−0.03 (−0.15 to 0.10)	992 (8 RCTs)	⨁⨁⨁◯ Moderate	—

a GRADE: Grading of Recommendations Assessment, Development, and Evaluation.

b People: adults without psychiatric diagnoses; Settings: general population; Intervention: immersive virtual reality interventions for stress reduction; Comparison: neutral control.

c SMD: standardized mean difference.

d *High*: This research provides a very good indication of the likely effect. The likelihood that the effect will be *substantially different* is low. *Moderate*: This research provides a good indication of the likely effect. The likelihood that the effect will be *substantially different* is moderate. *Low*: This research provides some indication of the likely effect. However, the likelihood that it will be *substantially different* is high. *Very low*: This research does not provide a reliable indication of the likely effect. The likelihood that the effect will be *substantially different* is very high. *Substantially different* means a large enough difference that it might affect a decision.

e RCT: randomized controlled trial.

f PSL: perceived stress level.

g PI: prediction interval.

h Not applicable.

i SYS: systolic blood pressure.

#### Predictors for the Effectiveness of VR-Based Interventions for Psychological Stress Reduction

To identify potential explanatory variables for the stress-reducing effects of VR interventions, several regression analyses were conducted.

Given that only 15 studies provided complete data for all predictors, an overall multivariate regression incorporating all 12 predictors was not feasible due to insufficient statistical power. Consequently, explanatory variables were grouped into study design aspects and technical or content-related characteristics of the VR intervention for the regression analyses. Potential predictors included the percentage of men, mean age of study participants, intervention duration in days, number of VR sessions, length of single VR session, setting, refresh rate, diagonal field of view, level of content motion, environment realism, level of user interactivity, and intervention type (for details, see Table 2).

Results of the univariate linear regression analyses of study-design related factors as potential predictors for the intervention effects are summarized in Table 7. Including 32 studies with a total of 3023 participants (SMD −0.56, 95% CI −0.73 to −0.39, *P*<.001; 95% PI −1.38 to 0.26; *I*²=76%), no significant effects were found for age (*P*=.36) or gender (*P*=.32). Multisession interventions predicted larger stress-reducing effects (*R*²=15.0%, *F*_1,30_=6.66; *P*=.02) compared with single-session approaches. No significant associations were identified for intervention duration in days (*P*=.26), number of VR sessions (*P*=.91), or length of individual sessions (*P*=.09). Multivariate regression analyses (Table S1 in Multimedia Appendix 7) did not identify any significant predictors, which may be attributable to interdependencies among single factors.

**Table 7.** Univariate linear regression analyses for possible predictors of the effectiveness of virtual reality (VR) interventions based on participants and intervention design (n=32) in the framework of the meta-analysis on VR interventions for stress reduction in the general population^a^^,b^.

Predictor	Estimate	SE	2-tailed *t* test (*df*)	*P* value	*F* test (*df*)	*τ*²	*R*²
Mean age (years)	0.01	0.01	0.94 (30)	.36	0.88 (1, 30)	0.005	0%
Males (%)	0.01	0.01	1.02 (30)	.32	1.04 (1, 30)	0.005	0%
Clinical setting (1,0)^c^	−0.12	0.25	−0.48 (30)	.64	0.23 (1, 30)	0.005	0%
Intervention duration (days)	−0.00	0.00	−1.15 (30)	.26	1.33 (1, 30)	0.005	1.1%
Multisession intervention (1,0)^d^	−0.50	0.19	−2.58 (30)	.02	6.66 (1, 30)	0.004	15%
Number of VR sessions	−0.00	0.02	−0.12 (30)	.91	0.01 (1, 30)	0.005	0%
Single VR session length (min)	−0.02	0.01	−1.75 (30)	.09	3.05 (1, 30)	0.005	6%

a Analyses were conducted in IBM SPSS Statistics, version 30.0, using a random-effects model with HKSJ (Hartung, Knapp, Sidik, and Jonkman) correction.

b Effect sizes of different VR interventions of one publication were pooled for the main analyses and are included as one intervention group [56,65,68,73].

c Clinical setting was dummy coded as “1=clinical setting” and “0=nonclinical setting.”

d Intervention duration was dummy coded as “1=multisession intervention” and “0=single-session intervention.”

We therefore performed explorative chi-square tests (Tables S1 and S3 in Multimedia Appendix 3), which revealed codependencies between the predictors age, setting, and intervention duration. Specifically, clinical settings were more likely to involve multisession interventions (*χ*²_2_=14.49, *P*<.001) and to include older participants (*χ*²_2_=9.41, *P*=.009).

Next, the explanatory role of content-related and technical characteristics of the VR intervention (eg, content motion, user interactivity, environment realism, field of view, and refresh rate) on the stress-reducing effects of VR interventions was investigated in linear regression analyzes based on a subset of 17 studies with a total of 1831 participants (SMD −0.37, 95% CI −0.61 to −0.14, *P*=.004; *I*²=73%; 95% PI −1.13 to 0.38). Univariate regression analyses (Table 8) identified significant results for image refresh rate (*R*²=42.1%, *F*_1,15_=12.91, *P*=.003) as well as real-world recordings (*R*²=23.8%, *F*_1,15_=6.17, *P*=.03) in comparison to computer-simulated content. The multivariate model (Table S2 in Multimedia Appendix 7) showed significance and demonstrated a predictive power of 42.9% (*R*²=42.9%, *F*_5,11_=3.43, *P*=.04). Within this model, a higher image refresh rate (*t*_15_=−2.36, 2-tailed; *P*=.04) was identified as a potential independent predictor of the intervention effects on PSL. Environment realism (*P*=.43) did not remain a significant predictor, which may be attributable to interdependencies between the environment realism and intervention type, showing real-world recordings more likely in nature exposure interventions in comparison to other types (*χ*²_3_=17.09, *P*<.001) with more passive levels of user interactivity (Tables S2 and S4 in Multimedia Appendix 3).

**Table 8.** Univariate linear regression analyses for possible predictors of the intervention effectiveness based on content-related and technical aspects (n=17), performed in the framework of the meta-analysis on virtual reality interventions for stress reduction in the general population^a^.

Predictor	Estimate	SE	2-tailed *t* test (*df*)	*P* value	*F* test (*df*)	*τ*²	*R*²
Content motion^b^	−0.13	0.30	−0.42 (15)	.68	0.18 (1, 15)	0.01	0%
User interactivity^c^	−0.15	0.27	−0.55 (15)	.59	0.30 (1, 15)	0.01	0%
Environment realism^d^	−0.73	0.29	−2.49 (15)	.03	6.17 (1, 15)	0.01	23.8%
Refresh rate (Hz)	−0.02	0.01	−3.59 (15)	.003	12.91 (1, 15)	0.01	42.1%
FoV^e^ (degree)	−0.02	0.02	−0.94 (15)	.36	0.88 (1, 15)	0.01	0%

a Analyses were conducted in IBM SPSS Statistics, version 30.0, using a random-effects model with HKSJ (Hartung, Knapp, Sidik, and Jonkman) correction.

b Content motion was categorized as “1=no motion” (simple 3D images without moving elements), “2=low motion” (static scene with some moving elements), or “3=high motion” (dynamic scene with changing perspective).

c User interactivity was ranked as “1=low control” (only watching and exploring scene by head movement), “2=medium control” (option to change perspective while moving in environment by controller or body movement), or “3=high control” (option to interact with elements due to an interactive task or game).

d Environment realism was dummy coded as dichotomic variable “real-world recording” (1=yes, 0=no) for regression analyses.

e Diagonal field of view (FoV) was calculated by the following formula: $\sqrt{{\left(horizontal\;\;fov\right)}^{2}+\left(vertical\;\;fov\right)2}$

## Discussion

### Summary of Findings

This systematic review synthesizes evidence on the effectiveness of various VR-based interventions for stress reduction across a broad range of application areas and identifies characteristics that may predict this effectiveness. The meta-analyses demonstrated that VR-based interventions led to significantly greater reductions in PSL, anxiety, and depression as well as increases in positive affect, compared to neutral control conditions. However, the nonsignificant PIs for reductions in negative affect pointed to substantial heterogeneity, supporting the notion that VR interventions do not improve psychological stress in all individuals similarly. In contrast, significant PIs for positive affect and depressive symptoms suggest more consistent beneficial effects in these domains. Regarding physiological stress markers, no significant effects were observed for most parameters (eg, HR, HRV, or SCL). Nevertheless, a significant reduction was found for SYS, with significant PIs indicating a robust and consistent intervention effect. Beyond establishing the overall effectiveness of VR interventions in reducing PSL, this meta-analysis also identified several potential predictors of enhanced intervention outcomes. Specifically, multisession interventions, higher image refresh rates, and increased environmental realism, particularly through real-world recordings, were positively associated with stronger stress-reducing effects of VR interventions in univariate regression analyses.

Most of the included studies were published within the past 5 years, reflecting the rapidly growing interest in the use of VR for managing psychological stress. Among the various intervention types identified, virtual exposure to natural environments emerged as the most frequently investigated approach. These interventions were predominantly passive in nature, offering limited interactivity, although some incorporated additional elements such as guided meditation or interactive mini-games to enhance their stress-reducing effects. The majority of studies used HMDs as a delivery method for VR content, were conducted in university or workplace settings, and evaluated single-session interventions. In contrast, RCTs evaluating long-term interventions or targeting specific populations with specific needs, such as pregnant women or older adults, were less common and warrant further investigation. Overall, the findings of this systematic review highlight the considerable diversity of VR-based interventions for stress reduction, characterized by substantial heterogeneity in target populations, methodological and technical design, as well as in reported levels of immersion, presence, and user experience.

### Interpretation and Implications of Findings

While previous qualitative literature reviews highlighted the general effectiveness of VR interventions for stress reduction [14,24,26-28,35], the present review extends those findings by quantifying intervention effects for stress reduction and statistically identifying possible predictors of intervention success. Chen et al [34] noted that existing meta-analyses have primarily focused on clinical populations, which motivated their investigation of virtual nature exposure and 2D images on anxiety, stress, and depression in healthy adults. In contrast, our analysis included a broader range of VR-based approaches, especially those delivered through immersive technologies. The meta-analysis of 39 RCTs confirmed that VR interventions can effectively reduce psychological stress in the general population. However, the wide and nonsignificant PIs indicate substantial variability in intervention effects and suggest that not all subgroups may benefit equally in practice. One possible explanation for this heterogeneity is the occurrence of cybersickness [101], which has been associated with increases in physiological stress markers (eg, cortisol levels and HR) [102], elevated psychological stress [103], and diminished intervention effects (eg, on anxiety) [104]. As cybersickness is likely influenced by characteristics of the VR content [105], it may contribute, together with the heterogeneity of intervention types, to the observed variability in PSL reduction. However, as only 4 of the included studies assessed cybersickness, its impact on intervention outcomes could not be systematically evaluated.

Univariate regression analyses further indicated that multisession interventions were associated with greater effectiveness compared to single-session interventions. This finding is consistent with previous research suggesting that longer-term interventions may be more beneficial for stress reduction [106]. One possible explanation is that studies using multisession designs more frequently targeted populations with elevated baseline stress levels, such as clinical samples [52,55,58,62,72,77] or individuals in workplace and university settings [46,48,57,59,74,100]. In contrast, single-session studies often involved experimentally induced, transient stress in younger participants [56,64,69,79,83,93]. This distinction is relevant, as existing evidence indicates that individuals with chronic conditions [107,108] and university students [109-111] are particularly susceptible to higher and more persistent stress levels. Moreover, while chronic stress typically requires repeated intervention exposure, acute stress may respond to brief relaxation techniques [112,113]. Accordingly, the optimal number, duration, and frequency of VR sessions likely depend on the nature of stress (chronic vs acute) and remain to be clarified by future research.

Notably, age and intervention setting were not identified as significant predictors in our regression analyses. Similarly, Li et al [29] reported no significant influence of age or session duration on the effectiveness of VR-based nature exposure on positive affect, while highlighting an overrepresentation of younger adults in the existing literature. Nevertheless, other studies suggest that older adults may represent a particularly promising target group for VR-based stress reduction [114], potentially benefiting even more than college students [115].

We also examined methodological and technical characteristics of VR content as potential predictors of VR intervention success. Our analyses indicated that the type of environment (eg, blue vs green space) did not significantly influence stress-reducing effects of VR interventions, consistent with the findings of the narrative review by Li et al [116]. In contrast, univariate regression analyses identified environmental realism (eg, computer simulation vs real-world video recordings) as a significant predictor, with stronger stress reduction observed for real-world recordings. However, this effect did not persist in multivariate analyses, possibly due to interdependencies with other factors such as intervention type and user interactivity. Although user interactivity did not emerge as a significant predictor, the observed effect size suggests that a more passive user experience may be associated with greater reductions in PSL. This is in line with findings by Reese et al [117], who reported stronger stress-reducing effects for passive VR nature exposure compared to active engagement within the VR environment. At the same time, the high heterogeneity of effects in studies involving active tasks, along with the limited representation of highly interactive interventions in our dataset, may have reduced statistical power and contributed to the lack of significance [118]. Moreover, previous research indicates that higher interactivity may enhance positive affect [97], suggesting that its impact may differ depending on the outcome domain (eg, negative vs positive emotions) as well as individual user preferences. Accordingly, the role of user interactivity and environmental realism warrants further investigation. The level of content motion was not identified as a significant predictor in our analyses, although individual studies have reported beneficial effects of dynamic content on physiological outcomes such as heart rate [119], as well as higher perceived presence for video-based compared to slideshow-based VR environments [120,121]. Moreover, this meta-analysis identified refresh rate as a promising predictor of stress-reducing effects of VR. However, the refresh rate considered in this meta-analysis reflects only the technically available frequency of image updates of the used HMD, whereas the actual rate at which images appear on the display (framerate) depends on the rendering of raw recordings into the VR content [122], for which insufficient information was available. As a higher refresh rate is generally associated with a better user experience, which is closely linked to interactivity and content motion [95,98,99], the observed interrelations between these variables are to be expected. It is therefore plausible that a smoother and more coherent user experience plays a crucial role for the effectiveness of VR-based stress interventions. Overall, given the substantial heterogeneity across studies, further research is needed to better understand predictors of intervention effects, particularly with regard to potential differences between chronic stress and acute mental load. Although the multivariate model showed significant predictive power, it did not fully account for the variability in outcomes, indicating that additional, as yet unidentified factors are likely to influence intervention success.

Significant effects of VR interventions were also observed for secondary outcomes, particularly increased positive affect and reductions in anxiety and depressive symptoms. Notably, the PIs for positive emotion and depression were significant, suggesting that these beneficial effects are relatively consistent across individuals. In contrast, nonsignificant PIs for other negative emotional outcomes (eg, PSL or anxiety) indicate that improvements in these domains may depend on additional factors. VR interventions were further associated with a significant reduction in SYS, with significant PIs indicating consistent effects across participants. These findings are in line with previous research, such as Yao et al [13], which demonstrated stress-reducing effects of VR interventions on both psychological and physiological parameters. Moreover, the beneficial impact of (virtual) nature exposure on blood pressure has been summarized in prior reviews [123,124]. While Song et al [124] also reported positive effects on HR and HRV, no significant effects on these or other physiological stress markers were observed in our analyses. Several factors may explain this discrepancy. First, the number of studies reporting physiological outcomes was relatively small, potentially limiting statistical power. Second, multisession interventions, which tend to yield stronger effects, were underrepresented in the available data. Third, substantial variability in the assessment and reporting of physiological measures, as well as potential limitations in measurement sensitivity, may have obscured detectable effects. The considerable heterogeneity in physiological outcomes is also reflected in previous studies [125,126], which attribute inconsistencies to differences in study design and population characteristics [126]. Overall, our findings are consistent with prior meta-analyses reporting reductions in PSL and increases in positive affect following VR interventions, alongside nonsignificant effects on cardiovascular parameters such as HR and HRV when compared to neutral control conditions [29,115].

Consistent with our finding of no substantial differences between VR interventions and active control conditions, such as 2D simulations or real-world nature exposure, previous studies suggest that VR-based nature exposure may be as effective as real-life exposure. For instance, Reese et al [127] reported similar improvements in well-being and PSL following both digital forest bathing and in vivo nature exposure. Likewise, the meta-analysis by Fan and Baharum [128] confirmed VR-based nature exposure as a viable and nearly equivalent alternative to real-world nature experience for stress reduction.

### Limitations

Several limitations should be considered when interpreting the results of this meta-analysis. First, no information scientist was involved in developing the search strategy, and relevant studies may therefore have been missed. However, the search followed a systematic and transparent strategy based on the Cochrane guidelines and the PICO framework, and the final search string was pretested and refined according to the PRESS guidelines by 2 independent researchers. Second, due to the nature of VR intervention studies, blinding of group allocation is rarely feasible, and only 3 studies implemented blinded control conditions. As a result, potential biases related to placebo effects or response bias cannot be fully excluded. However, evidence from VR-based pain management suggests that expectation effects may be limited in this context [129]. Third, many included studies exhibited a high risk of selection, performance, and detection bias, highlighting methodological limitations within the existing literature. Fourth, although strict inclusion criteria were applied to reduce heterogeneity, considerable variability between studies remained. Analyses could not identify more homogeneous subsets of studies with more robust effects regarding stress reduction through VR interventions. Prediction intervals were often nonsignificant, and *I*² values ranged from moderate to high, limiting the generalizability of the findings across populations. Fifth, the chi-square test indicated associations between several predictors, suggesting potential confounding. For example, multisession interventions were predominantly conducted in clinical settings and involved participants over 40 years of age. These interdependencies should be taken into account when interpreting the predictive value of related factors such as intervention duration in univariate analyses. Sixth, interventions involving active user engagement, such as interactive tasks or the use of controllers, remain underrepresented in the current literature, with the majority of interventions offering a passive VR experience [14]. Consequently, the stress-reducing potential of more interactive VR interventions cannot yet be adequately assessed. Seventh, limited reporting of participant characteristics (eg, prior VR experience) and insufficient detail on intervention features restricted the feasibility of conducting more robust subgroup and regression analyses. As a result, the statistical power of predictor analyses was limited and should be considered when interpreting these findings. Eighth, data on potential predictors were inconsistently reported, hampering a more comprehensive examination of their influence. It is therefore possible that additional unmeasured factors contribute to intervention effectiveness, which may explain the relatively low proportion of explained variance observed in the analyses.

### Conclusions

While previous reviews have demonstrated the general effectiveness of VR interventions for stress reduction without quantifying their effects [14,24,26-28,35], or have focused on specific subgroups [32,130,131] or intervention types [29,30,128], this review highlights the considerable potential of VR-based interventions to reduce psychological stress in the general population. By quantifying effects across a broad range of approaches and, for the first time, directly comparing diverse intervention formats, this meta-analysis provides a more comprehensive understanding of their effectiveness. Given that VR-based interventions appear comparable in effectiveness to established stress management techniques, they represent a promising alternative, particularly in contexts where access to real-life nature or conventional interventions is limited or resource-intensive. While the findings suggest reliable improvements in positive affect and reductions in depressive symptoms, effects on PSLs were more variable, with some individuals experiencing limited benefit. This variability was observed in nearly all subgroups, suggesting that individual characteristics and contextual factors play a critical role in shaping intervention outcomes. Multisession interventions generally yielded stronger effects than single-session designs, whereas intervention duration and total number of VR sessions did not emerge as significant predictors. Thus, the optimal number, duration, and frequency of VR sessions remain unclear and may differ depending on whether stress is acute or chronic. Future studies with larger and more diverse samples are needed to directly compare effects across target groups and application contexts, including short-term stress induction and everyday stress experiences.

This meta-analysis further identified characteristics associated with a more calming user experience, such as higher refresh rates and lower levels of user interactivity, as potential predictors of stronger stress-reducing effects. These findings may inform the design of future VR approaches for stress reduction by highlighting the importance of a high-quality and coherent user experience. However, individual preferences regarding content motion and user interactivity in VR suggest that customizable VR environments may further enhance effectiveness. Advances in VR hardware and content development could facilitate such personalization, raising important questions about how to implement these as accessible, low-threshold tools for everyday stress management.

Despite these insights, a substantial proportion of the variance in intervention outcomes remains unexplained, indicating that the stress-reducing effects of VR are shaped by multiple interacting factors, including participant characteristics, intervention design, and technical features. Future RCTs should therefore systematically assess and report these variables to validate current findings, explore interdependencies, and establish evidence-based guidelines for optimizing VR-based stress reduction across diverse populations.

## Supplementary material

10.2196/78212 Multimedia Appendix 1 Complete search strings.

10.2196/78212 Multimedia Appendix 2 Overview on the different domains and applied labels.

10.2196/78212 Multimedia Appendix 3 Cross tables and chi-square test for variable distribution in separate subgroups.

10.2196/78212 Multimedia Appendix 4 Excluded studies from this systematic review.

10.2196/78212 Multimedia Appendix 5 Meta-analyses of different subgroups.

10.2196/78212 Multimedia Appendix 6 Forest and funnel plots for secondary outcomes.

10.2196/78212 Multimedia Appendix 7 Multivariate linear regression analyses and bubble plots.

10.2196/78212 Multimedia Appendix 8 Raw data analyzed in meta-analysis.

10.2196/78212 Checklist 1 PRISMA checklist.

10.2196/78212 Checklist 2 PRISMA-S checklist.

## Abbreviations

CAVE: cave automatic virtual environment
DASS: Depression, Anxiety, and Stress Scale
GRADE: Grading of Recommendations Assessment, Development, and Evaluation
HKSJ: Hartung, Knapp, Sidik, and Jonkman approach
HMD: head-mounted display
HR: heart rate
HRV: heart rate variability
PANAS: Positive and Negative Affect Schedule
PI: prediction interval
POMS: Profile of Mood States
PRESS: Peer Review of Electronic Search Strategies
PRISMA: Preferred Reporting Items for Systematic Reviews and Meta-Analyses
PRISMA-S: Extension to the PRISMA Statement for Reporting Literature Searches in Systematic Reviews
PSL: perceived stress level
RCT: randomized controlled trial
RoB: risk of bias
RWE: real-world exposure
SCL: skin conductance level
SMD: standardized mean difference
STAI-S: State Scale of State-Trait Anxiety Inventory
SYS: systolic blood pressure
TSM: traditional stress management
VR: virtual reality
