# Supplementary material for: Virtual Reality Interventions for Stress Reduction in the General Population: Systematic Review and Meta-Analysis of Randomized Controlled Trials
Source: J Med Internet Res. 2026 May 25;28:e78212. doi: 10.2196/78212 (PMC13200809; doi:10.2196/78212)
Supplement: Multimedia Appendix 1 [file jmir-v28-e78212-s001.docx]

### Multimedia Appendix 1

Complete search strategy for the database MEDLINE for the systematic review on VR interventions for stress reduction in general population.^a^

| ID | Search term |
| --- | --- |
|  |  |
| #1 | "virtual reality"[MeSH Terms] |
| #2 | "virtual reality"[All Fields] |
| #3 | "virtual reality"[Title/Abstract:~2] or "simulated reality"[Title/Abstract:~2] or "digital reality"[Title/Abstract:~2] or "spatial reality"[Title/Abstract:~2] |
| #4 | "virtual environment"[Title/Abstract:~2] or "simulated environment"[Title/Abstract:~2] or "immersive environment"[Title/Abstract:~2] or "digital environment"[Title/Abstract:~2] |
| #5 | "virtual nature"[Title/Abstract:~2] or "simulated nature"[Title/Abstract:~2] or "digital nature"[Title/Abstract:~2] or "computer-generated nature"[Title/Abstract:~2] |
| #6 | "virtual world"[Title/Abstract:~2] or "simulated world"[Title/Abstract:~2] or "immersive world"[Title/Abstract:~2] or "computer-generated world"[Title/Abstract:~2] |
| #7 | "virtual spaces"[Title/Abstract:~2] or "3D environment"[Title/Abstract:~2] or "virtual immersion"[Title/Abstract:~2] or "immersive technology"[Title/Abstract:~2] or "3D simulation"[Title/Abstract:~2] or "3D video"[Title/Abstract:~2] or "360-degree simulation"[Title/Abstract:~2] or "360-degree video"[Title/Abstract:~2] or "virtual exposure"[Title/Abstract:~2] or "virtual experience"[Title/Abstract:~2] or "immersive experience"[Title/Abstract:~2] or "simulated experience"[Title/Abstract:~2] |
| #8 | 1 or 2 or 3 or 4 or 5 or 6 or 7 |
| #9 | "stress, psychological"[MeSH Terms] or "Subjective Stress"[MeSH Terms] or "relaxation"[MeSH Terms] |
| #10 | "stress*"[Title/Abstract] or "distress"[Title/Abstract] or "coping"[Title/Abstract] or "relax*"[Title/Abstract] or "resilience"[Title/Abstract] or "restorat*"[Title/Abstract] or "meditat*"[Title/Abstract] or "positive affect"[Title/Abstract] or "negative affect"[Title/Abstract] |
| #11 | 9 or 10 |
| #12 | "randomized controlled trial"[Publication Type] or "controlled clinical trial"[Publication Type] |
| #13 | "placebo"[Title/Abstract] or "random*"[Title/Abstract] or "Trial"[Title/Abstract] or "within subject*"[Title/Abstract] or "between subject*"[Title/Abstract] or "drug therapy"[MeSH Subheading] |
| #14 | "control*"[Title/Abstract] or "comparison"[Title/Abstract] or "compare*"[Title/Abstract] or "study"[Title/Abstract] or "different"[Title/Abstract] or "experiment*"[Title/Abstract] |
| #15 | "participant*"[Title/Abstract] or "patient*"[Title/Abstract] or "group*"[Title/Abstract] or "intervention*"[Title/Abstract] or "individual*"[Title/Abstract] or "subject*"[Title/Abstract] or "therapy"[Title/Abstract] or "adult*"[Title/Abstract] |
| #16 | 14 and 15 |
| #17 | 12 or 13 or 16 |
| #18 | 8 and 11 and 17 |
| #19 | "animals"[MeSH Terms] |
| #20 | "humans"[MeSH Terms] |
| #21 | 19 not 20 |
| #22 | "review"[Publication Type] or "meta-analysis"[Publication Type] |
| #23 | 21 or 22 |
| #24 | 18 not 23 |

^a^Search conducted at 24/07/2024.

Complete search strategy for the database CENTRAL for the systematic review on VR interventions for stress reduction in general population.^a^

| ID | Search term |
| --- | --- |
|  |  |
| #1 | ((virtual NEAR/2 reality) OR (simulated NEAR/2 reality) OR (digital NEAR/2 reality) OR (spatial NEAR/2 reality) OR (virtual NEAR/2 environment) OR (simulated NEAR/2 environment) OR (digital NEAR/2 environment) OR (virtual NEAR/2 nature) OR (digital NEAR/2 nature) OR (simulated NEAR/2 nature) OR (computer-generated NEAR/2 nature) OR (immersive NEAR/2 environment) OR (virtual NEAR/2 spaces) OR (3d NEAR/2 environment) OR (virtual NEAR/2 immersion) OR (immersive NEAR/2 technology) OR (virtual NEAR/2 exposure) OR (3d NEAR/2 simulation) OR (3d NEAR/2 video) OR (360 degree NEAR/2 simulation) OR (360 degree NEAR/2 video) OR (virtual NEAR/2 world) OR (simulated NEAR/2 world) OR (immersive NEAR/2 world) OR (computer-generated NEAR/2 world) OR (virtual NEAR/2 experience) OR (immersive NEAR/2 experience) OR (simulated NEAR/2 experience)):ti,ab,kw |
| #2 | (stress* OR relax* OR coping OR distress OR resilience OR restorat* OR meditat* OR (positive affect) OR (negative affect)):ti,ab,kw |
| #3 | #1 AND #2 |

^a^Search conducted at 24/07/2024.

Complete search strategy for the database CINAHL for the systematic review on VR interventions for stress reduction in general population.^a^

| ID | Search term |
| --- | --- |
|  |  |
| #1 | MM (Stress) OR MM (Psychological stress) OR MM (Stress Management) OR MM (Psychological Distress) OR DE (stress) OR TI (stress* OR relax* OR coping OR distress OR resilience OR restorat* OR meditat*) OR AB (stress* OR relax* OR coping OR distress OR resilience OR restorat* OR meditat* OR (negative N1 affect) OR (positive N1 affect)) |
| #2 | MM (virtual reality) OR DE (virtual reality) OR DE (virtual environment) OR TI ((virtual N2 reality) OR (simulated N2 reality) OR (digital N2 reality) OR (spatial N2 reality) OR (virtual N2 environment) OR (simulated N2 environment) OR (immersive N2 environment) OR (virtual N2 spaces) OR (3d N2 environment) OR (digital N2 environment) OR (digital N2 nature) OR (virtual N2 nature) OR (simulated N2 nature) OR (computer-generated N2 nature) OR (virtual N2 immersion) OR (immersive N2 technology) OR (3d N2 simulation) OR (3d N2 video) OR (360-degree N2 video) OR (360-degree N2 simulation) OR (virtual N2 world) OR (simulated N2 world) OR (immersive N2 world) OR (computer-generated N2 world) OR (virtual N2 experience) OR (immersive N2 experience) OR (virtual N2 exposure) OR (simulated N2 experience) OR(immersive N2 technology)) OR AB((virtual N2 reality) OR (simulated N2 reality) OR (digital N2 reality) OR (spatial N2 reality) OR (virtual N2 environment) OR (simulated N2 environment) OR (digital N2 environment) OR (immersive N2 environment) OR (virtual N2 spaces) OR (3d N2 environment) OR (virtual N2 nature) OR (digital N2 nature) OR (simulated N2 nature) OR (computer-generated N2 nature) OR (virtual N2 immersion) OR (immersive N2 technology) OR (3d N2 simulation) OR (3d N2 video) OR (360-degree N2 video) OR (360-degree N2 simulation) OR (virtual N2 world) OR (simulated N2 world) OR (immersive N2 world) OR (computer-generated N2 world) OR (virtual N2 experience) OR (immersive N2 experience) OR (virtual N2 exposure) OR (simulated N2 experience) OR (immersive N2 technology)) |
| #3 | (PT (randomi?ed controlled trial OR randomi?ed clinical trial OR controlled trial) OR (DE “Placebo” OR DE “randomized controlled trials” OR DE “randomized clinical trials” OR DE “experiment controls”) OR MH (randomized controlled trials OR double‐blind studies OR single‐blind studies OR random assignment OR pretest‐posttest design) OR TI (randomi?ed N1 clinical N1 trial* OR randomi?ed N1 controlled N1 trial* OR rct OR controlled N1 trial* OR controlled N1clinical N1 trial*) OR AB (randomi?ed N1 clinical N1 trial* OR randomi?ed N1 controlled N1 trial* OR RCT OR random* OR controlled N1 trial* OR controlled N1 clinical N1 trial*) OR AB (random* OR between-subject* OR within-subject* OR placebo OR trial*) OR (AB (control* OR compare* OR comparison OR experiment* OR different OR study) AND AB (group* OR patient* OR participant* OR intervention* OR subject*OR therapy OR individual* OR adult*)) NOT (TI (animal* NOT hum?n) OR AB (animal* NOT hum?n) OR TI (meta analysis OR systematic* review) OR PT (metaanalysis OR systematic review) OR MH (metaanalysis OR systematic review)) |
| #4 | S1 AND S2 AND S3 |

^a^Search conducted at 24/07/2024.

Complete search strategy for the database PsycINFO for the systematic review on VR interventions for stress reduction in general population.^a^

| No | Search term |
| --- | --- |
|  |  |
| #1 | DE “Virtual Reality” OR DE “Virtual Environment” OR TI ((virtual N2 reality) OR (simulated N2 reality) OR (digital N2 reality) OR (spatial N2 reality) OR (virtual N2 environment) OR (simulated N2 environment) OR (immersive N2 environment) OR (virtual N2 spaces) OR (3d N2 environment) OR (digital N2 environment) OR (digital N2 nature) OR (virtual N2 nature) OR (simulated N2 nature) OR (computer-generated N2 nature) OR (virtual N2 immersion) OR (immersive N2 technology) OR (3d N2 simulation) OR (3d N2 video) OR (360-degree N2 video) OR (360-degree N2 simulation) OR (virtual N2 world) OR (simulated N2 world) OR (immersive N2 world) OR (computer-generated N2 world) OR (virtual N2 experience) OR (immersive N2 experience) OR (virtual N2 exposure) OR (simulated N2 experience) OR(immersive N2 technology)) OR AB((virtual N2 reality) OR (simulated N2 reality) OR (digital N2 reality) OR (spatial N2 reality) OR (virtual N2 environment) OR (simulated N2 environment) OR (digital N2 environment) OR (immersive N2 environment) OR (virtual N2 spaces) OR (3d N2 environment) OR (virtual N2 nature) OR (digital N2 nature) OR (simulated N2 nature) OR (computer-generated N2 nature) OR (virtual N2 immersion) OR (immersive N2 technology) OR (3d N2 simulation) OR (3d N2 video) OR (360-degree N2 video) OR (360-degree N2 simulation) OR (virtual N2 world) OR (simulated N2 world) OR (immersive N2 world) OR (computer-generated N2 world) OR (virtual N2 experience) OR (immersive N2 experience) OR (virtual N2 exposure) OR (simulated N2 experience) OR (immersive N2 technology)) |
| #2 | (MM Stress) OR (DE Psychological stress) OR (DE Perceived Stress) OR (DE Distress) OR (DE Stress Management) OR TI (stress* OR relax* OR coping OR distress OR resilience OR cope* OR restorat* OR meditat*) OR AB (stress* OR relax* OR coping OR distress OR resilience OR cope* OR restorat* OR meditat* OR (negative N1 affect) OR (positive N1 affect)) |
| #3 | (ZT (randomi?ed controlled trial OR randomi?ed clinical trial OR controlled trial) OR (DE “Placebo” OR DE “randomized controlled trials” OR DE “randomized clinical trials” OR DE “experiment controls”) OR MH (randomized controlled trials OR double‐blind studies OR single‐blind studies OR random assignment OR pretest‐posttest design) OR TI (randomi?ed N1 clinical N1 trial* OR randomi?ed N1 controlled N1 trial* OR rct OR controlled N1 trial* OR controlled N1clinical N1 trial*) OR AB (randomi?ed N1 clinical N1 trial* OR randomi?ed N1 controlled N1 trial* OR RCT OR random* OR controlled N1 trial* OR controlled N1 clinical N1 trial*) OR AB (random* OR between-subject* OR within-subject* OR placebo OR trial*) OR (AB (control* OR compare* OR comparison OR experiment* OR different OR study) AND AB (group* OR patient* OR participant* OR intervention* OR subject*OR therapy OR individual* OR adult*)) NOT (TI (animal* NOT hum?n) OR AB (animal* NOT hum?n) OR TI (meta-analysis OR systematic* review) OR MR (metaanalysis OR systematic* N1 review) OR ZT (metaanalysis OR systematic* N1 review)) |
| #4 | S1 AND S2 AND S3 |

^a^Search conducted at 24/07/2024.

Complete search strategy for the database WEB OF SCIENCE for the systematic review on VR interventions for stress reduction in general population.^a^

| No | Search term |
| --- | --- |
|  |  |
| #1 | TS=((virtual NEAR/2 reality) OR (simulated NEAR/2 reality) OR (digital NEAR/2 reality) OR (spatial NEAR/2 reality) OR (virtual NEAR/2 environment) OR (simulated NEAR/2 environment) OR (digital NEAR/2 environment) OR (immersive NEAR/2 environment) OR (virtual NEAR/2 spaces) OR (3D NEAR/2 environment) OR (virtual NEAR/2 nature) OR (simulated NEAR/2 nature) OR (computer-generated NEAR/2 nature) OR (digital NEAR/2 nature) OR (virtual NEAR/2 immersion) OR (immersive NEAR/2 technology) OR (3D NEAR/2 simulation) OR (3D NEAR/2 video) OR (360-degree NEAR/2 simulation) OR (360-degree NEAR/2 video) OR (virtual NEAR/2 exposure) OR (virtual NEAR/2 world) OR (simulated NEAR/2 world) OR (immersive NEAR/2 world) OR (computer-generated NEAR/2 world) OR (virtual NEAR/2 experience) OR (immersive NEAR/2 experience) OR (simulated NEAR/2 experience) OR (immersive NEAR/2 technology)) |
| #2 | TS=(relax* OR stress* OR distress OR coping OR resilience OR restorat* OR meditat* OR “positive affect” OR “negative affect”) |
| #3 | (TS=(randomized controlled trial* OR randomized clinical trial* OR RCT OR randomised controlled trial* OR randomised clinical trial* OR controlled clinical trial* OR random* OR between-subject* OR within-subject* OR placebo OR trial*) OR (TS=(control* OR compare* OR comparison OR study OR experiment* OR different) AND TS=(group* OR intervention* OR individual* OR subject* OR therapy OR participant* OR patient* OR adult*))) NOT (TS=(animal* NOT hum?n) OR TI=(meta-analysis OR systematic* review) OR AK=(meta-analysis OR systematic* review) OR KP=(meta-analysis OR systematic* review)) |
| #4 | #1 AND #2 AND #3 |

^a^Search conducted at 24/07/2024.
