# Supplementary material for: Virtual Reality Interventions for Stress Reduction in the General Population: Systematic Review and Meta-Analysis of Randomized Controlled Trials
Source: J Med Internet Res. 2026 May 25;28:e78212. doi: 10.2196/78212 (PMC13200809; doi:10.2196/78212)
Supplement: Multimedia Appendix 2 [file jmir-v28-e78212-s002.docx]

### Multimedia Appendix 2

Overview on the different domains and applied labels used to categorize the included studies (n=29) for subgroup analyses and meta regressions in the meta-analysis on VR interventions for stress reduction in general population.

| **Domain** | **Description** | **Labels** | **Criteria** |
| --- | --- | --- | --- |
|  |  |  |  |
| Setting | Categorization of the framework within which the study was conducted | General population | Studies that investigate stress reduction among healthy adults without special demands |
|  |  | Workplace/  University | Studies that research stress reduction in the framework of work- or study-related demands (e.g., during work breaks or among college students) |
|  |  | Clinical setting | Studies that research general stress reduction in a clinical setting among people with special demands (e.g., pregnant women or cancer patients), detached from disease treatment |
| Intervention type | Categorization of the provided method in VR environment for stress reduction | Nature exposure | Studies that utilize a pure VR exposure to natural environment without any tasks |
|  |  | Meditation | Studies that provide a guided meditation task (e.g., breathing meditation) in a natural VR environment |
|  |  | Game/activity | Studies that include an interactive task (e.g., coloring or music making) or game (e.g., Tetris or puzzle) as main VR content |
|  |  | Biophilic design | Studies that examine the effects of biophilic design elements (e.g., plants or green walls) in built environment (e.g., offices, streets, or courtyards) |
| Environment realism | Differentiation of VR environment in natural real images, recorded by camera, or more unrealistic computer-simulated images | Real world recording | Studies that present real world recordings in form of photos or videos captured by camera as VR environment |
|  |  | Computer simulation | Studies that present a computer-simulated environment, probably perceived as more unrealistic. |
| Environment style | Categorization of environment style provided in the VR intervention, based on involved natural elements and scenery | Green space | Studies that use environments with primarily green elements (e.g., park, garden, or forest) |
|  |  | Blue space | Studies that use environments dominated by water and sparsely green elements (e.g., beach or island) |
|  |  | Underwater | Studies that use an underwater scene |
|  |  | Biophilic architecture | Studies that provide biophilic elements (e.g., green walls or plants) in an otherwise rather urban or grey environment (e.g., office, courtyard, or street) |
|  |  | Different landscapes | Studies that provide changing environments of different labels |
| Content motion | Categorization of movement level of the VR environment due to animated or moving elements, cinematography, or other effects | No motion | VR interventions that provide static 3D photos or images without any animated or moving elements |
|  |  | Low | VR environments that provide a static scene from a consistent perspective with some moving elements like leaves in the wind (e.g., video recording on a tripod) |
|  |  | High | VR environments that provide a dynamic scene with changing perspective and, consequently, the impression to move or walk in the virtual world (e.g., video recording of a pathway) |
| User interactivity | Categorization of the level of user interactivity provided in the VR intervention, expressed by the given option to control and manipulate the VR content | Low | Interventions with low control of the virtual environment modulation, given by the option to explore the delivered content within the field of view by head movement |
|  |  | Medium | Interventions with medium control of the virtual environment modulation, given by the option to change the perspective while moving in the environment by controller or body movement |
|  |  | High | Interventions with high control of the virtual environment modulation, given by the option to interact with elements due to an interactive task or game |
