# Supplementary material for: Virtual Reality Interventions for Stress Reduction in the General Population: Systematic Review and Meta-Analysis of Randomized Controlled Trials
Source: J Med Internet Res. 2026 May 25;28:e78212. doi: 10.2196/78212 (PMC13200809; doi:10.2196/78212)
Supplement: Multimedia Appendix 3 [file jmir-v28-e78212-s003.docx]

### Multimedia Appendix 3

### Cross tables and ꭓ²-statistics performed in the overall dataset

Table S1. Cross table for the distribution of different predictors in subgroups based on the domain “setting” in the overall dataset, investigated in the framework of the meta-analysis on VR interventions for stress reduction in general population.

|  | **General population** | | | **Workplace/University** | | | **Clinical setting** | | | **General** | |
| --- | --- | --- | --- | --- | --- | --- | --- | --- | --- | --- | --- |
|  | O | E | Se_i_ | O | E | Se_i_ | O | E | Se_i_ | O | E |
|  |  |  |  |  |  |  |  |  |  |  |  |
| **Intervention type*** |  |  |  |  |  |  |  |  |  |  |  |
| Nature exposure | 7 | 3.8 | 1.7 | 3 | 7.0 | -1.5 | 4 | 3.2 | 0.4 | 14 | 14.0 |
| Meditation | 0 | 1.6 | -1.3 | 4 | 3.0 | 0.6 | 2 | 1.4 | 0.5 | 6 | 6.0 |
| Game/activity | 0 | 0.3 | -0.5 | 1 | 0.5 | 0.7 | 0 | 0.2 | -0.5 | 1 | 1.0 |
| Biophilic design | 0 | 1.3 | -1.2 | 5 | 2.5 | 1.6 | 0 | 1.2 | -1.1 | 5 | 5.0 |
| General | 7 | 7.0 |  | 13 | 13.0 |  | 6 | 6.0 |  | 26 | 26.0 |
| **Population*** |  |  |  |  |  |  |  |  |  |  |  |
| General population | 7 | 2.7 | 2.7 | 2 | 5.0 | -1.3 | 1 | 234 | -0.9 | 10 | 10.0 |
| Students | 0 | 3.5 | -1.9 | 13 | 6.5 | 2.5 | 0 | 3.0 | -1.7 | 13 | 13.0 |
| Pregnant women | 0 | 0.8 | -0.9 | 0 | 1.5 | -1.2 | 3 | 0.7 | 2.7 | 3 | 3.0 |
| Clinical residents | 0 | 0.8 | -0.9 | 0 | 1.5 | -1.2 | 3 | 0.7 | 2.7 | 3 | 3.0 |
| Older adults | 1 | 0.3 | 1.4 | 0 | 0.5 | -0.7 | 0 | 0.2 | -0.5 | 1 | 1.0 |
| General | 8 | 8.0 |  | 15 | 15.0 |  | 7 | 7.0 |  | 30 | 30.0 |
| **Sex dominance** |  |  |  |  |  |  |  |  |  |  |  |
| Predominately men | 1 | 1.7 | -0.5 | 4 | 3.1 | 0.5 | 1 | 1.2 | -0.2 | 6 | 6.0 |
| Predominately women | 7 | 6.3 | 0.3 | 11 | 11.9 | -0.3 | 5 | 4.8 | 0.1 | 23 | 23.0 |
| General | 8 | 8.0 |  | 15 | 15.0 |  | 6 | 6.0 |  | 29 | 29.0 |
| **Intervention duration*** |  |  |  |  |  |  |  |  |  |  |  |
| Single-session | 8 | 5.6 | 1.0 | 12 | 10.5 | 0.5 | 1 | 4.9 | -1.8 | 21 | 21.0 |
| Multi-session | 0 | 2.4 | -1.5 | 3 | 4.5 | -0.7 | 6 | 2.1 | 2.7 | 9 | 9.0 |
| General | 8 | 8.0 |  | 15 | 15.0 |  | 7 | 7.0 |  | 30 | 30.0 |
| **Age group*** |  |  |  |  |  |  |  |  |  |  |  |
| <40 years | 6 | 6.3 | -0.1 | 14 | 11.1 | 0.9 | 3 | 5.6 | -1.1 | 23 | 23.0 |
| ≥40 years | 2 | 1.7 | 0.3 | 0 | 2.9 | -1.7 | 4 | 1.4 | 2.1 | 6 | 6.0 |
| General | 8 | 8.0 |  | 14 | 14.0 |  | 7 | 7.0 |  | 29 | 29.0 |
| **Content motion** |  |  |  |  |  |  |  |  |  |  |  |
| No motion | 0 | 1.3 | -1.1 | 3 | 1.9 | 0.8 | 1 | 0.9 | 0.2 | 4 | 4.0 |
| Low | 6 | 4.2 | 0.9 | 5 | 6.2 | -0.5 | 2 | 2.6 | -0.4 | 13 | 13.0 |
| High | 2 | 2.6 | -0.4 | 4 | 3.8 | 0.1 | 2 | 1.6 | 0.3 | 8 | 8.0 |
| General | 8 | 8.0 |  | 12 | 12.0 |  | 5 | 5.0 |  | 25 | 25.0 |
| **User interactivity** |  |  |  |  |  |  |  |  |  |  |  |
| Low | 6 | 4.8 | 0.6 | 7 | 8.2 | -0.4 | 2 | 2.0 | 0.0 | 15 | 15.0 |
| Medium | 1 | 1.9 | -0.7 | 4 | 3.3 | 0.4 | 1 | 0.5 | 0.6 | 6 | 6.0 |
| High | 0 | 0.3 | -0.6 | 1 | 0.5 | 0.6 | 0 | 0.1 | -0.4 | 1 | 1.0 |
| General | 7 | 7.0 |  | 12 | 12.0 |  | 1 | 1.0 |  | 22 | 22.0 |
| **Environment realism** |  |  |  |  |  |  |  |  |  |  |  |
| Real world recording | 5 | 3.8 | 0.8 | 3 | 5.3 | -1.0 | 4 | 3.1 | 0.5 | 12 | 12.0 |
| Computer simulation | 3 | 4.4 | -0.7 | 9 | 6.7 | 0.9 | 3 | 3.9 | -0.5 | 15 | 15.0 |
| General | 8 | 8.0 |  | 12 | 12.0 |  | 7 | 7.0 |  | 27 | 27.0 |

*significant ꭓ²-tests for subgroups

Table S2. Cross table for the distribution of different predictors in subgroups based on the domain “intervention type” in the overall dataset, performed in the framework of the meta-analysis on VR interventions for stress reduction in general population.

|  | **Nature exposure** | | | **Meditation** | | | **Game/Activity** | | | **Biophilic design** | | | **General** | |
| --- | --- | --- | --- | --- | --- | --- | --- | --- | --- | --- | --- | --- | --- | --- |
|  | O | E | Se_i_ | O | E | Se_i_ | O | E | Se_i_ | O | E | Se_i_ | O | E |
|  |  |  |  |  |  |  |  |  |  |  |  |  |  |  |
| **Environment style*** |  |  |  |  |  |  |  |  |  |  |  |  |  |  |
| Green space | 8 | 6.0 | 0.8 | 2 | 2.5 | -0.3 | 1 | 0.5 | 0.7 | 0 | 2.0 | -1.4 | 11 | 11.0 |
| Blue space | 3 | 1.6 | 1.1 | 0 | 0.7 | -0.8 | 0 | 0.1 | -0.4 | 0 | 0.5 | -0.7 | 3 | 3.0 |
| Biophilic elements | 0 | 2.2 | -1.5 | 0 | 0.9 | -1.0 | 0 | 0.2 | -0.4 | 4 | 0.7 | 3.8 | 4 | 4.0 |
| Diverse landscapes | 1 | 2.2 | -0.8 | 3 | 0.9 | 2.2 | 0 | 0.2 | -0.4 | 0 | 0.7 | -0.9 | 4 | 4.0 |
| General | 12 | 12.0 |  | 5 | 5.0 |  | 1 | 1.0 |  | 4 | 4.0 |  | 22 | 22.0 |
| **Intervention duration** |  |  |  |  |  |  |  |  |  |  |  |  |  |  |
| Single-session | 12 | 12.2 | -0.1 | 4 | 6.1 | -0.8 | 1 | 0.7 | 0.4 | 6 | 4.1 | 1.0 | 23 | 23.0 |
| Multi-session | 6 | 5.8 | 0.1 | 5 | 2.9 | 1.2 | 0 | 0.3 | -0.6 | 0 | 1.9 | -1.4 | 11 | 11.0 |
| General | 18 | 18.0 |  | 9 | 9.0 |  | 1 | 1.0 |  | 6 | 6.0 |  | 34 | 34.0 |
| **Age group** |  |  |  |  |  |  |  |  |  |  |  |  |  |  |
| <40 years | 13 | 14.1 | -0.3 | 5 | 5.5 | -0.2 | 1 | 0.8 | 0.2 | 6 | 4.7 | 0.6 | 25 | 25.0 |
| ≥40 years | 5 | 3.9 | 0.5 | 2 | 1.5 | 0.4 | 0 | 0.2 | -0.5 | 0 | 1.3 | -1.1 | 7 | 7.0 |
| General | 18 | 18.0 |  | 7 | 7.0 |  | 1 | 1.0 |  | 6 | 6.0 |  | 27 | 27.0 |
| **Content motion*** |  |  |  |  |  |  |  |  |  |  |  |  |  |  |
| No motion | 1 | 2.5 | -0.9 | 0 | 0.6 | -0.8 | 0 | 0.2 | -0.4 | 3 | 0.8 | 2.5 | 4 | 4.0 |
| Low | 10 | 8.6 | 0.5 | 4 | 2.2 | 1.3 | 0 | 0.5 | -0.7 | 0 | 2.7 | -1.6 | 14 | 14.0 |
| High | 5 | 4.9 | 0.0 | 0 | 1.2 | -1.1 | 1 | 0.3 | 1.2 | 2 | 1.5 | 0.4 | 8 | 8.0 |
| General | 16 | 16.0 |  | 4 | 4.0 |  | 1 | 1.0 |  | 5 | 5.0 |  | 26 | 26.0 |
| **User interactivity*** |  |  |  |  |  |  |  |  |  |  |  |  |  |  |
| Low | 10 | 10.6 | -0.2 | 7 | 4.9 | 0.9 | 0 | 0.7 | -0.8 | 2 | 2.8 | -0.5 | 19 | 19.0 |
| Medium | 4 | 3.3 | 0.4 | 0 | 1.6 | -1.2 | 0 | 0.2 | -0.5 | 2 | 0.9 | 1.2 | 6 | 6.0 |
| High | 1 | 1.1 | -0.1 | 0 | 0.5 | -0.7 | 1 | 0.1 | 3.4 | 0 | 0.3 | -0.5 | 2 | 2.0 |
| General | 16 | 16.0 |  | 4 | 4.0 |  | 1 | 1.0 |  | 5 | 5.0 |  | 27 | 27.0 |
| **Environment realism*** |  |  |  |  |  |  |  |  |  |  |  |  |  |  |
| Real world recording | 14 | 8.2 | 2.0 | 0 | 3.6 | -1.9 | 0 | 0.5 | -0.7 | 1 | 2.7 | -1.0 | 15 | 15.0 |
| Computer simulation | 4 | 9.8 | -1.9 | 8 | 4.4 | 1.7 | 1 | 0.5 | 0.6 | 5 | 3.3 | 1.0 | 18 | 18.0 |
| General | 18 | 18.0 |  | 8 | 8.0 |  | 1 | 1.0 |  | 6 | 6.0 |  | 33 | 33.0 |

*significant ꭓ²-tests for subgroups

Table S3. Significant results of the ꭓ²-tests for subgroups based on the domain “setting” in the overall dataset, performed in the framework of the meta-analysis on VR interventions for stress reduction in general population.

|  | ꭓ² | df | P | Cramér’s V |
| --- | --- | --- | --- | --- |
|  |  |  |  |  |
| Target population | 45.07 | 8 | <.001 | 0.87 |
| Age group | 9.41 | 2 | =.009 | 0.57 |
| Intervention duration | 14.49 | 2 | <.001 | 0.70 |
| Intervention type | 13.46 | 6 | =.04 | 0.51 |

Table S4. Significant results of ꭓ²-tests for subgroups based on the domain “intervention type” in the overall dataset, performed in the framework of the meta-analysis on VR interventions for stress reduction in general population.

|  | ꭓ² | df | P | Cramér’s V |
| --- | --- | --- | --- | --- |
|  |  |  |  |  |
| Environment realism | 17.09 | 3 | <.001 | 0.72 |
| Environment style | 30.13 | 9 | <.001 | 0.68 |
| Content motion | 16.07 | 6 | =.01 | 0.56 |
| User interactivity | 17.54 | 6 | =.007 | 0.57 |
