# Supplementary material for: Virtual Reality Interventions for Stress Reduction in the General Population: Systematic Review and Meta-Analysis of Randomized Controlled Trials
Source: J Med Internet Res. 2026 May 25;28:e78212. doi: 10.2196/78212 (PMC13200809; doi:10.2196/78212)
Supplement: Multimedia Appendix 5 [file jmir-v28-e78212-s005.docx]

# Multimedia Appendix 5

## Meta-analyses for separate subgroups based on study design characteristics

Table S1. Separately conducted meta-analyses for the intervention effects on perceived stress for diverse subgroups based on study design, investigated in the framework of the meta-analysis on VR interventions for stress reduction in general population.^a-b^

|  | Subgroup | SMD | 95% CI | P | I² | 95% PI | n |
| --- | --- | --- | --- | --- | --- | --- | --- |
|  |  |  |  |  |  |  |  |
| **Setting** |  |  |  |  |  |  |  |
|  | General population | -0.54 | -0.70; -0.37 | <.001 | 75% | -1.28; 0.21 | 31 |
|  | Clinical setting | -0.59 | -1.04; -0.15 | =.02 | 80% | -1.80; 0.60 | 8 |
| **Target group** |  |  |  |  |  |  |  |
|  | General population | -0.29 | -0.47; -0.11 | =.005 | 57% | -0.77; 0.20 | 13 |
|  | Students | -0.68 | -0.92; -0.45 | <.001 | 70% | -1.50; 0.13 | 18 |
|  | Pregnant women | -0.46 | -1.86; -0.94 | =.29 | 85% | -3.12; 2.20 | 3 |
|  | Clinical residents | -0.84 | -1.60; -0.08 | =.04 | 73% | -2.34; 0.66 | 4 |
| **Age group** |  |  |  |  |  |  |  |
|  | < 40 years | -0.54 | -0.71; -0.37 | <.001 | 76% | -1.33; 0.46 | 30 |
|  | ≥ 40 years | -0.63 | -1.11; -0.16 | =.02 | 79% | -1.83; 0.56 | 7 |
| **Gender ratio** |  |  |  |  |  |  |  |
|  | Men dominance | -0.49 | -0.76; -0.23 | =.003 | 59% | -1.16; 0.17 | 8 |
|  | Women dominance | -0.55 | -0.75; -0.35 | <.001 | 82% | -1.48; 0.38 | 28 |
| **Intervention duration** |  |  |  |  |  |  |  |
|  | Single-session intervention | -0.46 | -0.62; -0.31 | <.001 | 72% | -1.13; 0.20 | 27 |
|  | Multi-session intervention | -0.77 | -1.13; -0.41 | <.001 | 78% | -1.89; 0.35 | 12 |

^a^Meta-analyses for different subgroups were conducted by Hartung-Knapp-Sidik-Jonkman method in RevMan, Version 9.11.0.

^b^Effect sizes of different VR interventions of one publication were pooled for main analyses and are included as one intervention group [56,65,68,73,89,100]. In subgroup analyses and meta regressions studies were excluded if categorization was not applicable.

Figure S1. Intervention effects on perceived stress and heterogeneity for subgroups based on target group, investigated in the framework of the meta-analysis on VR interventions for stress reduction in general population.^a-c^


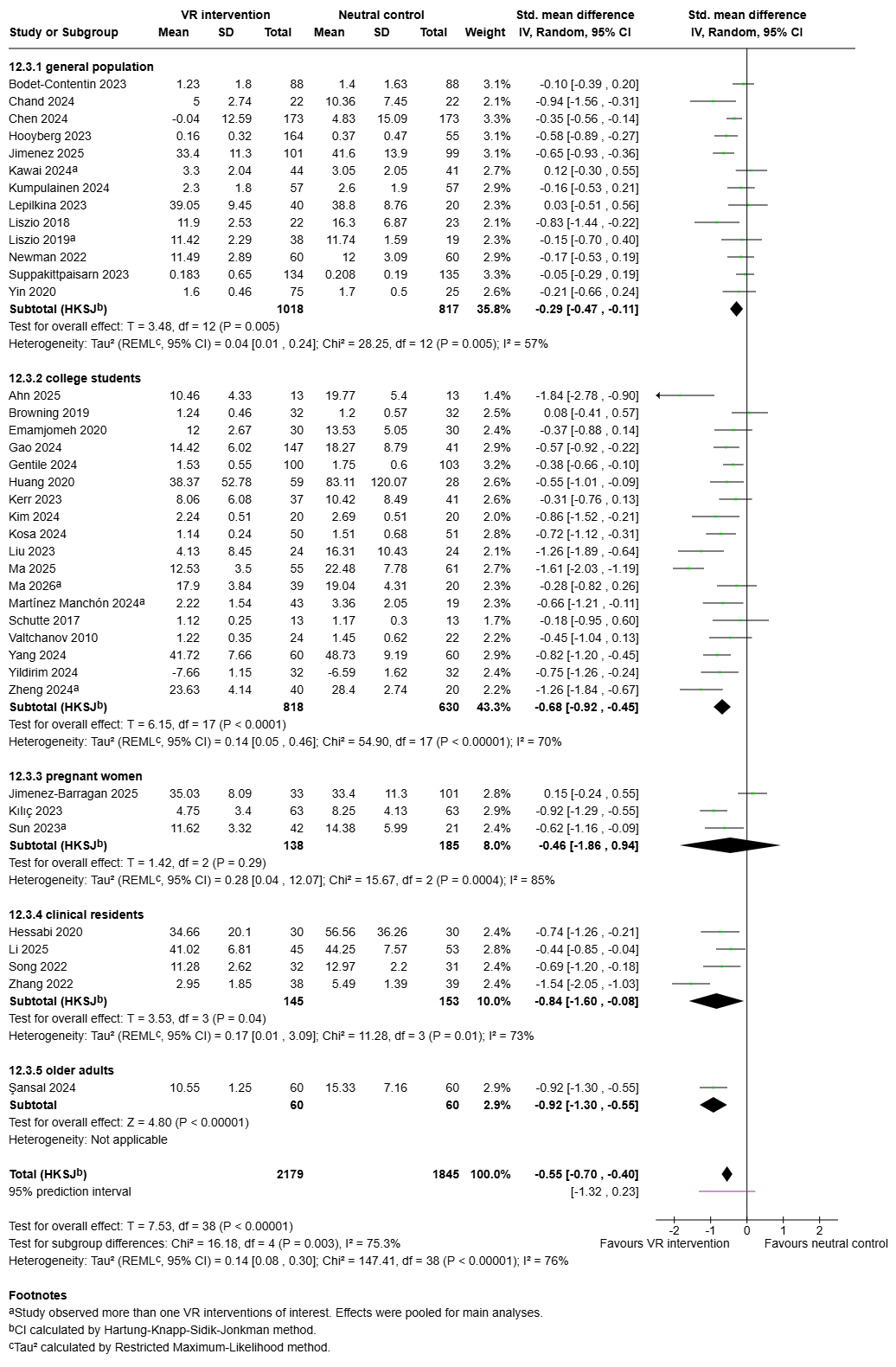


Figure S2. Intervention effects on perceived stress and heterogeneity for subgroups based on intervention duration, investigated in the framework of the meta-analysis on VR interventions for stress reduction in general population.^a-c^


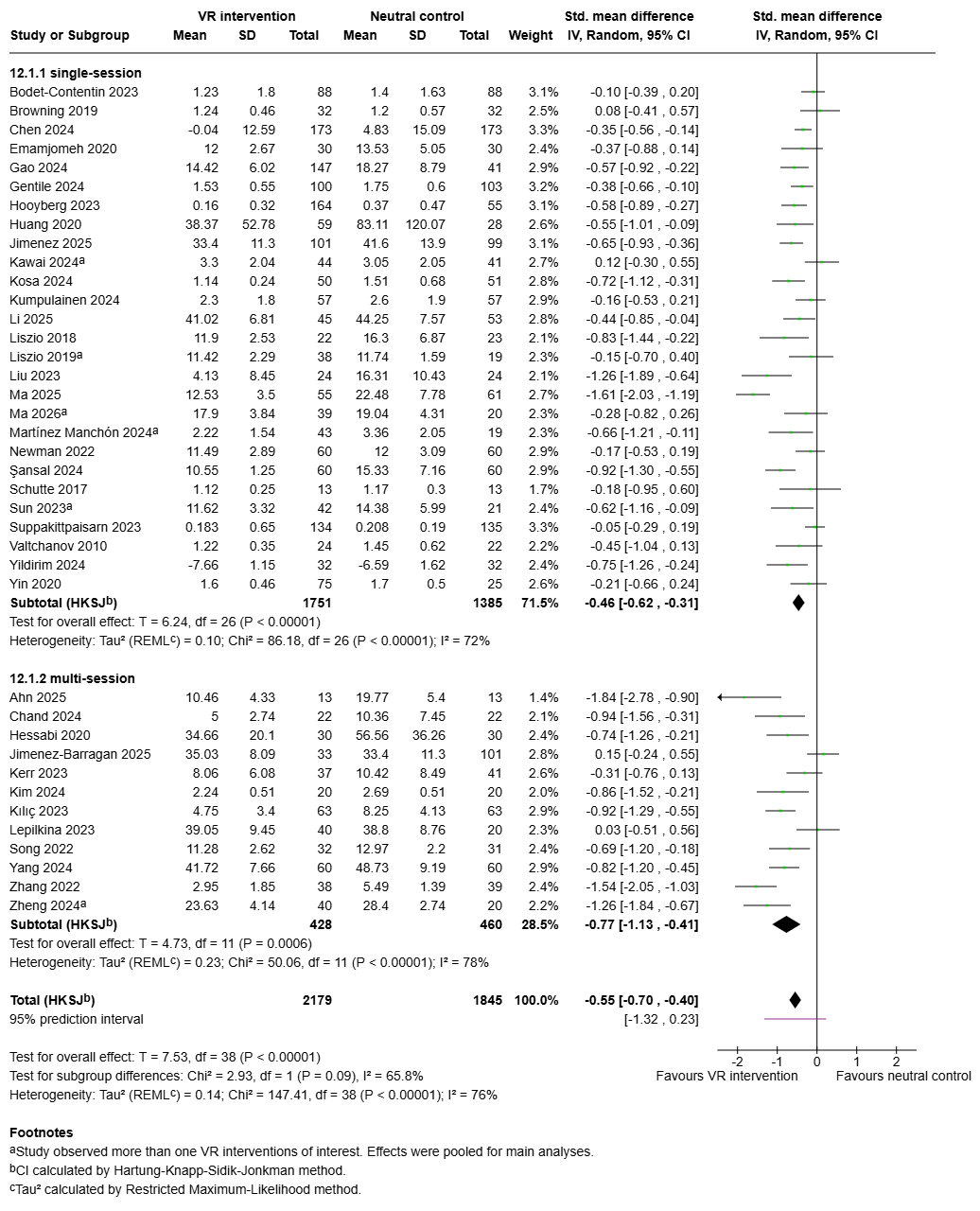


Figure S3. Intervention effects on perceived stress and heterogeneity for subgroups based on setting, investigated in the framework of the meta-analysis on VR interventions for stress reduction in general population.^a-c^

*
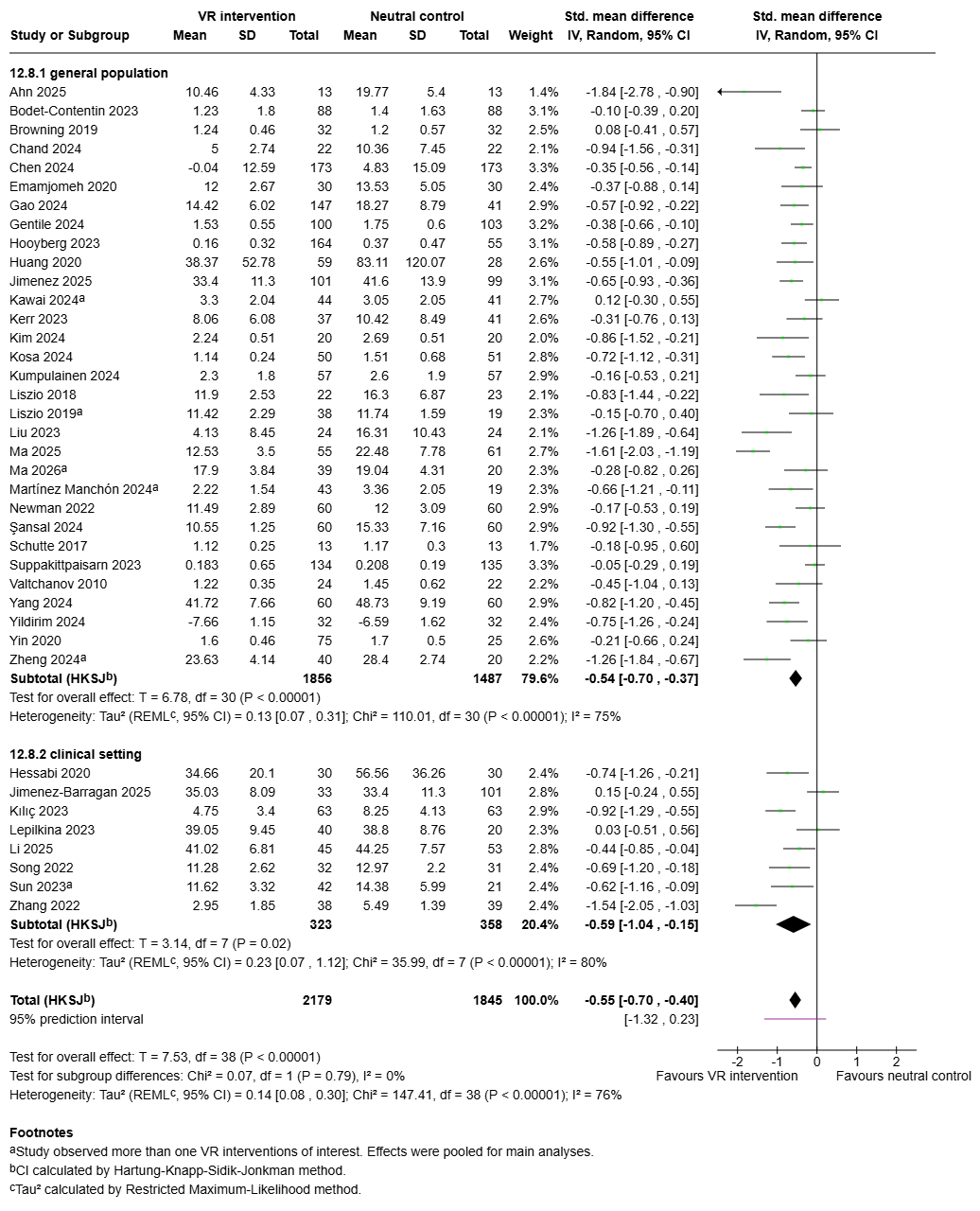
*

Figure S4. Intervention effects on perceived stress and heterogeneity for subgroups based on age group, investigated in the framework of the meta-analysis on VR interventions for stress reduction in general population.^a-c^

*
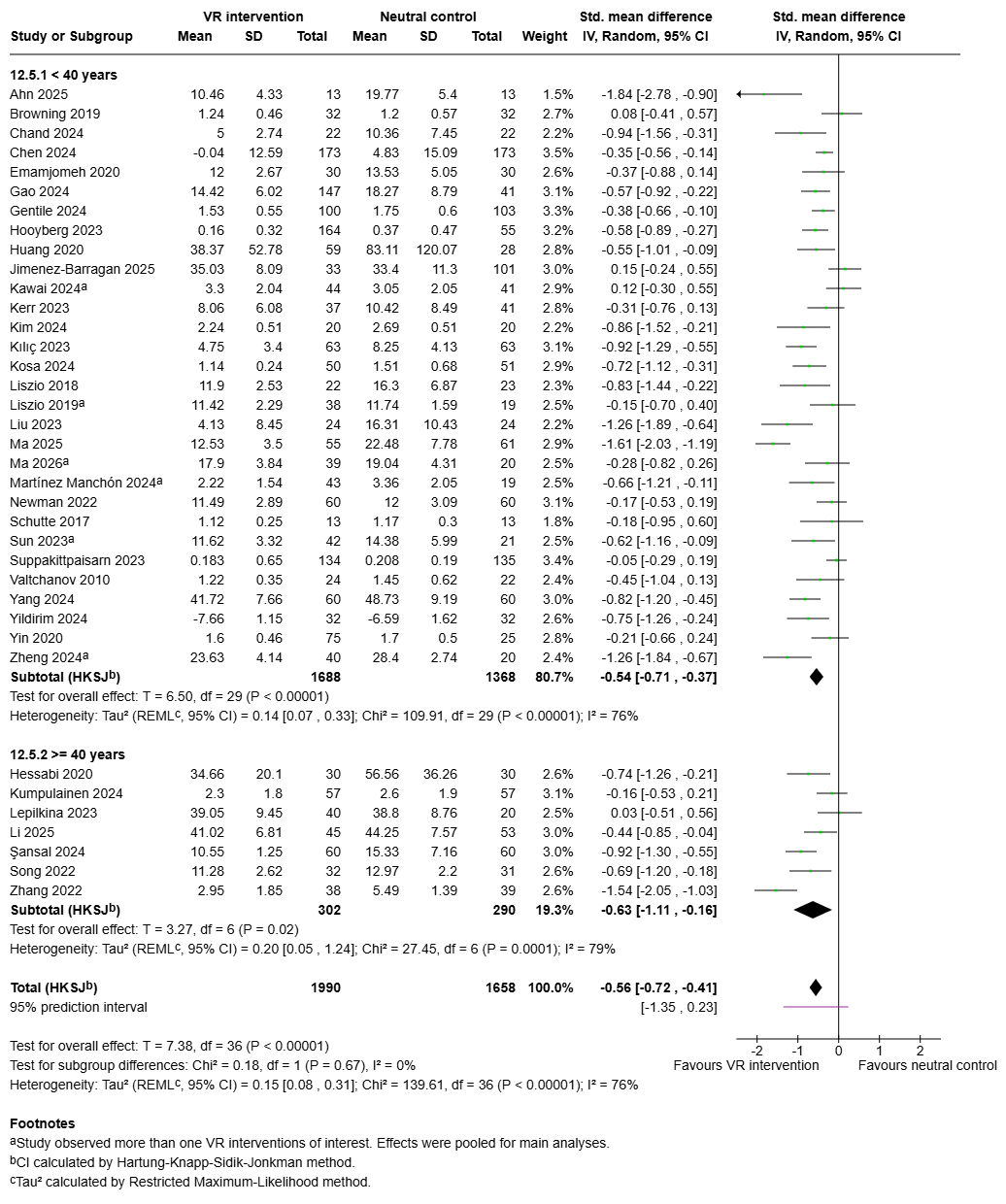
*

Figure S5. Intervention effects on perceived stress and heterogeneity for subgroups based on gender ratio, investigated in the framework of the meta-analysis on VR interventions for stress reduction in general population.^a-c^


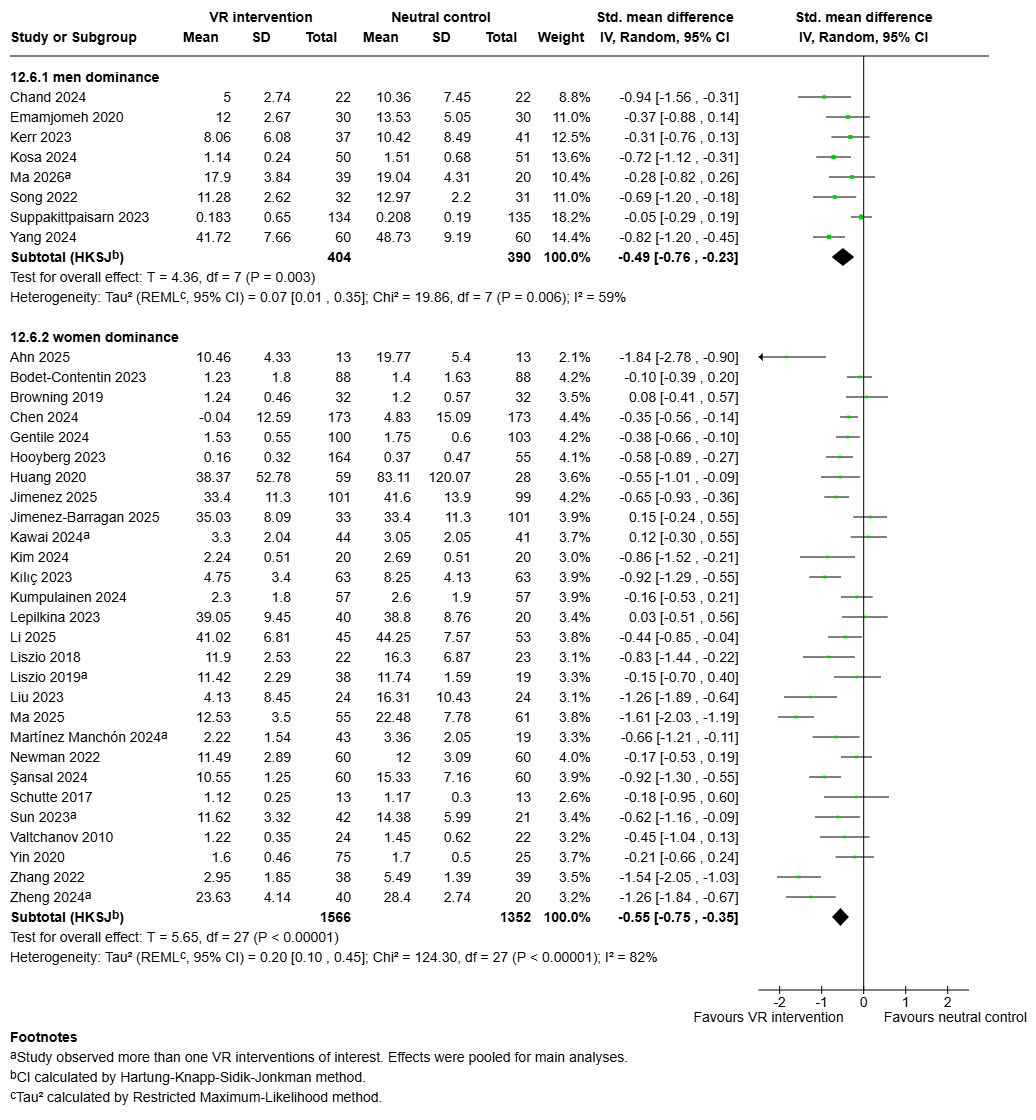


## Meta-analyses for separate subgroups based on technical and content-related characteristics of the VR intervention

Table S2. Separately conducted meta-analyses for the intervention effects on perceived stress for diverse subgroups based on intervention characteristics, investigated in the framework of the meta-analysis on VR interventions for stress reduction in general population.^a-d^

|  | Subgroup | SMD | 95% CI | P | I² | 95% PI | n |
| --- | --- | --- | --- | --- | --- | --- | --- |
|  |  |  |  |  |  |  |  |
| **Intervention type** |  |  |  |  |  |  |  |
|  | Meditation | -0.31 | -0.57; -0.05 | =.03 | 60% | -0.92; 0.30 | 8 |
|  | Nature exposure | -0.53 | -0.79; -0.28 | <.001 | 81% | -1.43; 0.37 | 17 |
|  | Biophilic design | -0.54 | -0.92; -0.16 | =.02 | 43% | -1.23; 0.15 | 6 |
|  | Game/activity | -1.16 | -6.84; 4.51 | =.23 | 89% | -10.62; 8.30 | 2 |
| **Environment realism** |  |  |  |  |  |  |  |
|  | Real world recording | -0.55 | -0.78; -0.32 | <.001 | 75% | -1.34; 0.24 | 17 |
|  | Computer simulation | -0.50 | -0.71; -0.29 | <.001 | 77% | -1.35; 0.35 | 20 |
| **Content motion^c^** |  |  |  |  |  |  |  |
|  | no motion | -0.45 | -0.78; -0.11 | =.02 | 0% | -0.78; -0.11 | 4 |
|  | low | -0.42 | -0.68; -0.17 | =.004 | 75% | -1.17; 0.33 | 14 |
|  | high | -0.78 | -1.22; -0.34 | =.004 | 85% | -2.07; 0.51 | 9 |
| **Content motion (dichotomy)** |  |  |  |  |  |  |  |
|  | static | -0.40 | -0.59; -0.22 | <.001 | 63% | -0.98; 0.17 | 19 |
|  | moving | -0.78 | -1.22; -0.34 | =.004 | 85% | -2.07; 0.51 | 9 |
| **User interactivity^b^** |  |  |  |  |  |  |  |
|  | low | -0.43 | -0.60; -0.27 | <.001 | 66% | -1.01; 0.15 | 20 |
|  | medium | -0.70 | -1.30; -0.11 | =.03 | 83% | -2.18; 0.77 | 7 |
|  | high | -0.74 | -2.89; 1.41 | =.28 | 94% | -4.93; 3.46 | 3 |
| **User interactivity (dichotomy)** |  |  |  |  |  |  |  |
|  | passive | -0.43 | -0.60; -0.27 | <.001 | 66% | -1.01; 0.15 | 20 |
|  | active | -0.72 | -1.20; -0.24 | =.008 | 87% | -2.16; 0.73 | 10 |

^a^Different VR conditions of interest of one publication are included as pooled effect size [56,65,68,73,89,100]. If intervention arms differ in a domain and could not be categorized, the study was excluded from analyses.

^b^User interactivity was ranked as 1=“low control” (only watching and exploring scene by head movement), 2=“medium control” (option to change perspective while moving in environment by controller or body movement), or 3=“high control” (option to interact with elements due to an interactive task or game).

^c^Content motion was categorized as 1=“no motion” (simple 3D images without moving elements), 2=“low motion” (static scene with some moving elements), or 3=“high motion” (dynamic scene with changing perspective).

^d^Meta-analyses for different subgroups were conducted by Hartung-Knapp-Sidik-Jonkman method in RevMan, Version 9.11.0.

Figure S6. Intervention effects on perceived stress and heterogeneity for subgroups based on content motion, investigated in the framework of the meta-analysis on VR interventions for stress reduction in general population.^a-c^
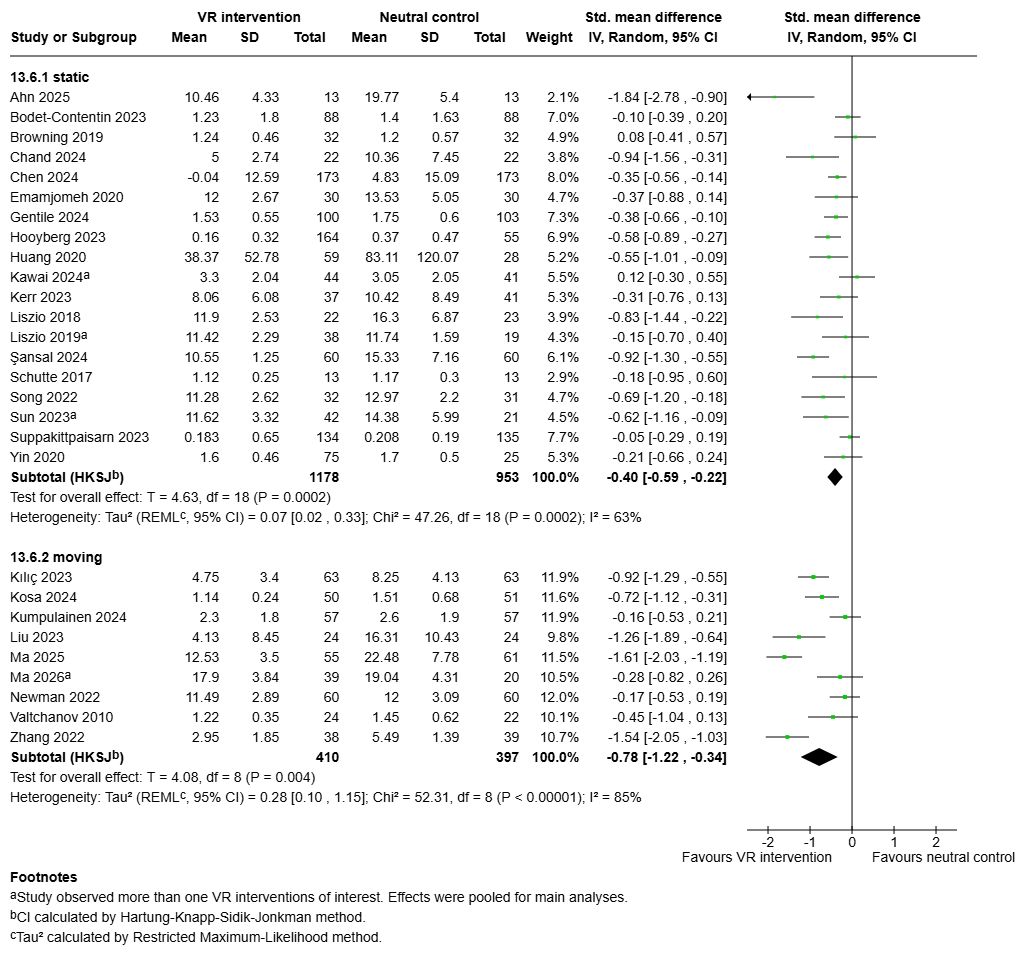


Figure S7. Intervention effects on perceived stress and heterogeneity for subgroups based on user interactivity, investigated in the framework of the meta-analysis on VR interventions for stress reduction in general population.^a-c^

*
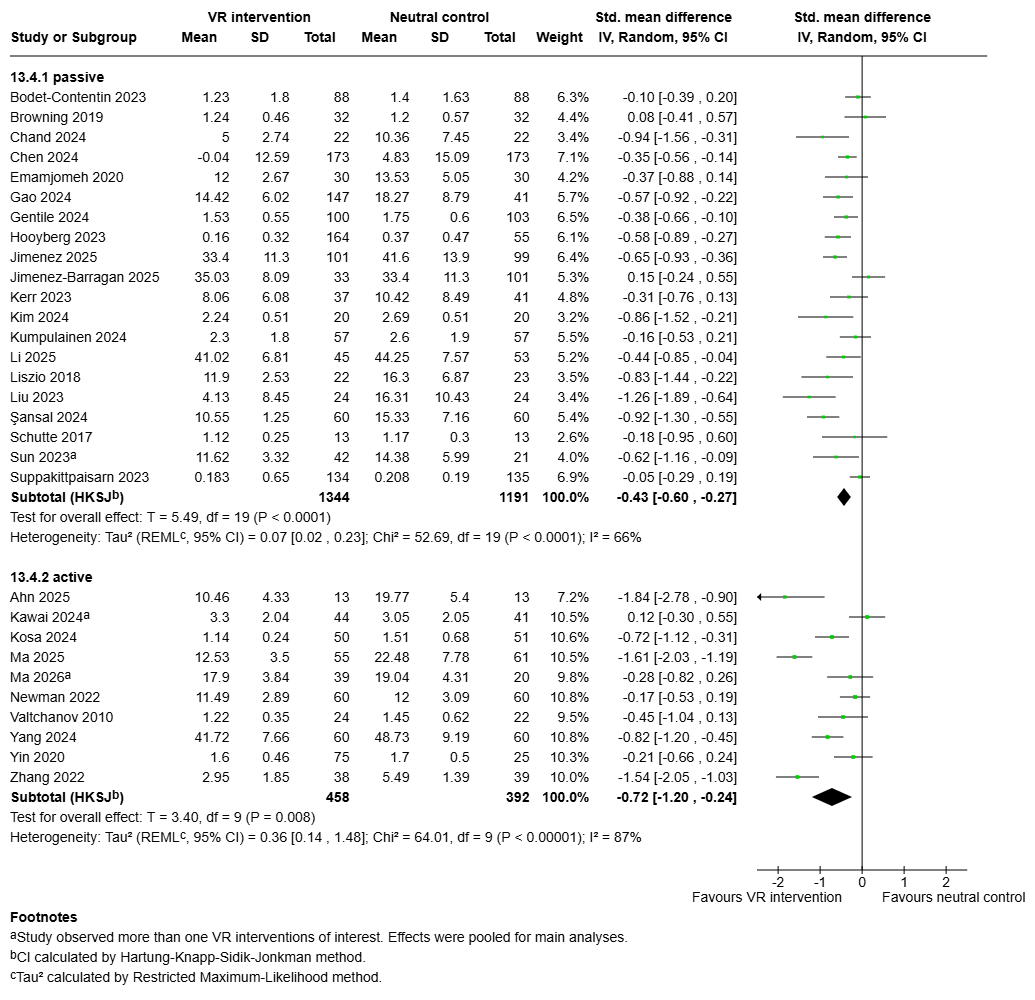
*

Figure S8. Intervention effects on perceived stress and heterogeneity for subgroups based on intervention type, investigated in the framework of the meta-analysis on VR interventions for stress reduction in general population.^a-c^


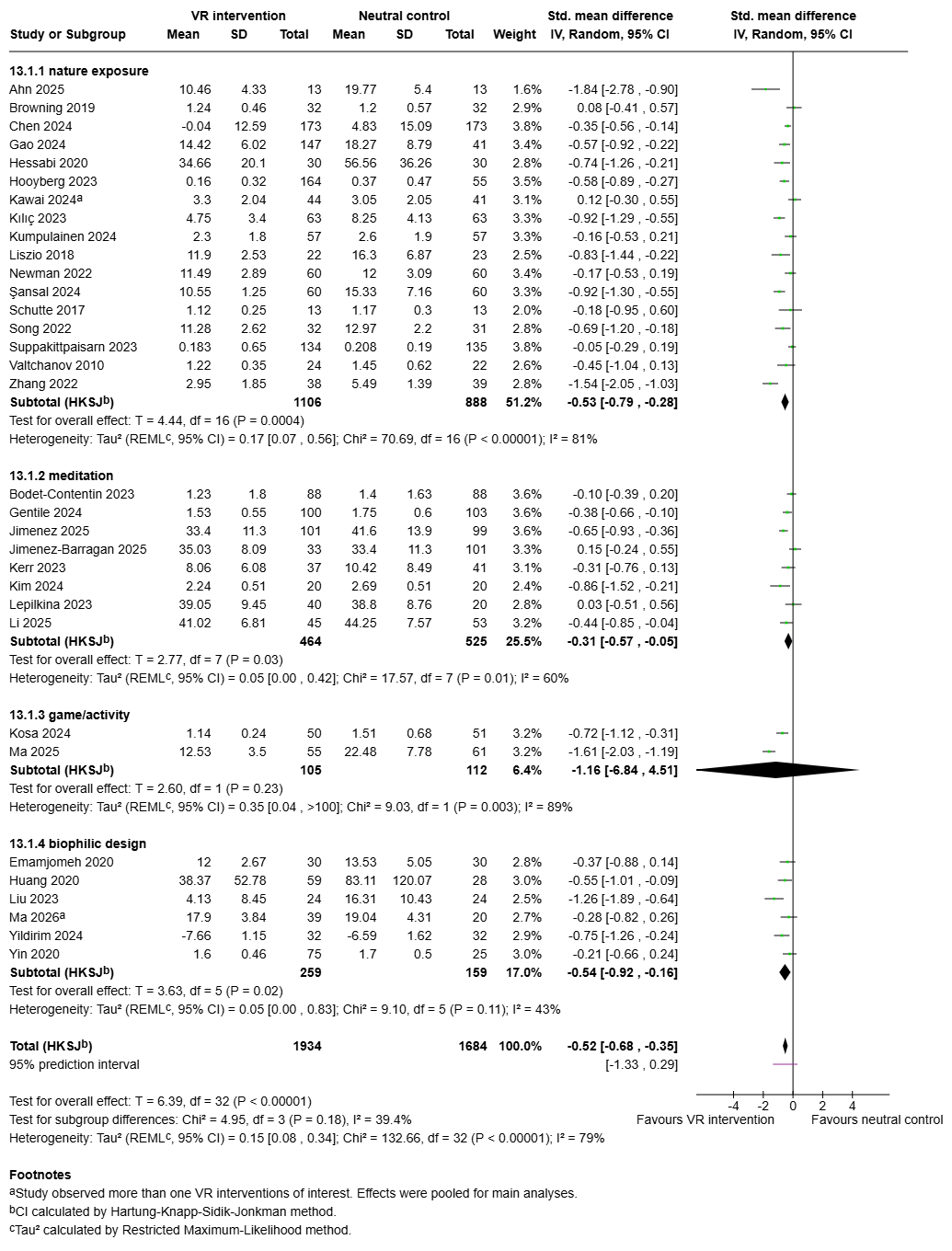


Figure S9. Intervention effects on perceived stress and heterogeneity for subgroups based on environment realism, investigated in the framework of the meta-analysis on VR interventions for stress reduction in general population.^a-c
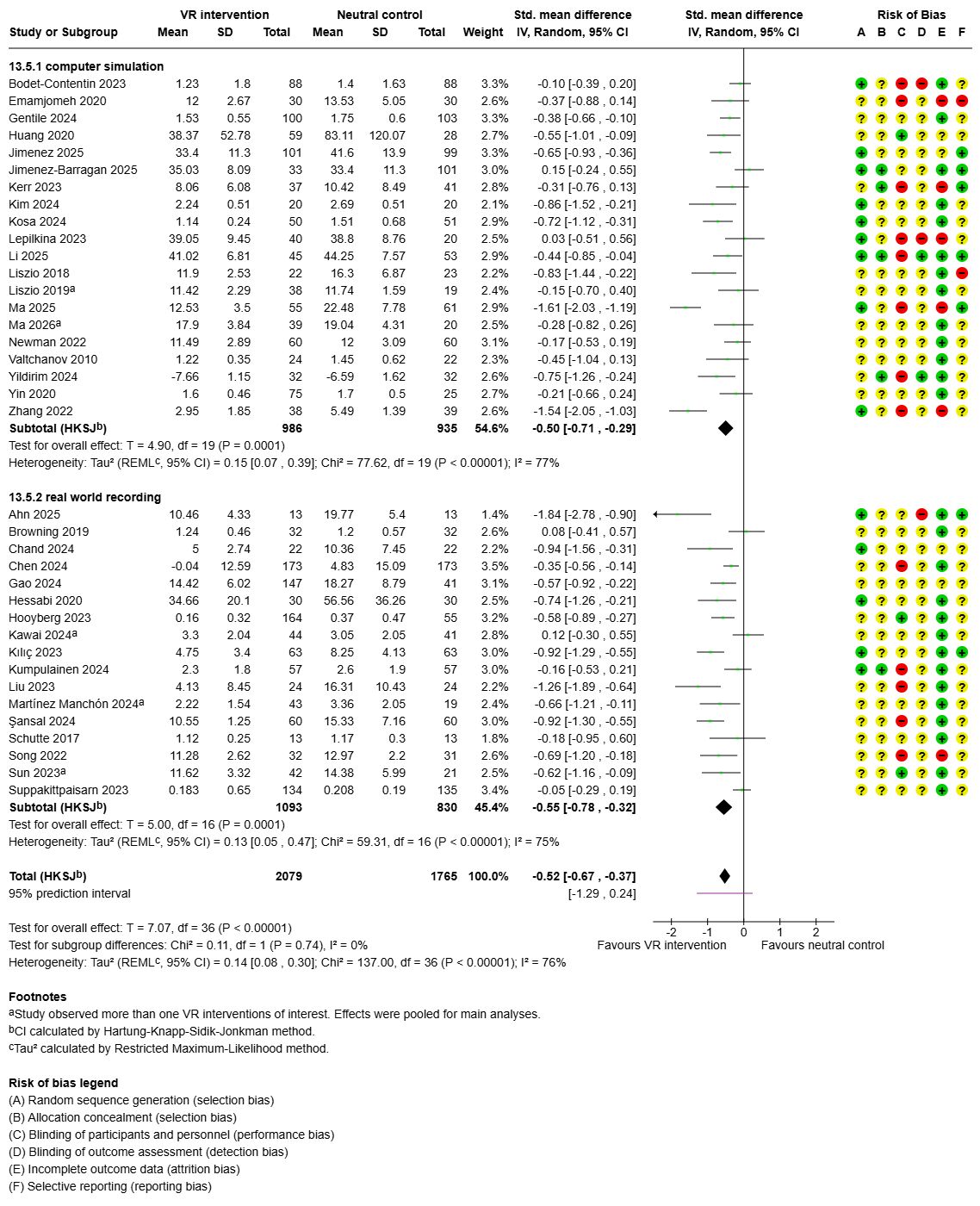
^
