# Supplementary material for: Virtual Reality Interventions for Stress Reduction in the General Population: Systematic Review and Meta-Analysis of Randomized Controlled Trials
Source: J Med Internet Res. 2026 May 25;28:e78212. doi: 10.2196/78212 (PMC13200809; doi:10.2196/78212)
Supplement: Multimedia Appendix 6 [file jmir-v28-e78212-s006.docx]

# Multimedia Appendix 6

## Forest plots for secondary physiological outcomes

Figure S1. Forest plot for heart rate outcome (post-intervention) of the meta-analysis on VR interventions for stress reduction in the general population, generated using RevMan and including effect sizes for separate studies.

^
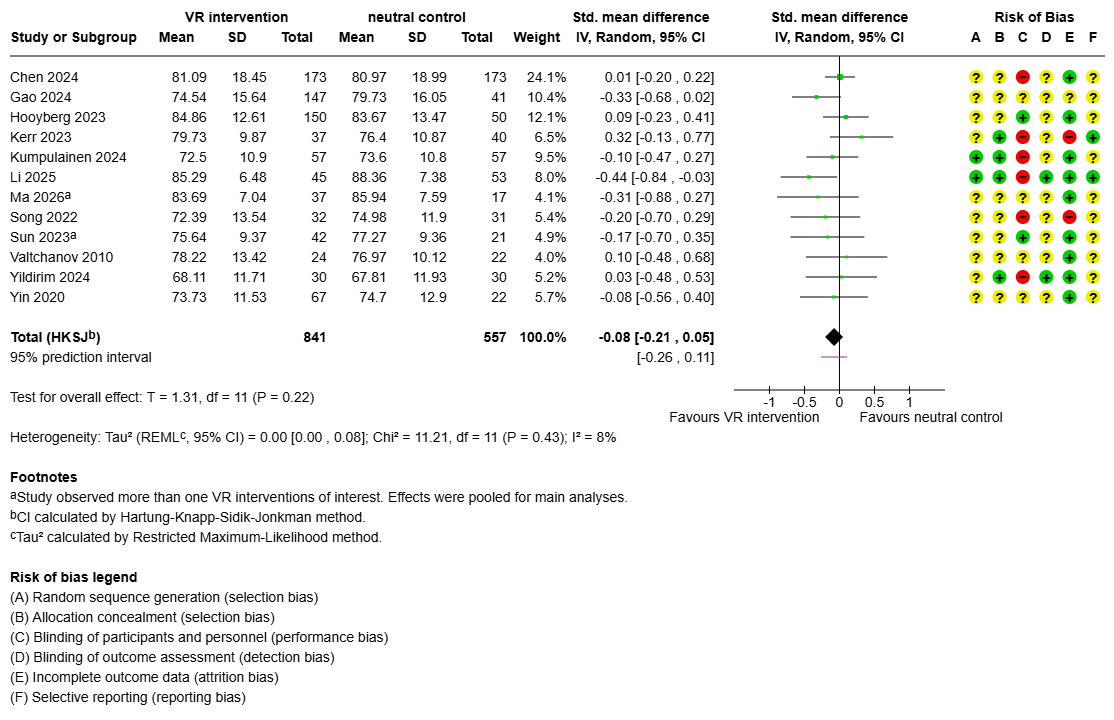
^

Figure S2. Forest plot for heart rate variability outcome (post-intervention) of the meta-analysis on VR interventions for stress reduction in the general population, generated using RevMan and including effect sizes for separate studies.


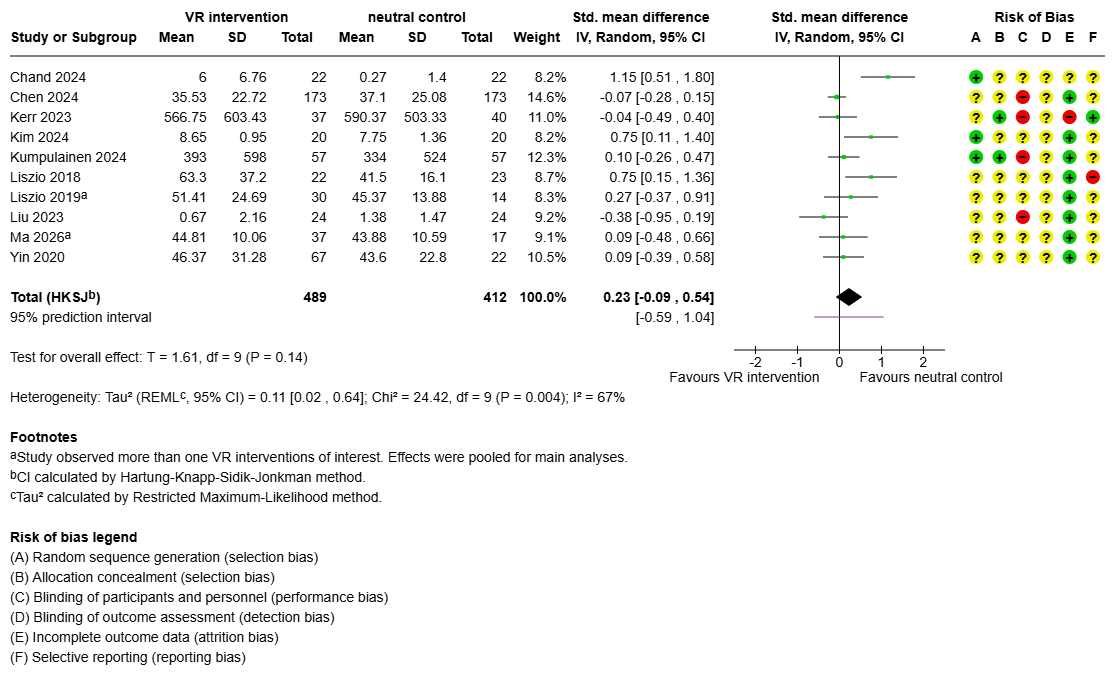


Figure S3. Forest plot for skin conductance level outcome (post-intervention) of the meta-analysis on VR interventions for stress reduction in the general population, generated using RevMan and including effect sizes for separate studies.


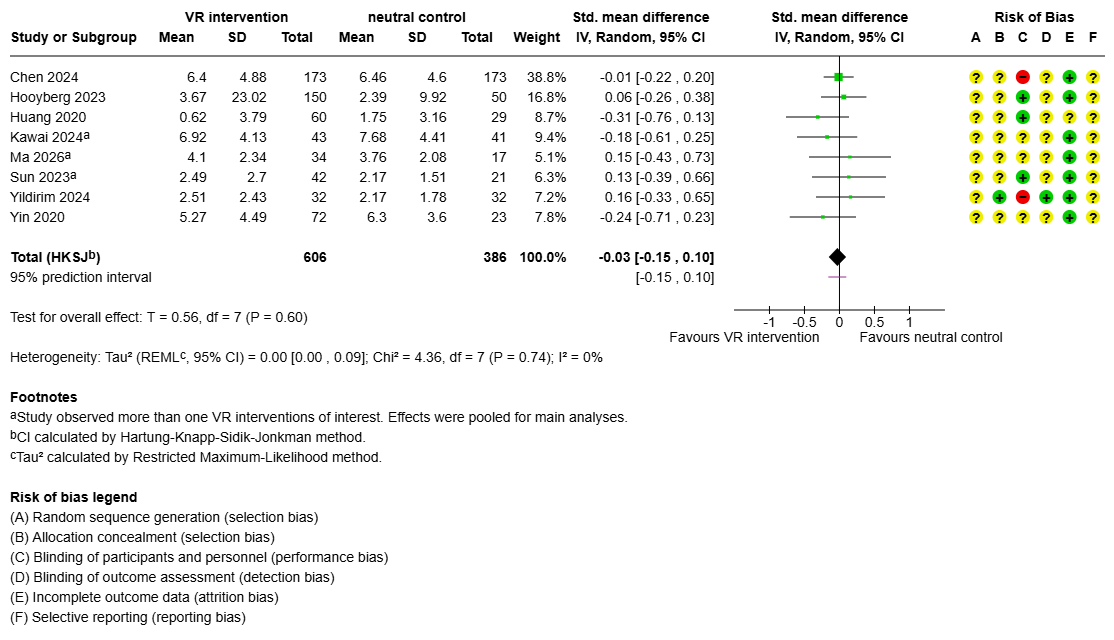


Figure S4. Forest plot for systolic blood pressure outcome (post-intervention) of the meta-analysis on VR interventions for stress reduction in the general population, generated using RevMan and including effect sizes for separate studies.


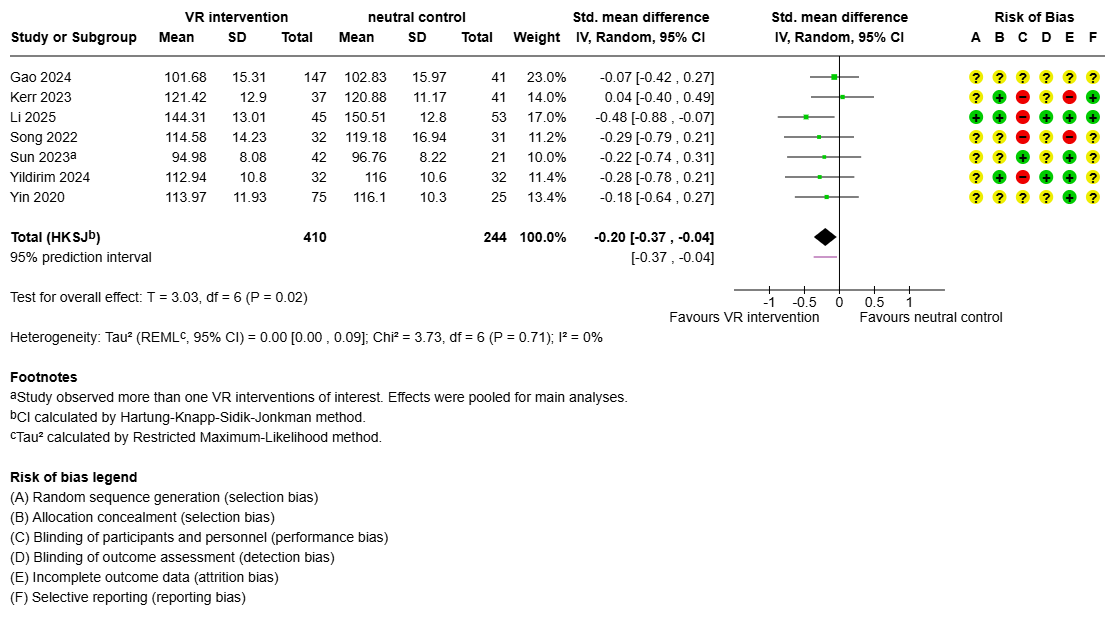


## Forest plots for secondary psychological outcomes

Figure S5. Forest plot for positive emotion outcome (post-intervention) of the meta-analysis on VR interventions for stress reduction in the general population, generated using RevMan and including effect sizes for separate studies.

##
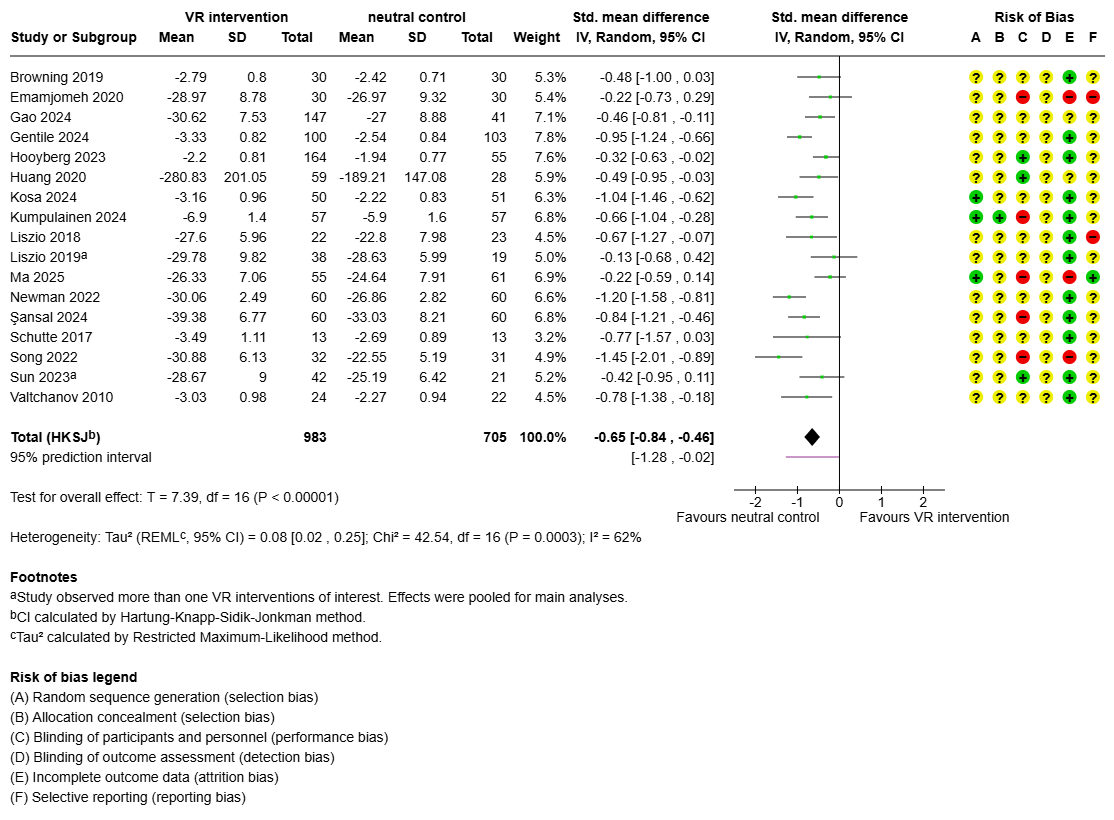


Figure S6. Forest plot for anxiety outcome (post-intervention) of the meta-analysis on VR interventions for stress reduction in the general population, generated using RevMan and including effect sizes for separate studies.


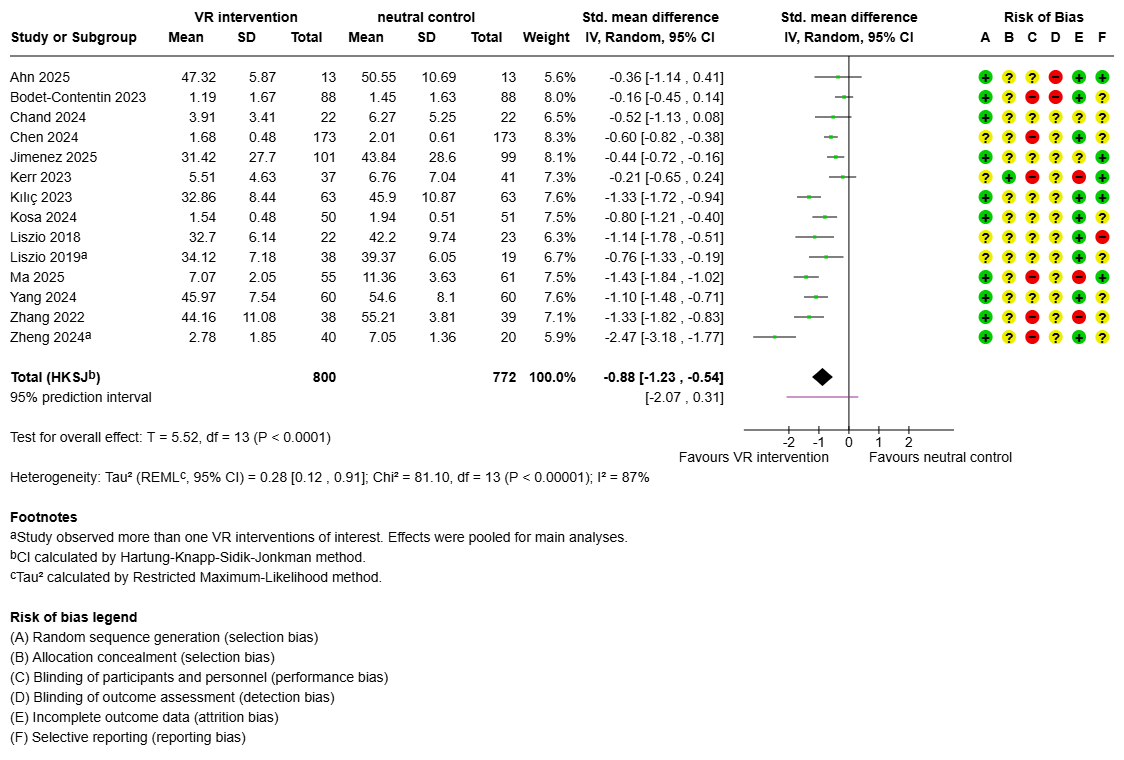


Figure S7. Forest plot for depression outcome (post-intervention) of the meta-analysis on VR interventions for stress reduction in the general population, generated using RevMan and including effect sizes for separate studies.


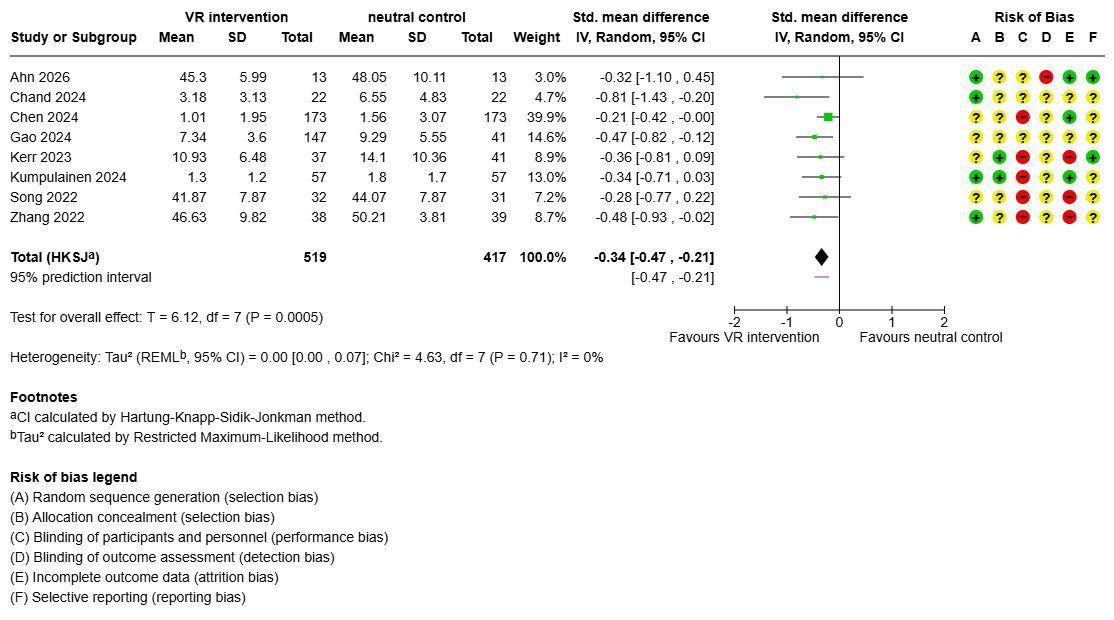


Figure S8. Forest plot for restoration outcome (post-intervention) of the meta-analysis on VR interventions for stress reduction in the general population, generated using RevMan and including effect sizes for separate studies.


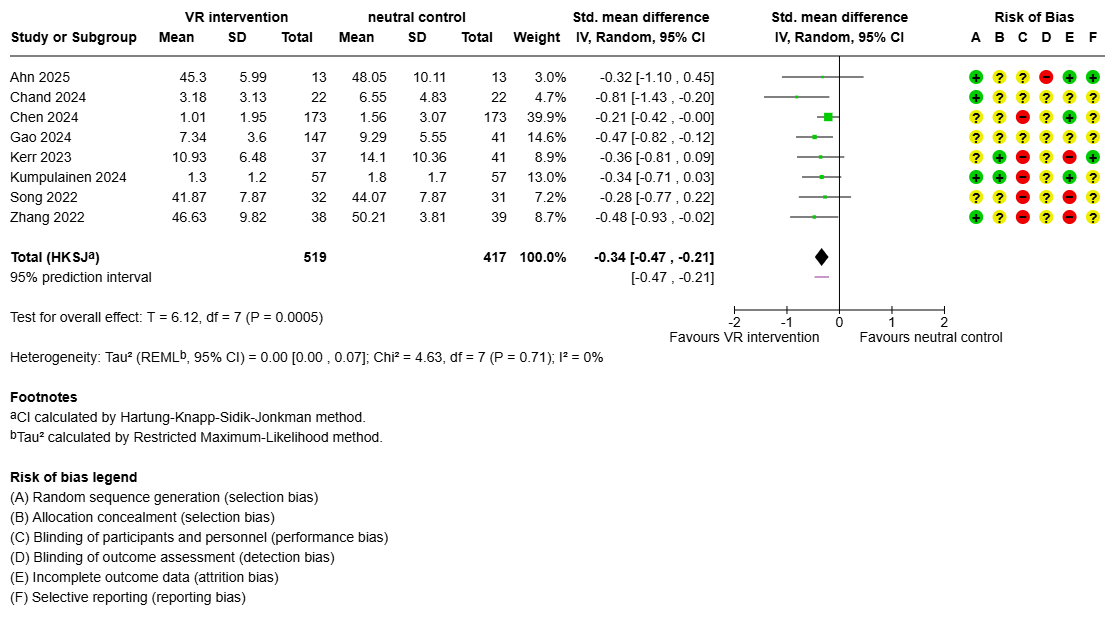


## Funnel plots for secondary physiological outcomes

Figure S9. Funnel plot for heart rate outcome of the meta-analysis on VR interventions for stress reduction in the general population, generated using RevMan.^a^


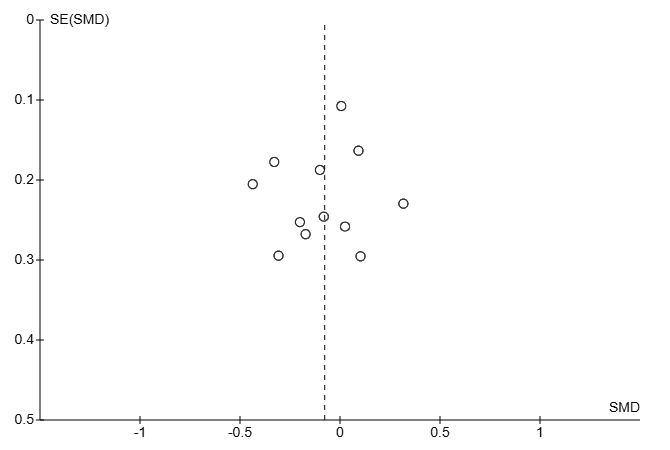


^a^Egger’s test for funnel plot asymmetry (conducted in SPSS): Intercept=-0.1, CI -0.71 to 0.51, P=.73

Figure S10. Funnel plot for heart rate variability outcome of the meta-analysis on VR interventions for stress reduction in the general population, generated using RevMan. ^a^


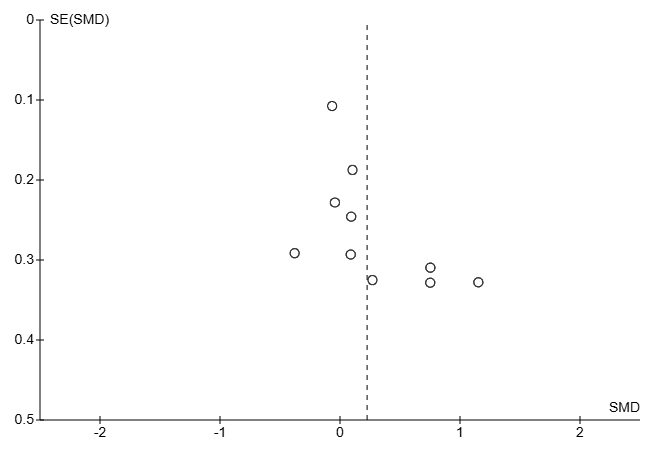


^a^Egger’s test for funnel plot asymmetry (conducted in SPSS): Intercept=1.13, CI -0.27 to 2.54, P=.10

Figure S11. Funnel plot for skin conductance level outcome of the meta-analysis on VR interventions for stress reduction in the general population, generated using RevMan. ^a^
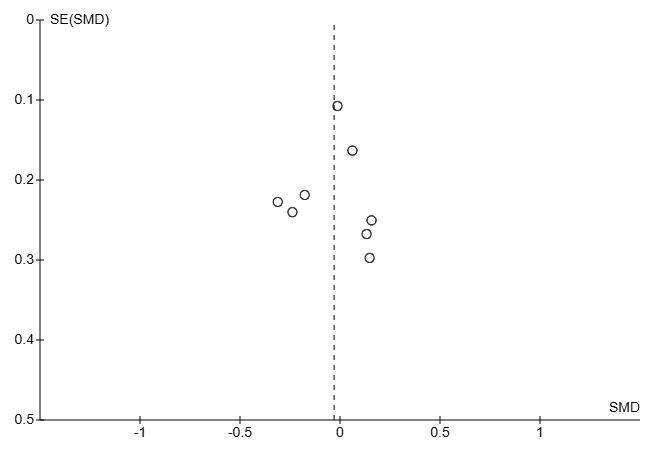


^a^Egger’s test for funnel plot asymmetry (conducted in SPSS): Intercept=0.17, CI -0.57 to 0.91, P=.60

Figure S12. Funnel plot for systolic blood pressure outcome of the meta-analysis on VR interventions for stress reduction in the general population, generated using RevMan. ^a^
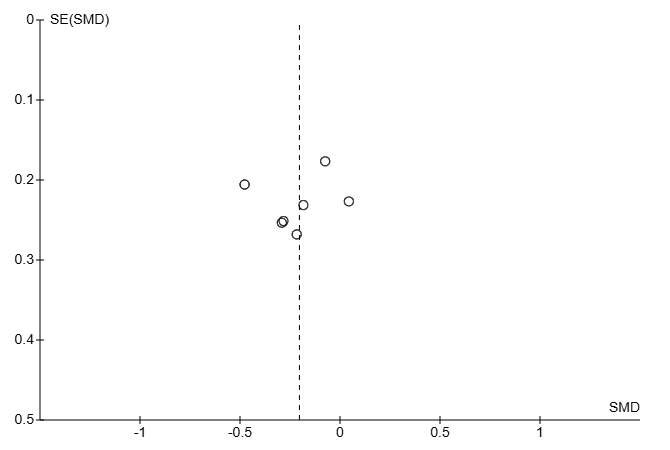


^a^Egger’s test for funnel plot asymmetry (conducted in SPSS): Intercept=-0.41, CI -1.69 to 0.87, P=.45

## Funnel plots for secondary psychological outcomes

Figure S13. Funnel plot for positive emotion outcome of the meta-analysis on VR interventions for stress reduction in the general population, generated using RevMan. ^a^


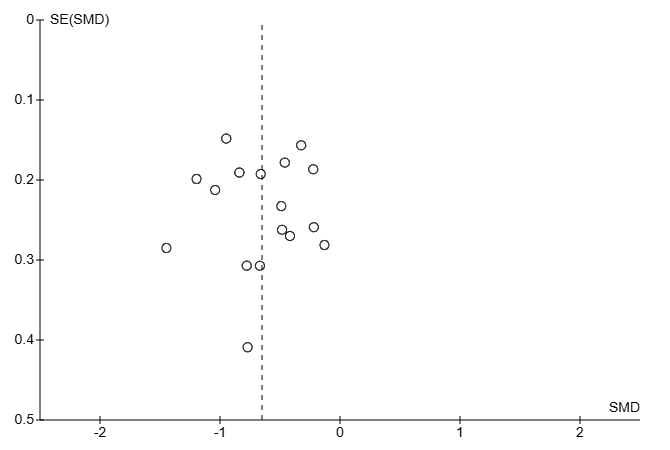


^a^Egger’s test for funnel plot asymmetry (conducted in SPSS): Intercept=-0.77, CI -1.51 to -0.04, P=.04

Figure S14. Funnel plot for anxiety outcome of the meta-analysis on VR interventions for stress reduction in the general population, generated using RevMan. ^a^
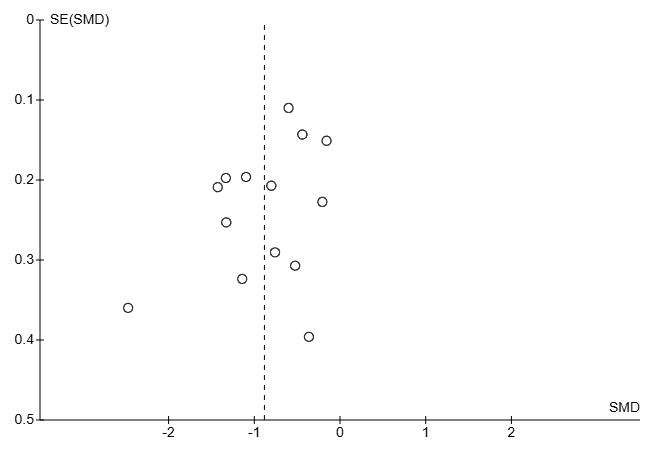


^a^Egger’s test for funnel plot asymmetry (conducted in SPSS): Intercept=-1.40, CI -2.77 to -0.04, P=.04

Figure S15. Funnel plot for depression outcome of the meta-analysis on VR interventions for stress reduction in the general population, generated using RevMan. ^a^
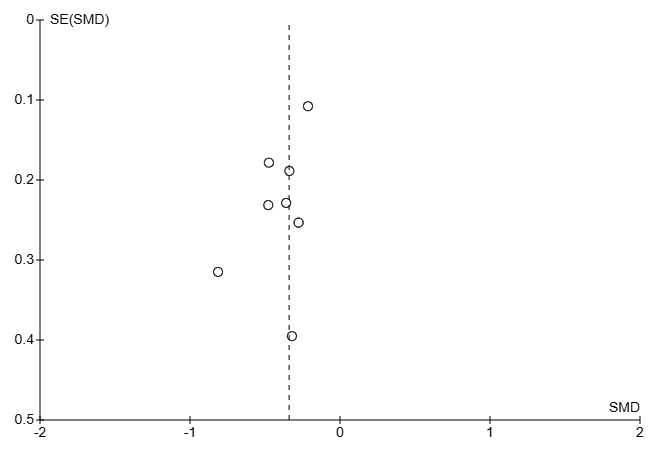


^a^Egger’s test for funnel plot asymmetry (conducted in SPSS): Intercept=-0.53, CI -1.11 to 0.06, P=.07

Figure S16. Funnel plot for restoration outcome of the meta-analysis on VR interventions for stress reduction in the general population, generated using RevMan. ^a^


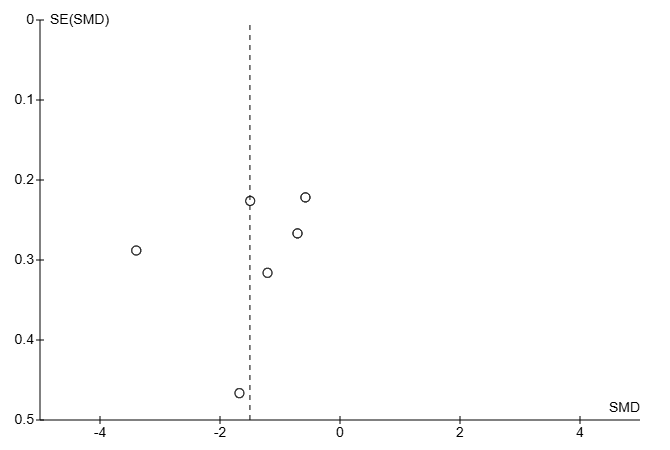


^a^Egger’s test for funnel plot asymmetry (conducted in SPSS): Intercept=-2.23, CI -6.46 to 2.00, P=.22
