# Supplementary material for: Virtual Reality Interventions for Stress Reduction in the General Population: Systematic Review and Meta-Analysis of Randomized Controlled Trials
Source: J Med Internet Res. 2026 May 25;28:e78212. doi: 10.2196/78212 (PMC13200809; doi:10.2196/78212)
Supplement: Multimedia Appendix 7 [file jmir-v28-e78212-s007.docx]

# Multimedia Appendix 7

## Meta regressions performed with the subset of study design factors (32 studies)

Table S1. Multivariate linear regression analysis (R²=0.0%, F_6,25_=0.90; P=.51) for possible predictors of the intervention effectiveness based on study design, performed in the framework of the meta-analysis on VR interventions for stress reduction in general population.^a-c^

| Predictor | Estimate | SE | T | P |
| --- | --- | --- | --- | --- |
|  |  |  |  |  |
| Intercept | -0.97 | 0.42 | -2.31 | =.03 |
| Intervention duration (days) | -0.00 | 0.01 | -0.36 | =.72 |
| Number of VR sessions | 0.02 | 0.02 | 0.89 | =.38 |
| Single VR session length (min) | -0.02 | 0.01 | -1.21 | =.24 |
| Males (%) | 0.01 | 0.01 | 1.02 | =.32 |
| Mean age (years) | 0.01 | 0.01 | 0.54 | =.59 |
| Clinical setting (1,0) ^c^ | 0.07 | 0.38 | 0.18 | =.86 |

^a^Analyses were conducted in IBM SPSS statistics, using a random-effects model with KHSJ correction.

^b^Effect sizes of different VR interventions of one publication were pooled for main analyses and are included as one intervention group [56,65,68,73].

^c^Clinical setting was dummy coded as 1=”clinical setting” and 0=”non-clinical setting”.

Figure S1. Bubble plots of the meta regression analyses for possible predictors of the intervention effectiveness based on study design, performed in the framework of the meta-analysis on VR interventions for stress reduction in general population (n=32), generated in R Studio.

| SMD | 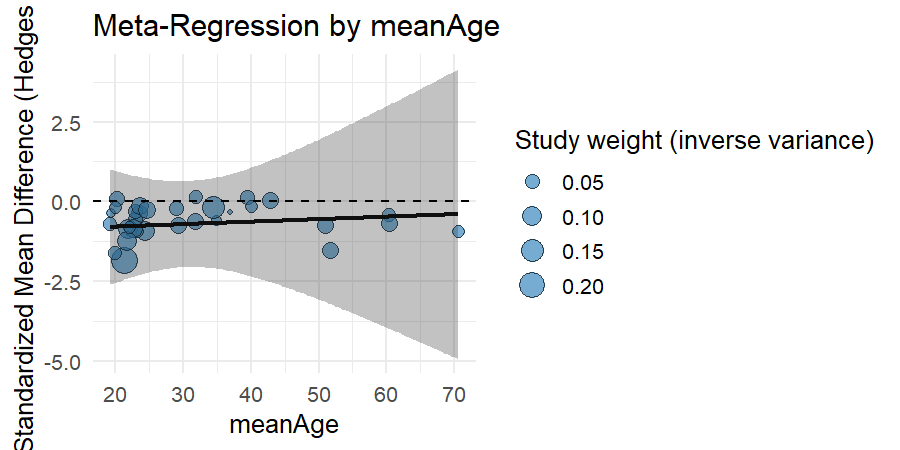 | 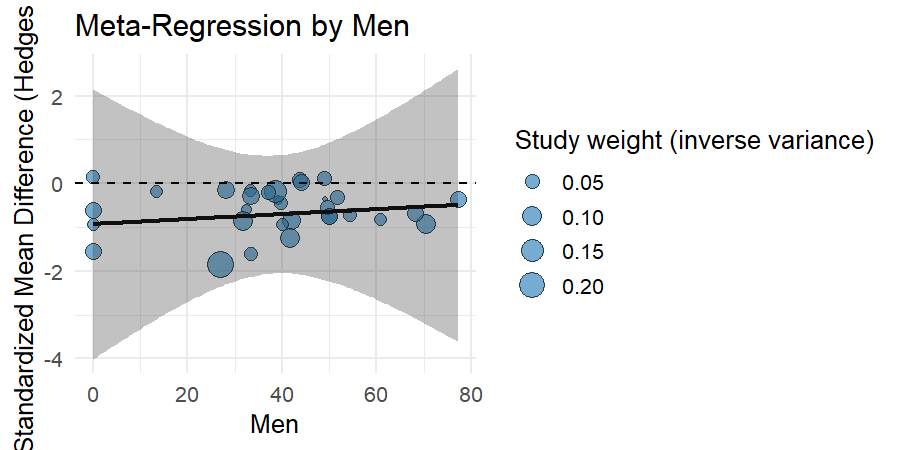 |
| --- | --- | --- |
|  | Mean age (years) | Males (%) |
| SMD | 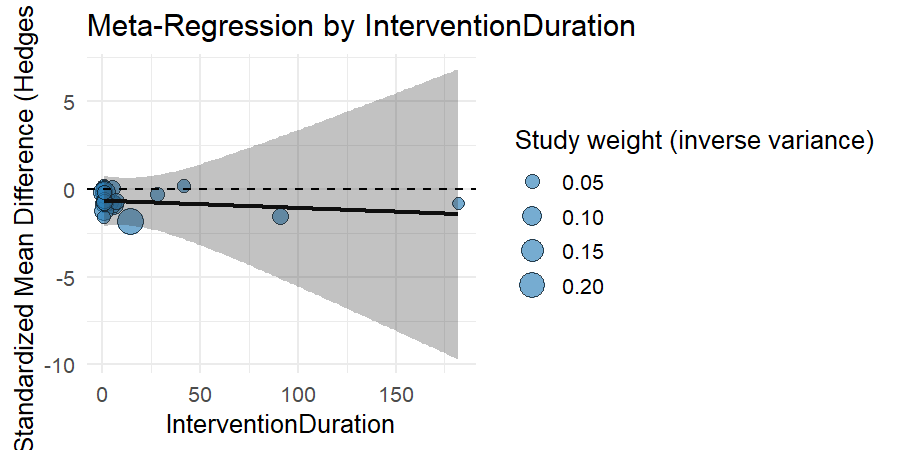 | 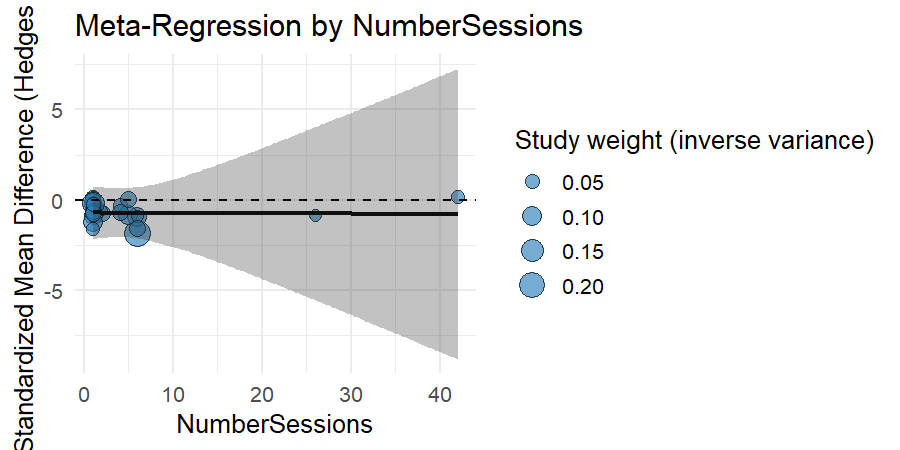 |
|  | Intervention duration (days) | Number of Sessions |
| SMD | 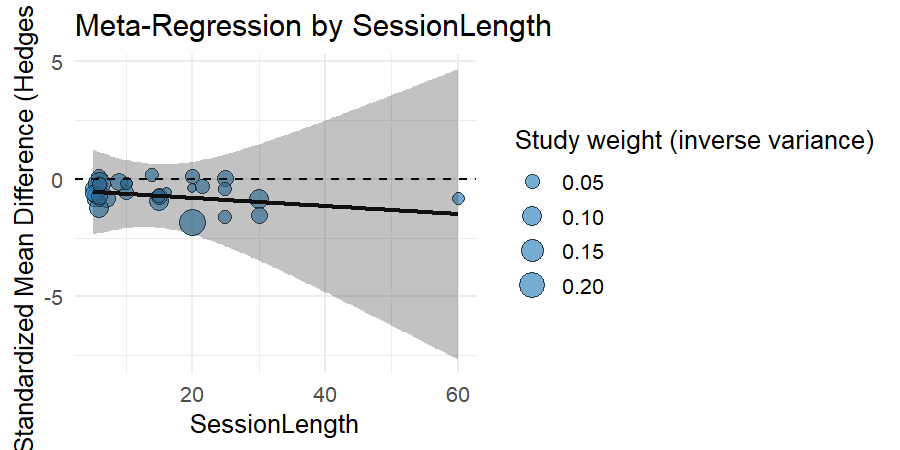 | 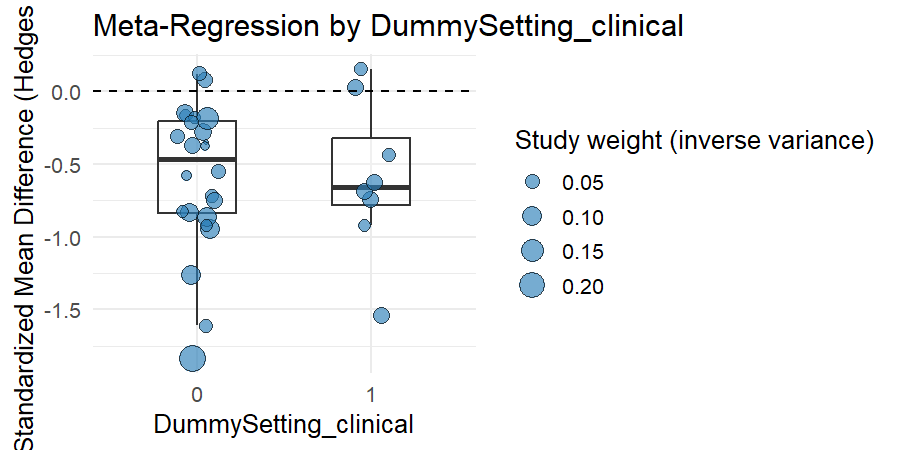 |
|  | Length of single sessions (minutes) | Setting (0=non-clinical; 1=clinical) |

## Meta regressions performed with the subset of content-related and technical aspects (17 studies)

Table S2. Multivariate linear regression analyses (R²=42.9%, F_5,11_=3.43, P=.04) for possible predictors of the intervention effectiveness based on content-related and technical aspects (n=17), performed in the framework of the meta-analysis on VR interventions for stress reduction in general population.^a-e^

| Predictor | estimate | SE | T | P |
| --- | --- | --- | --- | --- |
|  |  |  |  |  |
| Intercept | 1.28 | 1.66 | 0.77 | =.46 |
| Content motion^c^ | -0.15 | 0.23 | -0.64 | =.54 |
| User interactivity^b^ | 0.37 | 0.26 | 1.40 | =.19 |
| Environment realism^d^ | -0.28 | 0.35 | -0.81 | =.43 |
| Refresh rate (Hz) | -0.03 | 0.01 | -2.36 | =.04 |
| FoV^e^ (degree) | -0.00 | 0.02 | -0.13 | =.90 |

^a^Analyses were conducted in IBM SPSS statistics, using a random-effects model with KHSJ correction.

^b^User interactivity was ranked as 1=“low control” (only watching and exploring scene by head movement), 2=“medium control” (option to change perspective while moving in environment by controller or body movement), or 3=“high control” (option to interact with elements due to an interactive task or game).

^c^Content motion was categorized as 1=“no motion” (simple 3D images without moving elements), 2=“low motion” (static scene with some moving elements), or 3=“high motion” (dynamic scene with changing perspective).

^d^Environment realism was dummy coded as 1=”real world recording” and 0=”computer simulation”.

^e^Diagonal field of view (FoV) was calculated by the following formular: $\sqrt{\left( horizontal fov \right)^{2}+(vertical fov)^{2}}$

Figure S2. Bubble plots of the meta regression analyses for possible predictors of the intervention effectiveness based on content-related and technical aspects (n=17), performed in the framework of the meta-analysis on VR interventions for stress reduction in general population, generated in R Studio.

| SMD | 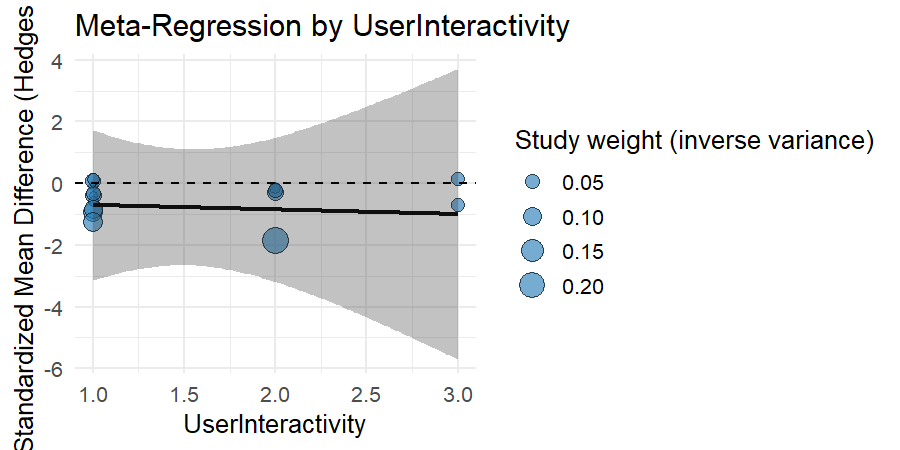 | 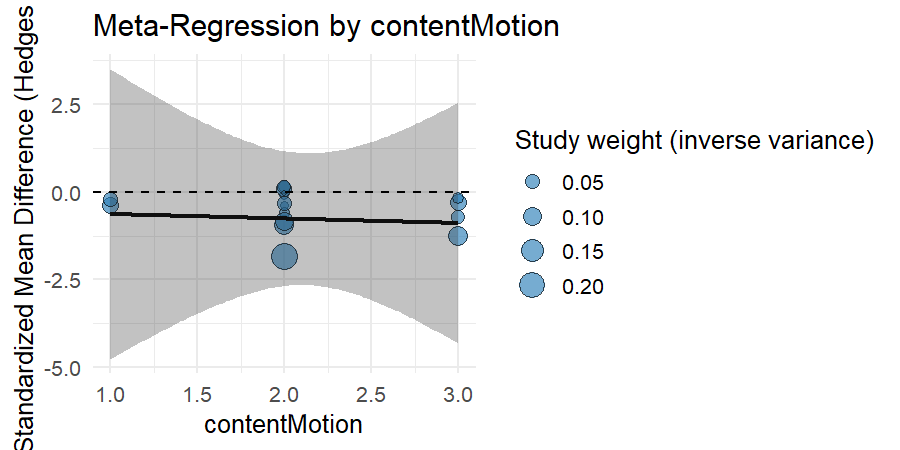 |
| --- | --- | --- |
|  | Level of user interactivity | Level of content motion |
| SMD | 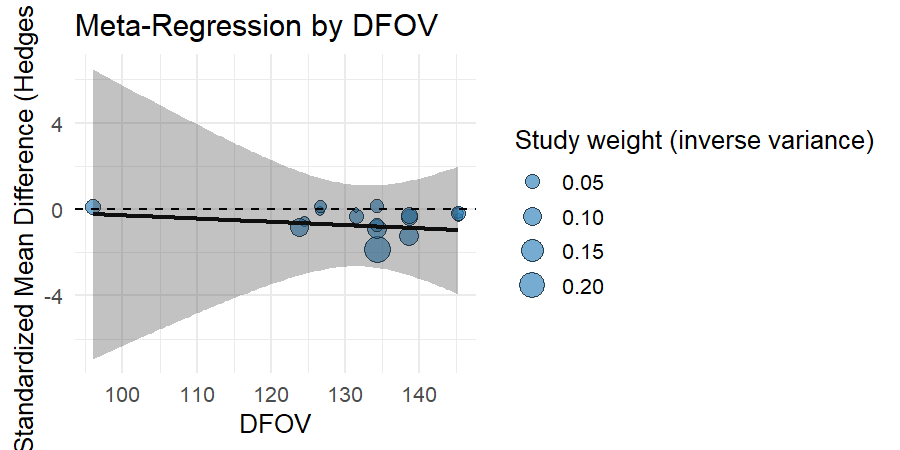 | 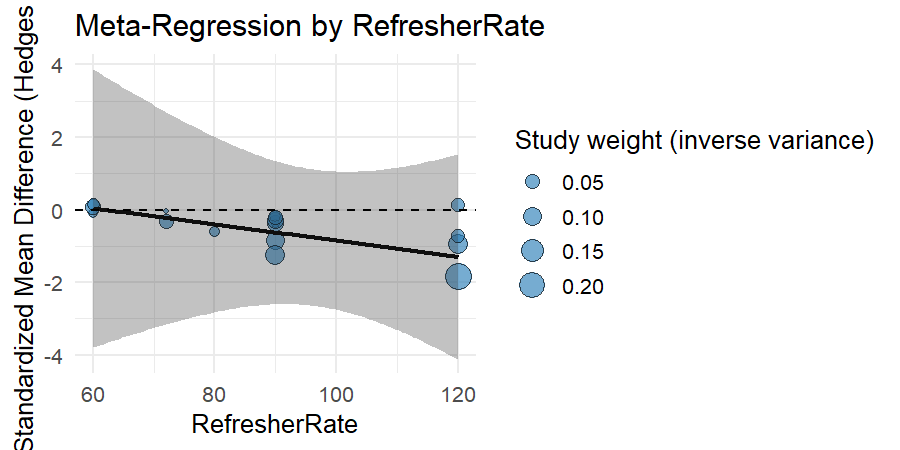 |
|  | FoV (degree) | Refresh rate (Hz) |
| SMD | 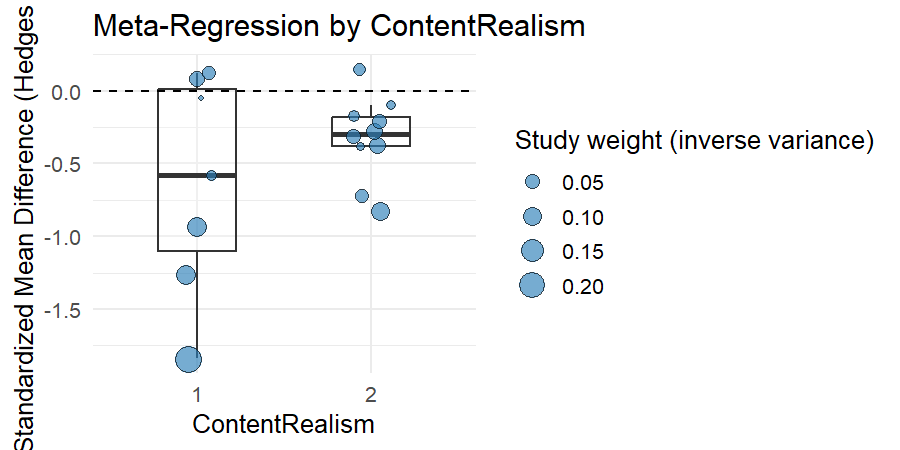 |  |
|  | Environment realism (1=recording; 2=simulation) |  |
